# Patterns of intron gain and conservation in eukaryotic genes

**DOI:** 10.1186/1471-2148-7-192

**Published:** 2007-10-12

**Authors:** Liran Carmel, Igor B Rogozin, Yuri I Wolf, Eugene V Koonin

**Affiliations:** 1 National Center for Biotechnology Information, National Library of Medicine, National Institutes of Health, Bethesda, Maryland 20894, USA.

## Abstract

**Background:**

The presence of introns in protein-coding genes is a universal feature of eukaryotic genome organization, and the genes of multicellular eukaryotes, typically, contain multiple introns, a substantial fraction of which share position in distant taxa, such as plants and animals. Depending on the methods and data sets used, researchers have reached opposite conclusions on the causes of the high fraction of shared introns in orthologous genes from distant eukaryotes. Some studies conclude that shared intron positions reflect, almost entirely, a remarkable evolutionary conservation, whereas others attribute it to parallel gain of introns. To resolve these contradictions, it is crucial to analyze the evolution of introns by using a model that minimally relies on arbitrary assumptions.

**Results:**

We developed a probabilistic model of evolution that allows for variability of intron gain and loss rates over branches of the phylogenetic tree, individual genes, and individual sites. Applying this model to an extended set of conserved eukaryotic genes, we find that parallel gain, on average, accounts for only ~8% of the shared intron positions. However, the distribution of parallel gains over the phylogenetic tree of eukaryotes is highly non-uniform. There are, practically, no parallel gains in closely related lineages, whereas for distant lineages, such as animals and plants, parallel gains appear to contribute up to 20% of the shared intron positions. In accord with these findings, we estimated that ancestral introns have a high probability to be retained in extant genomes, and conversely, that a substantial fraction of extant introns have retained their positions since the early stages of eukaryotic evolution. In addition, the density of sites that are available for intron insertion is estimated to be, approximately, one in seven basepairs.

**Conclusion:**

We obtained robust estimates of the contribution of parallel gain to the observed sharing of intron positions between eukaryotic species separated by different evolutionary distances. The results indicate that, although the contribution of parallel gains varies across the phylogenetic tree, the high level of intron position sharing is due, primarily, to evolutionary conservation. Accordingly, numerous introns appear to persist in the same position over hundreds of millions of years of evolution. This is compatible with recent observations of a negative correlation between the rate of intron gain and coding sequence evolution rate of a gene, suggesting that at least some of the introns are functionally relevant.

## Background

The presence of spliceosomal introns and the concurrent splicing machinery are one of the principal distinctive features of eukaryotic genomes [1-3]. Indeed, even early branching eukaryotes that were once suspected of being intronless have been shown in recent years to posses at least a few introns [4-7]. This suggests that the evolution of introns is tightly linked to the central aspects of the evolution of eukaryotes, and it even has been proposed that introns were the driving force behind the emergence of the eukaryotic nucleus and other features of the eukaryotic cell [8,9].

Thanks to their ubiquity and potential major role in the evolution of eukaryotes, intron evolution has drawn considerable attention [1-3,10]. It is generally accepted that introns are units of evolution such that their presence/absence pattern is a result of stochastic processes of loss and gain. The details of these processes, however, remain elusive. Only in recent years, with the accumulation of genomic data, evolution of introns has become amenable to a systematic, genome-wide analysis. However, different attempts to accomplish such a study led to incongruent conclusions regarding the prevalence, rates, and timing of intron loss and gain during the evolution of eukaryotes [11-18]. Apparently, these discrepancies are due, primarily, to incomplete underlying evolutionary models and biased estimation techniques [10].

Recently, we have obtained more conclusive results by developing a comprehensive probabilistic model of intron evolution, and by compiling a data set that is considerably larger than any one previously used [19,20]. The probabilistic models used so far to study intron evolution can be classified into two groups: branch-specific and gene-specific ones. The branch-specific models assume that the processes of intron gain and loss along a branch are determined only by the properties of the branch, regardless of the particular gene in question [17,18,21]. Conversely, gene-specific models assume that these processes are determined only by the particular gene, independent on the branch in question [12]. Obviously, in reality, the characteristics of intron gain and loss processes vary considerably both across genes and across branches. Thus, each of the models employed thus far seem to describe only one facet of a more complex reality. By contrast, our model allows for the variability of intron gain and loss characteristics with respect to both genes and branches such that any of the previously suggested models can be shown to be a special case of this comprehensive model [20]. Moreover, this model is even more realistic in that it also includes rate variability between sites, with respect to both intron gain and intron loss. In order to estimate the model parameters, we devised an expectation-maximization (EM) algorithm that can also be used to reconstruct ancestral states [22]. Combining this algorithm with the profile likelihood technique allows one, in addition to all of the above, to compute confidence intervals of the model parameters.

Here, we describe, in detail, the developed model of intron evolution and the derivation of an improved version of the EM algorithm used to estimate its parameters. Previously, we have applied this comprehensive model to a set of 391 conserved genes from 19 eukaryotic species, to investigate evolutionary trends in gene structure both at the lineage level [20] and at the gene level [19]. The results of this analysis suggest that introns invaded eukaryotic genomes at early stages of eukaryogenesis in a nearly neutral process. At early times, during periods of major transitions in the eukaryotic evolution that led to population bottlenecks, these introns seem to have vastly proliferated in the ancient genomes. Gradually, a considerable fraction of the introns appear to become involved in various cellular functions, mostly, regulation of gene expression [19].

In the present work, we focus on the process of intron gain, and address the causes of the high level of intron position conservation between eukaryotic taxa. It had been already noticed that many intron positions are shared between distant eukaryotic taxa [11,13]. For example, plants and animals share up to 25% of the intron positions [13]. However, these findings can be explained by either remarkable conservation of ancient introns or by parallel, independent, intron gains in the same positions, or (perhaps, most likely) by a combination of both these factors. The previous analyses that have attempted to quantify the relative contributions of evolutionary conservation and parallel gain in intron position sharing have differed widely, with estimates of the extent of parallel gain ranging from nearly 0% to nearly 100% [11-13,18,23]. Using our probabilistic model, we developed a rigorous measure for assessing the amount of parallel gain of introns. We found that, overall, parallel gain is responsible for ~8% of the shared intron positions, with the rest due to shared ancestry. However, we also demonstrate substantial heterogeneity in the extent of parallel gain, with almost none in closely related lineages, but up to ~20% in distant ones, such as plants and unikonts. On the whole, these results support the notion that intron positions are highly conserved during evolution.

## Results and discussion

### Notation

The primary input component in the study of intron evolution consists of *G *sets of aligned protein-coding sequences of orthologous genes from *S *species. Each nucleotide in these alignments is substituted by 0 or 1 depending on whether or not an intron is present following the respective position. We allow for missing data by using a third symbol (*) to indicate lack of knowledge about the presence or absence of an intron. Consequently, every site in the alignments, called *pattern*, is a vector of length *S *over the alphabet (0,1,*) and is denoted by *ω*. Let Ω be the total number of unique patterns in the entire set of *G *alignments, and let *n*_*gp *_be the count of the number of times pattern *ω*_*p *_(*p *= 1, ..., Ω) is found in the multiple alignment of gene *g*. Assuming that the sites evolve independently, the set *M*_*g *_= (*n*_*g*1_, ..., *n*_*g*Ω_) fully characterizes the multiple alignment of the *g *th gene.

Let *T *be a rooted, bifurcating phylogenetic tree with *S *leaves (terminal nodes), describing the evolutionary relationships between the *S *species above. The total number of nodes in *T *is *N *= 2*S *- 1, and we index them by *t *= 0,1, ..., *N *- 1, with the convention that zero is the root node. The state of node *t *is described by the variable *q*_*t*_, which can take the values 0 and 1 (and * in leaves). We use *V*_*t *_for the set of all leaves such that node *t *is among their ancestors. The entire collection of leaves is, obviously, *V*_0_. The parent node of *t *is denoted P(*t*). We use the notations qtP
 MathType@MTEF@5@5@+=feaafiart1ev1aaatCvAUfKttLearuWrP9MDH5MBPbIqV92AaeXatLxBI9gBaebbnrfifHhDYfgasaacH8akY=wiFfYdH8Gipec8Eeeu0xXdbba9frFj0=OqFfea0dXdd9vqai=hGuQ8kuc9pgc9s8qqaq=dirpe0xb9q8qiLsFr0=vr0=vr0dc8meaabaqaciaacaGaaeqabaqabeGadaaakeaacqWGXbqCdaqhaaWcbaGaemiDaqhabaGaemiuaafaaaaa@30DE@ and VtP
 MathType@MTEF@5@5@+=feaafiart1ev1aaatCvAUfKttLearuWrP9MDH5MBPbIqV92AaeXatLxBI9gBaebbnrfifHhDYfgasaacH8akY=wiFfYdH8Gipec8Eeeu0xXdbba9frFj0=OqFfea0dXdd9vqai=hGuQ8kuc9pgc9s8qqaq=dirpe0xb9q8qiLsFr0=vr0=vr0dc8meaabaqaciaacaGaaeqabaqabeGadaaakeaacqWGwbGvdaqhaaWcbaGaemiDaqhabaGaemiuaafaaaaa@30A8@ for *q*_P(*t*) _and *V*_P(*t*)_, respectively. Analogously, the two direct descendents of the node *t *are denoted L(*t*) and R(*t*), and we use the notations qtL
 MathType@MTEF@5@5@+=feaafiart1ev1aaatCvAUfKttLearuWrP9MDH5MBPbIqV92AaeXatLxBI9gBaebbnrfifHhDYfgasaacH8akY=wiFfYdH8Gipec8Eeeu0xXdbba9frFj0=OqFfea0dXdd9vqai=hGuQ8kuc9pgc9s8qqaq=dirpe0xb9q8qiLsFr0=vr0=vr0dc8meaabaqaciaacaGaaeqabaqabeGadaaakeaacqWGXbqCdaqhaaWcbaGaemiDaqhabaGaemitaWeaaaaa@30D6@, qtR
 MathType@MTEF@5@5@+=feaafiart1ev1aaatCvAUfKttLearuWrP9MDH5MBPbIqV92AaeXatLxBI9gBaebbnrfifHhDYfgasaacH8akY=wiFfYdH8Gipec8Eeeu0xXdbba9frFj0=OqFfea0dXdd9vqai=hGuQ8kuc9pgc9s8qqaq=dirpe0xb9q8qiLsFr0=vr0=vr0dc8meaabaqaciaacaGaaeqabaqabeGadaaakeaacqWGXbqCdaqhaaWcbaGaemiDaqhabaGaemOuaifaaaaa@30E2@, VtL
 MathType@MTEF@5@5@+=feaafiart1ev1aaatCvAUfKttLearuWrP9MDH5MBPbIqV92AaeXatLxBI9gBaebbnrfifHhDYfgasaacH8akY=wiFfYdH8Gipec8Eeeu0xXdbba9frFj0=OqFfea0dXdd9vqai=hGuQ8kuc9pgc9s8qqaq=dirpe0xb9q8qiLsFr0=vr0=vr0dc8meaabaqaciaacaGaaeqabaqabeGadaaakeaacqWGwbGvdaqhaaWcbaGaemiDaqhabaGaemitaWeaaaaa@30A0@, and VtR
 MathType@MTEF@5@5@+=feaafiart1ev1aaatCvAUfKttLearuWrP9MDH5MBPbIqV92AaeXatLxBI9gBaebbnrfifHhDYfgasaacH8akY=wiFfYdH8Gipec8Eeeu0xXdbba9frFj0=OqFfea0dXdd9vqai=hGuQ8kuc9pgc9s8qqaq=dirpe0xb9q8qiLsFr0=vr0=vr0dc8meaabaqaciaacaGaaeqabaqabeGadaaakeaacqWGwbGvdaqhaaWcbaGaemiDaqhabaGaemOuaifaaaaa@30AC@ for *q*_L(*t*)_, *q*_R(*t*)_, *V*_L(*t*)_, and *V*_R(*t*)_, respectively. The branches are indexed by the node into which they are leading, and Δ_*t *_denotes the length (in time units) of the *t *th branch. Hereinafter we assume that the tree topology, as well as all the branch lengths Δ_1_, ..., Δ_*N*-1_, are known.

### The probabilistic model

A bifurcating phylogenetic tree can be viewed as a graphical model depicting the probabilistic model

Pr⁡(q0)∏t=1N−1Pr⁡(qt|qtP).
 MathType@MTEF@5@5@+=feaafiart1ev1aaatCvAUfKttLearuWrP9MDH5MBPbIqV92AaeXatLxBI9gBaebbnrfifHhDYfgasaacH8akY=wiFfYdH8Gipec8Eeeu0xXdbba9frFj0=OqFfea0dXdd9vqai=hGuQ8kuc9pgc9s8qqaq=dirpe0xb9q8qiLsFr0=vr0=vr0dc8meaabaqaciaacaGaaeqabaqabeGadaaakeaacyGGqbaucqGGYbGCcqGGOaakcqWGXbqCdaWgaaWcbaGaeGimaadabeaakiabcMcaPmaarahabaGagiiuaaLaeiOCaiNaeiikaGIaemyCae3aaSbaaSqaaiabdsha0bqabaGccqGG8baFcqWGXbqCdaqhaaWcbaGaemiDaqhabaGaemiuaafaaOGaeiykaKcaleaacqWG0baDcqGH9aqpcqaIXaqmaeaacqWGobGtcqGHsislcqaIXaqma0Gaey4dIunakiabc6caUaaa@4A21@

We use the notation *π*_*i *_= Pr(*q*_0 _= *i*) to describe the prior probability of the root, and *T*_*ij*_(*g, t*) = Pr(*q_t_*= *j*|qtP
 MathType@MTEF@5@5@+=feaafiart1ev1aaatCvAUfKttLearuWrP9MDH5MBPbIqV92AaeXatLxBI9gBaebbnrfifHhDYfgasaacH8akY=wiFfYdH8Gipec8Eeeu0xXdbba9frFj0=OqFfea0dXdd9vqai=hGuQ8kuc9pgc9s8qqaq=dirpe0xb9q8qiLsFr0=vr0=vr0dc8meaabaqaciaacaGaaeqabaqabeGadaaakeaacqWGXbqCdaqhaaWcbaGaemiDaqhabaGaemiuaafaaaaa@30DE@ = *i, g*) to describe the transition probability for gene *g *along branch *t*. In our model, we assume that this transition probability depends on both the gene and the branch, and that it takes the explicit form

T(g,t)=(1−ξt(1−e−ηgΔt)ξt(1−e−ηgΔt)1−(1−φt)e−θgΔt(1−φt)e−θgΔt).
 MathType@MTEF@5@5@+=feaafiart1ev1aaatCvAUfKttLearuWrP9MDH5MBPbIqV92AaeXatLxBI9gBaebbnrfifHhDYfgasaacH8akY=wiFfYdH8Gipec8Eeeu0xXdbba9frFj0=OqFfea0dXdd9vqai=hGuQ8kuc9pgc9s8qqaq=dirpe0xb9q8qiLsFr0=vr0=vr0dc8meaabaqaciaacaGaaeqabaqabeGadaaakeaacqWGubavcqGGOaakcqWGNbWzcqGGSaalcqWG0baDcqGGPaqkcqGH9aqpdaqadaqaauaabeqaciaaaeaacqaIXaqmcqGHsisliiGacqWF+oaEdaWgaaWcbaGaemiDaqhabeaakiabcIcaOiabigdaXiabgkHiTiabdwgaLnaaCaaaleqabaGaeyOeI0Iae83TdG2aaSbaaWqaaiabdEgaNbqabaWccqqHuoardaWgaaadbaGaemiDaqhabeaaaaGccqGGPaqkaeaacqWF+oaEdaWgaaWcbaGaemiDaqhabeaakiabcIcaOiabigdaXiabgkHiTiabdwgaLnaaCaaaleqabaGaeyOeI0Iae83TdG2aaSbaaWqaaiabdEgaNbqabaWccqqHuoardaWgaaadbaGaemiDaqhabeaaaaGccqGGPaqkaeaacqaIXaqmcqGHsislcqGGOaakcqaIXaqmcqGHsislcqWFgpGzdaWgaaWcbaGaemiDaqhabeaakiabcMcaPiabdwgaLnaaCaaaleqabaGaeyOeI0Iae8hUde3aaSbaaWqaaiabdEgaNbqabaWccqqHuoardaWgaaadbaGaemiDaqhabeaaaaaakeaacqGGOaakcqaIXaqmcqGHsislcqWFgpGzdaWgaaWcbaGaemiDaqhabeaakiabcMcaPiabdwgaLnaaCaaaleqabaGaeyOeI0Iae8hUde3aaSbaaWqaaiabdEgaNbqabaWccqqHuoardaWgaaadbaGaemiDaqhabeaaaaaaaaGccaGLOaGaayzkaaGaeiOla4caaa@7914@

Here, *η*_*g *_and *θ*_*g *_are non-negative parameters which determine, respectively, the intron gain and loss rates per site for gene *g*. That is, along branch *t *the gene's contribution to intron gain and retention probabilities per site is 1−e−ηgΔt
 MathType@MTEF@5@5@+=feaafiart1ev1aaatCvAUfKttLearuWrP9MDH5MBPbIqV92AaeXatLxBI9gBaebbnrfifHhDYfgasaacH8akY=wiFfYdH8Gipec8Eeeu0xXdbba9frFj0=OqFfea0dXdd9vqai=hGuQ8kuc9pgc9s8qqaq=dirpe0xb9q8qiLsFr0=vr0=vr0dc8meaabaqaciaacaGaaeqabaqabeGadaaakeaacqaIXaqmcqGHsislcqWGLbqzdaahaaWcbeqaaiabgkHiTGGaciab=D7aOnaaBaaameaacqWGNbWzaeqaaSGaeuiLdq0aaSbaaWqaaiabdsha0bqabaaaaaaa@373C@ and e−θgΔt
 MathType@MTEF@5@5@+=feaafiart1ev1aaatCvAUfKttLearuWrP9MDH5MBPbIqV92AaeXatLxBI9gBaebbnrfifHhDYfgasaacH8akY=wiFfYdH8Gipec8Eeeu0xXdbba9frFj0=OqFfea0dXdd9vqai=hGuQ8kuc9pgc9s8qqaq=dirpe0xb9q8qiLsFr0=vr0=vr0dc8meaabaqaciaacaGaaeqabaqabeGadaaakeaacqWGLbqzdaahaaWcbeqaaiabgkHiTGGaciab=H7aXnaaBaaameaacqWGNbWzaeqaaSGaeuiLdq0aaSbaaWqaaiabdsha0bqabaaaaaaa@3569@, respectively. We assume that each branch is characterized by an intrinsic *branch-specific intron gain coefficient*, *ξ*_*t*_, as well as an intrinsic *branch-specific intron loss coefficient*, *φ*_*t*_, both of which are bounded, 0 ≤ *ξ*_*t*_, *φ*_*t *_≤ 1.

In other fields of molecular evolution, it had been long realized that the precision of the analysis significantly improves if one allows for rate variability across sites [24-26]. Typically, such rate variability is modeled by introducing a *rate variable*, *r*, which scales, for each site, the time units of the phylogenetic tree, Δ_*t *_← *r*·Δ_*t*_. This rate variable is a random variable that is distributed according to a distribution function with non-negative domain and unit mean, typically, the unit-mean gamma distribution. The rate variability captures rate variations among sites of the same gene. Specifically, there are fast-evolving sites (*r *>> 1), as well as slow-evolving ones (*r *<< 1). In our model of intron evolution, we extend this idea by assuming that the gain and loss processes are subject to rate variability, independently of each other. Hence, a site can have any combination of gain and loss rates, for example, it can be fast to gain introns but slow to lose them. To implement this approach, we use two independent rate variables, *r*^*η *^and *r*^*θ*^, that are used to scale, for each site, the gene-specific gain rate, *η*_*g *_← *r*^*η*^·*η*_*g*_, and the gene-specific loss rate, *θ*_*g *_← *r*^*θ*^·*θ*_*g*_, respectively. We further assume that the distributions of these rate variables are independent of the genes, and are explicitly given by

rη~νδ(η)+(1−ν)Γ(η;λη)rθ~Γ(θ;λθ).
 MathType@MTEF@5@5@+=feaafiart1ev1aaatCvAUfKttLearuWrP9MDH5MBPbIqV92AaeXatLxBI9gBaebbnrfifHhDYfgasaacH8akY=wiFfYdH8Gipec8Eeeu0xXdbba9frFj0=OqFfea0dXdd9vqai=hGuQ8kuc9pgc9s8qqaq=dirpe0xb9q8qiLsFr0=vr0=vr0dc8meaabaqaciaacaGaaeqabaqabeGadaaakqaabeqaaiabdkhaYnaaCaaaleqabaacciGae83TdGgaaOGaeiOFa4Nae8xVd4Mae8hTdqMaeiikaGIae83TdGMaeiykaKIaey4kaSIaeiikaGIaeGymaeJaeyOeI0Iae8xVd4MaeiykaKIaeu4KdCKaeiikaGIae83TdGMaei4oaSJae83UdW2aaSbaaSqaaiab=D7aObqabaGccqGGPaqkaeaacqWGYbGCdaahaaWcbeqaaiab=H7aXbaakiabc6ha+jabfo5ahjabcIcaOiab=H7aXjabcUda7iab=T7aSnaaBaaaleaacqWF4oqCaeqaaOGaeiykaKIaeiOla4caaaa@56C5@

Here, Γ(*x*; *λ*) is the unit-mean gamma distribution of the variable *x *with the shape parameter *λ*, *δ*(*x*) is the Dirac delta-function, and *ν *is the fraction of sites that are assumed to have zero gain rate. The existence of these *zero sites *reflects the notion that introns cannot be gained at any location within genes, but rather are preferentially inserted at specific locations, contingent on particular sequence motifs known as proto-splice sites [27-29], the density of other introns in the neighborhood, the chromatin exposure, and more. According to this interpretation, 1 - *ν *measures the density of potential intron insertion sites. Importantly, using the same value of *ν *for the entire tree does not mean that the proto-splice sites are constant throughout the evolution, or are identical for different lineages. It only means that, on the average, the fraction of potential insertion sites, whatever is their concrete nature, is similar across the lineages throughout the course of eukaryotic evolution. The incorporation of such invariant sites in a rate variability model appears natural for intron evolution and has proved beneficial also in other fields of molecular evolution [30-32]. By contrast, intron loss does not have an invariant counterpart because the assumption is that, once an intron is gained, it can always be lost. Therefore, the loss rate variable is assumed to be distributed according to a gamma distribution, which is by far the most popular distribution for describing rate variability [24,33].

In practice, the rate distributions in (3) are rendered discrete [34]. We assume that the gain rate variable can take *K*_*η *_discrete values r1η=0,r2η,…,rKηη
 MathType@MTEF@5@5@+=feaafiart1ev1aaatCvAUfKttLearuWrP9MDH5MBPbIqV92AaeXatLxBI9gBaebbnrfifHhDYfgasaacH8akY=wiFfYdH8Gipec8Eeeu0xXdbba9frFj0=OqFfea0dXdd9vqai=hGuQ8kuc9pgc9s8qqaq=dirpe0xb9q8qiLsFr0=vr0=vr0dc8meaabaqaciaacaGaaeqabaqabeGadaaakeaacqWGYbGCdaqhaaWcbaGaeGymaedabaacciGae83TdGgaaOGaeyypa0JaeGimaaJaeiilaWIaemOCai3aa0baaSqaaiabikdaYaqaaiab=D7aObaakiabcYcaSiablAciljabcYcaSiabdkhaYnaaDaaaleaacqWGlbWsdaWgaaadbaGae83TdGgabeaaaSqaaiab=D7aObaaaaa@4125@ with probabilities f1η=ν,f2η…,fKηη
 MathType@MTEF@5@5@+=feaafiart1ev1aaatCvAUfKttLearuWrP9MDH5MBPbIqV92AaeXatLxBI9gBaebbnrfifHhDYfgasaacH8akY=wiFfYdH8Gipec8Eeeu0xXdbba9frFj0=OqFfea0dXdd9vqai=hGuQ8kuc9pgc9s8qqaq=dirpe0xb9q8qiLsFr0=vr0=vr0dc8meaabaqaciaacaGaaeqabaqabeGadaaakeaacqWGMbGzdaqhaaWcbaGaeGymaedabaacciGae83TdGgaaOGaeyypa0Jae8xVd4MaeiilaWIaemOzay2aa0baaSqaaiabikdaYaqaaiab=D7aObaakiablAciljabcYcaSiabdAgaMnaaDaaaleaacqWGlbWsdaWgaaadbaGae83TdGgabeaaaSqaaiab=D7aObaaaaa@40C2@ such that ∑k=1Kηfkη=1
 MathType@MTEF@5@5@+=feaafiart1ev1aaatCvAUfKttLearuWrP9MDH5MBPbIqV92AaeXatLxBI9gBaebbnrfifHhDYfgasaacH8akY=wiFfYdH8Gipec8Eeeu0xXdbba9frFj0=OqFfea0dXdd9vqai=hGuQ8kuc9pgc9s8qqaq=dirpe0xb9q8qiLsFr0=vr0=vr0dc8meaabaqaciaacaGaaeqabaqabeGadaaakeaadaaeWaqaaiabdAgaMnaaDaaaleaacqWGRbWAaeaaiiGacqWF3oaAaaaabaGaem4AaSMaeyypa0JaeGymaedabaGaem4saS0aaSbaaWqaaiab=D7aObqabaaaniabggHiLdGccqGH9aqpcqaIXaqmaaa@3B7F@. Analogously, we assume that the loss rate variable can take *K*_*θ *_discrete values r1θ,…,rKθθ
 MathType@MTEF@5@5@+=feaafiart1ev1aaatCvAUfKttLearuWrP9MDH5MBPbIqV92AaeXatLxBI9gBaebbnrfifHhDYfgasaacH8akY=wiFfYdH8Gipec8Eeeu0xXdbba9frFj0=OqFfea0dXdd9vqai=hGuQ8kuc9pgc9s8qqaq=dirpe0xb9q8qiLsFr0=vr0=vr0dc8meaabaqaciaacaGaaeqabaqabeGadaaakeaacqWGYbGCdaqhaaWcbaGaeGymaedabaacciGae8hUdehaaOGaeiilaWIaeSOjGSKaeiilaWIaemOCai3aa0baaSqaaiabdUealnaaBaaameaacqWF4oqCaeqaaaWcbaGae8hUdehaaaaa@3A32@ with probabilities f1θ,…,fKθθ
 MathType@MTEF@5@5@+=feaafiart1ev1aaatCvAUfKttLearuWrP9MDH5MBPbIqV92AaeXatLxBI9gBaebbnrfifHhDYfgasaacH8akY=wiFfYdH8Gipec8Eeeu0xXdbba9frFj0=OqFfea0dXdd9vqai=hGuQ8kuc9pgc9s8qqaq=dirpe0xb9q8qiLsFr0=vr0=vr0dc8meaabaqaciaacaGaaeqabaqabeGadaaakeaacqWGMbGzdaqhaaWcbaGaeGymaedabaacciGae8hUdehaaOGaeiilaWIaeSOjGSKaeiilaWIaemOzay2aa0baaSqaaiabdUealnaaBaaameaacqWF4oqCaeqaaaWcbaGae8hUdehaaaaa@3A02@ such that ∑k=1Kθfkθ=1
 MathType@MTEF@5@5@+=feaafiart1ev1aaatCvAUfKttLearuWrP9MDH5MBPbIqV92AaeXatLxBI9gBaebbnrfifHhDYfgasaacH8akY=wiFfYdH8Gipec8Eeeu0xXdbba9frFj0=OqFfea0dXdd9vqai=hGuQ8kuc9pgc9s8qqaq=dirpe0xb9q8qiLsFr0=vr0=vr0dc8meaabaqaciaacaGaaeqabaqabeGadaaakeaadaaeWaqaaiabdAgaMnaaDaaaleaacqWGRbWAaeaaiiGacqWF4oqCaaaabaGaem4AaSMaeyypa0JaeGymaedabaGaem4saS0aaSbaaWqaaiab=H7aXbqabaaaniabggHiLdGccqGH9aqpcqaIXaqmaaa@3B93@. For a particular gain rate value rkη
 MathType@MTEF@5@5@+=feaafiart1ev1aaatCvAUfKttLearuWrP9MDH5MBPbIqV92AaeXatLxBI9gBaebbnrfifHhDYfgasaacH8akY=wiFfYdH8Gipec8Eeeu0xXdbba9frFj0=OqFfea0dXdd9vqai=hGuQ8kuc9pgc9s8qqaq=dirpe0xb9q8qiLsFr0=vr0=vr0dc8meaabaqaciaacaGaaeqabaqabeGadaaakeaacqWGYbGCdaqhaaWcbaGaem4AaSgabaacciGae83TdGgaaaaa@3158@, we denote the actual gain rate rkη
 MathType@MTEF@5@5@+=feaafiart1ev1aaatCvAUfKttLearuWrP9MDH5MBPbIqV92AaeXatLxBI9gBaebbnrfifHhDYfgasaacH8akY=wiFfYdH8Gipec8Eeeu0xXdbba9frFj0=OqFfea0dXdd9vqai=hGuQ8kuc9pgc9s8qqaq=dirpe0xb9q8qiLsFr0=vr0=vr0dc8meaabaqaciaacaGaaeqabaqabeGadaaakeaacqWGYbGCdaqhaaWcbaGaem4AaSgabaacciGae83TdGgaaaaa@3158@·*η*_*g *_by *η*_*kg*_. Similarly, for a particular loss rate value rkθ
 MathType@MTEF@5@5@+=feaafiart1ev1aaatCvAUfKttLearuWrP9MDH5MBPbIqV92AaeXatLxBI9gBaebbnrfifHhDYfgasaacH8akY=wiFfYdH8Gipec8Eeeu0xXdbba9frFj0=OqFfea0dXdd9vqai=hGuQ8kuc9pgc9s8qqaq=dirpe0xb9q8qiLsFr0=vr0=vr0dc8meaabaqaciaacaGaaeqabaqabeGadaaakeaacqWGYbGCdaqhaaWcbaGaem4AaSgabaacciGae8hUdehaaaaa@3162@, we denote the actual loss rate rkθ
 MathType@MTEF@5@5@+=feaafiart1ev1aaatCvAUfKttLearuWrP9MDH5MBPbIqV92AaeXatLxBI9gBaebbnrfifHhDYfgasaacH8akY=wiFfYdH8Gipec8Eeeu0xXdbba9frFj0=OqFfea0dXdd9vqai=hGuQ8kuc9pgc9s8qqaq=dirpe0xb9q8qiLsFr0=vr0=vr0dc8meaabaqaciaacaGaaeqabaqabeGadaaakeaacqWGYbGCdaqhaaWcbaGaem4AaSgabaacciGae8hUdehaaaaa@3162@·*θ*_*g *_by *θ*_*kg*_.

For notational clarity, we aggregate the model's parameters into a small number of sets. To this end, let Ξ_*t *_= {*ξ*_*t*_, *φ*_*t*_} be the set of parameters that are specific to branch *t*, and let Ξ = (Ξ_1_, ..., Ξ_*N*-1_) be the set of all branch-specific parameters. Similarly, let Ψ_*g *_= (*η*_*g*_, *θ*_*g*_) be the set of parameters that are specific for gene *g*, and let Ψ = (Ψ_1_, ..., Ψ_*G*_) be the set of all gene-specific parameters. Additionally, we denote by Λ = (*π*_0_, *ν*, *λ*_*η*_, *λ*_*θ*_) the "global" parameters that determine the rate variability and the prior probability of an intron absent in the root. When the distinction between the different sets of parameters is irrelevant, we shall use Θ = (Ξ, Ψ, Λ) as the set of all the model's parameters. We achieve further succinctness in notations by denoting the actual gene-specific rate values for particular values rkη
 MathType@MTEF@5@5@+=feaafiart1ev1aaatCvAUfKttLearuWrP9MDH5MBPbIqV92AaeXatLxBI9gBaebbnrfifHhDYfgasaacH8akY=wiFfYdH8Gipec8Eeeu0xXdbba9frFj0=OqFfea0dXdd9vqai=hGuQ8kuc9pgc9s8qqaq=dirpe0xb9q8qiLsFr0=vr0=vr0dc8meaabaqaciaacaGaaeqabaqabeGadaaakeaacqWGYbGCdaqhaaWcbaGaem4AaSgabaacciGae83TdGgaaaaa@3158@ and rk'θ
 MathType@MTEF@5@5@+=feaafiart1ev1aaatCvAUfKttLearuWrP9MDH5MBPbIqV92AaeXatLxBI9gBaebbnrfifHhDYfgasaacH8akY=wiFfYdH8Gipec8Eeeu0xXdbba9frFj0=OqFfea0dXdd9vqai=hGuQ8kuc9pgc9s8qqaq=dirpe0xb9q8qiLsFr0=vr0=vr0dc8meaabaqaciaacaGaaeqabaqabeGadaaakeaacqWGYbGCdaqhaaWcbaGaem4AaSMaei4jaCcabaacciGae8hUdehaaaaa@3238@ of the rate variables as Ψ_*kk*'*g *_= (*η*_*kg*_, *θ*_*k*'*g*_).

### The Expectation-Maximization algorithm

We estimate the parameters of the model using maximum likelihood. As the probability model includes observed random variables (state of the tree leaves) as well as hidden random variables (state of internal nodes in the tree and value of actual rate variables), the expectation-maximization algorithm is a natural tool to use [35]. This is a hill climbing iterative algorithm that requires two steps in each iteration – the expectation (E) step followed by the maximization (M) step. The details of the algorithm are given under Methods.

The total number of parameters in the model is 2*G *+ 4*S*, where *G *is the number of genes and *S *is the number of species. For data sets in the hundreds of genes, this sums up to >1000 parameters. For infinite data, maximum likelihood estimators are known to be nonbiased and efficient. However, as each gene typically goes through a small number of intron-related events during its lifetime, the information content of our data is limited, and cannot straightforwardly support the estimation of such a large number of parameter. To overcome this, we adopt a two-phase approach in the data analysis. In the first phase, that we denoted *homogeneous evolution*, it is assumed that all the genes have identical intron gain and loss rates, formally, that *θ*_*g *_= *θ*_0 _and *η*_*g *_= *η*_0 _for any gene *g*. In the second phase, denoted *heterogeneous evolution*, all the global and branch-specific parameters are fixed, which allows for estimation of the gene-specific parameters *θ*_*g *_and *η*_*g *_that can now take different values for different genes. Except for the rate variability within genes, the model of evolution under the homogeneous phase resembles the branch-specific models, and consequently the EM algorithm used in this part has similar structure to the EM algorithm developed by Nguyen *et al*. [18,36].

Using simulations, we showed that this approach yields highly accurate evolutionary reconstructions: a relative error of 1%, 3%, and 11% in estimating the number of introns in internal nodes, the number of loss events along each branch, and the number of gain events along each branch, respectively [20]. The current analysis is pattern-centered rather than gene-centered and thus we have used the results of the homogeneous phase only. While slightly less accurate than the heterogeneous counterparts, the reconstructions after the homogeneous phase are still highly accurate: a relative error of 2%, 4%, and 12% in estimating the number of introns in internal nodes, the number of losses along each branch, and the number of gains along each branch, respectively [20]. Also notable is that, apart from the fraction of zero sites *v*, the rate variability within genes can be ignored without having any significant effect on the results (Ref. [20] and Additional file 1), indicating that those sites that are capable of gaining introns do so at comparable rates.

The EM algorithm was applied to the set of 391 orthologous genes from 19 eukaryotic species ([20] and see Methods), under the homogeneous evolution assumption, and the profile likelihood technique was used to estimate the 95% confidence interval for each parameter (Additional file 1). In computing the confidence intervals for a particular parameter, we allow all other parameters to vary. Sometimes, different combinations of parameters yield similar likelihood values, an observation that has already been made formal for simpler models [18]. This occasionally leads to wide single-parameter confidence intervals, especially for the intron gain and loss rates of deep nodes. Importantly, this does not reflect similarly large errors in the ensuing inference, as was demonstrated by an exhaustive simulation study [19,20].

### One in 7 nucleotides is a potential intron insertion site

The maximum-likelihood estimate for the fraction of zero sites is *ν *= 0.862, suggesting a potential intron insertion site every 7 nucleotides. Taking into account the 95% confidence interval for *ν*, [0.631, 0.928], the density of potential insertion sites is estimated to be 1 site per 3-14 nucleotides. Based on a smaller data set, Nguyen *et al*. [18] computed a 95% confidence interval of 1 potential insertion site in 9–14 nucleotides. Although this estimate falls within our confidence interval, our results suggest, generally, a denser population of potential insertion sites. The present estimate is also compatible with the high density of intron insertion sites (one site per 9.7 nucleotides) observed for some large protein families, e.g., small G proteins [37]. The problem of estimating the number of zero sites is of fundamental importance in maximum likelihood techniques, so both Csuros [17] and Nguyen *et al*. [18] developed different heuristic procedure to handle it. We took the alternative and, arguably, more natural approach of integrating this estimate into the model as part of the gain rate variability distribution (see above) such that additional, *ad hoc *computations are not required.

One should be cautious about identifying potential intron insertion sites with proto-splice sites. More realistically, the density of potential insertion sites reflects the overall average (across species) of the impact of multiple factors such as differential tendencies to be inserted into different proto-splice sites, local densities of pre-existing introns, and degree of chromatin exposure. Furthermore, this density estimation strongly depends on the specific data set as the parameter *ν *"absorbs" the information on zero sites. Thus, if intronless genes are added to the data, only this parameter will be heavily affected.

With the total number of sites *N*_0 _= 289,902 our calculations yield 39,962 sites that can gain introns (95% confidence interval 20,891 to 106,901). Accordingly, the 5,755 sites that are actually occupied by introns in the genes analyzed here comprise 14.4% of all sites that can potentially gain introns (95% confidence interval 5.4% to 27.5%). Thus, even when data from 19 species are combined, the density of the sites actually occupied by introns is still far below the density of sites capable of hosting introns, which emphasizes the inadequacy of any analysis that considers only those sites in which introns are, actually, observed.

### The number of shared intron positions is much greater than expected by chance

**Figure 1:**
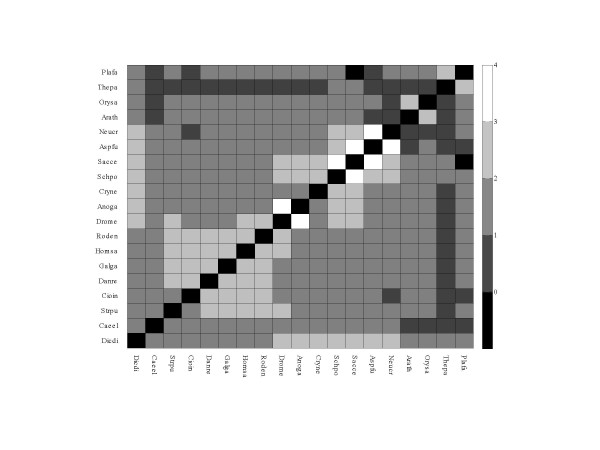
Conservation of intron positions between eukaryotic species. The scale to the right shows the pairwise conservation level of intron positions, measured as the log-ratio of the observed number of shared positions to the number expected by chance (see text). The expected value of this ratio is 0, so the positive values indicate an excess of shared intron positions, and the negative values indicate an unexpected deficit of such positions. Species and lineage abbreviations: Anoga (*Anopheles gambiae*), Arath (*Arabidopsis thaliana*), Aspfu (*Aspergillus fumigatus*), Caeel (*Caenorhabditis elegans*), Cioin (*Ciona intestinalis*), Cryne (*Cryptococcus neoformans*), Danre (*Danio rerio*), Dicdi (*Dictyostelium discoideum*), Drome (*Drosophila melanogaster*), Galga (*Gallus gallus*), Homsa (*Homo sapiens*), Neucr (*Neurospora crassa*), Orysa (*Oryza sativa*), Plafa (*Plasmodium falciparum*), Sacce (*Saccharomyces cerevisiae*), Schpo (*Schizosaccharomyces pombe*), Strpu (*Strongylocentrotus purpuratus*), Thepa (Theileria parva), roden (*Mus musculus *and *Rattus norvegicus *combined).

Numerous intron positions are shared even between taxa that had diverged during the early stages of the eukaryotic evolution [11,13]. To quantify this conservation more precisely and to estimate how surprising it is, compared to the random expectation, we propose the following measure: let *N*_*i *_and *N*_*j *_be the number of intron positions in species *i *and *j*, respectively; let *S*_*ij *_be the number of intron positions shared by the two species; and let *N *be the total number of sites capable of gaining introns. Then, approximately, we would expect that *N*_*i*_*N*_*j*_/*N *positions will be shared between the two species by chance alone. We define the *conservation level*

cij=log⁡(NSijNiNj)
 MathType@MTEF@5@5@+=feaafiart1ev1aaatCvAUfKttLearuWrP9MDH5MBPbIqV92AaeXatLxBI9gBaebbnrfifHhDYfgasaacH8akY=wiFfYdH8Gipec8Eeeu0xXdbba9frFj0=OqFfea0dXdd9vqai=hGuQ8kuc9pgc9s8qqaq=dirpe0xb9q8qiLsFr0=vr0=vr0dc8meaabaqaciaacaGaaeqabaqabeGadaaakeaacqWGJbWydaWgaaWcbaGaemyAaKMaemOAaOgabeaakiabg2da9iGbcYgaSjabc+gaVjabcEgaNnaabmaabaWaaSaaaeaacqWGobGtcqWGtbWudaWgaaWcbaGaemyAaKMaemOAaOgabeaaaOqaaiabd6eaonaaBaaaleaacqWGPbqAaeqaaOGaemOta40aaSbaaSqaaiabdQgaQbqabaaaaaGccaGLOaGaayzkaaaaaa@4256@

as the log-ratio between the observed number and the number expected by chance. Positive values designate that *S*_*ij *_exceeds random expectation, whereas negative values indicate that *S*_*ij *_is below expectation. Even if we take for *N *its lower 95% confidence interval bound of 20,891 (see the preceding section), almost all pairs show a positive value, namely, a greater than random number of shared positions (Fig. 1). The only exception is the pair *S. cerevisiae *– *P. falciparum *(the two most intron-poor species in our set) that do not have even a single shared position.

The number of positions shared by chance between two species is a random variable distributed, approximately, according to a binomial distribution with the probability of success *p *= *N*_*i*_*N*_*j*_/*N*^2^, and *N *experiments. Therefore, it is easy to associate a p-value with any observed number of shared positions, *S*_*ij *_measuring how improbable it is to obtain by chance this value or a greater one. An overall significance level of 0.05 is equivalent to a Bonfferoni-corrected significance level of 0.0003 (overly conservative as we assume that all pairs are independent). The calculations indicate that only 20 species pairs out of 171, all involving the intron-poor *S. cerevisiae *or one of the apicomplexans, had a number of shared positions that was indistinguishable from the random expectation; all other pairs had a significant excess of shared intron positions (Additional file 2).

### Twelve out of thirteen shared intron positions reflect common ancestry

The significant excess of shared intron positions over the random expectation can be explained by one of two factors or a combination thereof. The first explanation is that this observation reflects genuine evolutionary conservation; the implication is that, once an intron is gained, it is hard to lose, perhaps, due to functional importance of introns or because the deletion event itself is destructive. The second explanation is that different lineages gain introns at the same position, independently. The chance of such parallel gain is not necessarily as low as it seems at first sight because introns are, apparently, preferentially inserted into proto-splice sites (see above). Accordingly, regions of high sequence conservation might gain introns at exactly the same position.

The two explanations have opposing impacts on our understanding of the evolution of eukaryotic genes. If introns are persistent, many must have been gained early in the eukaryotic evolution, and the later evolution involved multiple losses [16,38]. By contrast, if parallel gain is the dominant mechanism, many of the extant introns are evolutionarily young, and the eukaryotic evolution involves, primarily, multiple introns gains [12].

In order to estimate the extent of parallel gain, we examined all the sites that host introns in at least two species. Assuming that these sites are not invariant, the probability was estimated that the corresponding pattern had arisen from parallel gain, by computing the probability of the last common ancestor of the intron-bearing species to lack an intron. This approach assumes that, if the last common ancestor had an intron in a particular position, no parallel gain occurred, i.e., highly unlikely scenarios involving at least two gains and one loss at the same site are neglected. The probabilities of parallel gain for all the patterns observed in our data set are summarized in Additional file 3. Overall, the data set harbors 3176 sites with shared intron positions (741 unique patterns) out of which 317 positions (10%) are expected to result from parallel intron gain. As inferences involving the root of the tree are prone to significantly elevated standard errors [20], the calculation was repeated using only patterns that do not have the root as the last common ancestor of all intron-bearing species. This calculation yielded 2913 sites with shared intron positions (568 unique patterns) of which 229 (~7.9%) are expected to be due to parallel intron gain. Each of these calculations provides a rough estimate of the level of errors introduced into some of the recent studies that explicitly excluded the possibility of parallel gain [13,16,21].

Importantly, however, the contributions of parallel gains to the emergence of different patterns of intron sharing are widely different; in particular, some rare patterns are explained (almost) entirely by parallel gain and do not reflect evolutionary conservation (Additional file 3). For example, for the single site that harbors introns only in humans and *N. crassa*, the probability is >0.99 that it results from parallel gain. Considering somewhat more frequent patterns, 11 sites harbor introns in *C. intestinalis*, *A. thaliana *and *O. saliva*, with the probability of parallel gain ~0.875, and another 12 sites harbor introns only in *C. elegans *and *C. intestinalis*, with the probability of parallel gain ~0.8.

Generally, the distribution of parallel gains in comparisons of specific clades is, obviously, more informative than overall counting (Table 1). To obtain this information, for each internal node *t *(excluding the root of the tree), all patterns that have 1s in the two sub-clades stemming from *t*, VtL
 MathType@MTEF@5@5@+=feaafiart1ev1aaatCvAUfKttLearuWrP9MDH5MBPbIqV92AaeXatLxBI9gBaebbnrfifHhDYfgasaacH8akY=wiFfYdH8Gipec8Eeeu0xXdbba9frFj0=OqFfea0dXdd9vqai=hGuQ8kuc9pgc9s8qqaq=dirpe0xb9q8qiLsFr0=vr0=vr0dc8meaabaqaciaacaGaaeqabaqabeGadaaakeaacqWGwbGvdaqhaaWcbaGaemiDaqhabaGaemitaWeaaaaa@30A0@ and VtR
 MathType@MTEF@5@5@+=feaafiart1ev1aaatCvAUfKttLearuWrP9MDH5MBPbIqV92AaeXatLxBI9gBaebbnrfifHhDYfgasaacH8akY=wiFfYdH8Gipec8Eeeu0xXdbba9frFj0=OqFfea0dXdd9vqai=hGuQ8kuc9pgc9s8qqaq=dirpe0xb9q8qiLsFr0=vr0=vr0dc8meaabaqaciaacaGaaeqabaqabeGadaaakeaacqWGwbGvdaqhaaWcbaGaemiDaqhabaGaemOuaifaaaaa@30AC@ were tallied, and the probability that *t *is in state zero was computed. By this analysis, in which 728 unique patterns were included, we found that nearly 20% of the shared intron positions between plants and unikonts, thought to have diverged more than a billion and a half years ago [39], are due to parallel gain. In fungi and metazoa, diverged ~1.4 billion years ago, >10% of the shared positions are estimated to derive from parallel gains. In contrast, many recently diverged clades show almost no parallel gain (e.g., humans versus rodents, birds versus mammals, *Aspergillus *versus *Neurospora*, and flies versus mosquitoes). Table 2 lists all patterns for which the estimated contribution to parallel gain was greater than two sites. The estimated total number of sites with parallel gain is 248. Out of the 728 unique patterns, the 22 in this list (3%) account for 116 parallel gains, i.e., ~47% of the total estimated number.

**Table 1:** The estimated number of parallel gains on the branches stemming out of each of the internal nodes in the phylogenetic tree (excluding the root; see Additional file 4)

**internal node**	**Subclade_1**	**subclade_2**	**total number of shared sites**	**total number of parallel gains [95% confidence inrterval]**	**% parallel gains [95% confidence inrterval]**
AME	Unikonts	Magnoliophyta	630	122.8 [38.5 – 229.3]	19.5 [6.1 – 36.4]
Unikonts	Dicdi	Opisthokonts	212	4.9 [1.6 – 15.3]	2.3 [0.7 – 7.2]
Opisthokonts	Metazoa	Fungi	606	70.7 [23.0 – 123.1]	11.7 [3.8 – 20.3]
Metazoa	Caeel	Coelomata	374	24.0 [7.8 – 38.7]	6.4 [2.1 – 10.4]
Coelomata	Deuterostomia	Diptera	350	7.0 [2.4 – 11.5]	2.0 [0.7 – 3.3]
Deuterostomia	Strpu	Chordata	1395	4.7 [1.6 – 8.2]	0.3 [0.1 – 0.6]
Diptera	Drome	Anoga	192	0.0 [0.0 – 0.1]	0.0 [0.0-0.0]
Fungi	Cryne	Ascomycota	223	7.0 [2.4 – 13.7]	3.1 [1.1 – 6.1]
Ascomycota	Schpo	ScAfNc	82	0.3 [0.0 – 0.5]	0.3 [0.0 – 0.7]
ScAfNc	Sacce	Pezizomycotina	5	0.0 [0.0 – 0.1]	0.5 [0.1 – 1.2]
Magnoliophyta	Arath	Orysa	1337	0.2 [0.1 – 0.5]	0.0 [0.0-0.0]
Chordata	Cioin	Vertebrata	822	2.5 [0.9 – 4.2]	0.3 [0.1 – 0.5]
Vertebrata	Danre	Amniota	1701	0.3 [0.1 – 0.5]	0.0 [0.0-0.0]
Apicomplexa	Thepa	Plafa	113	3.9 [0.4 – 8.0]	3.4 [0.3 – 7.1]
Pezizomycotina	Aspfu	Neucr	221	0.1 [0.0 – 0.3]	0.1 [0.0 – 0.1]
Amniota	Galga	Mammals	1659	0.1 [0.0 – 0.1]	0.0 [0.0-0.0]
Mammals	Homsa	Roden	1448	0.0 [0.0-0.0]	0.0 [0.0-0.0]

Species abbreviations are as in Fig. 1. AME stands for the last common Ancestor of Multicellular Eukaryotes

**Table 2:** Patterns that are estimated to contribute more than two sites to the total count of parallel gains

**Pattern**	**total number of patterns**	**Estimated number of parallel gains**
Caeel, Arath, Orysa	20	14.9
Cryne, Arath, Orysa	28	12.6
Cioin, Arath, Orysa	11	9.6
Caeel, Cioin	12	9.6
Strpu, Danre, Galga, Homsa, Arath, Orysa, Roden	39	7.5
Strpu, Cioin, Danre, Galga, Homsa, Arath, Orysa, Roden	32	6.1
Strpu, Cryne	16	5.8
Strpu, Arath, Orysa	13	5.7
Danre, Galga, Homsa, Arath, Orysa, Roden	13	5.3
Cioin, Cryne	5	4.5
Caeel, Cryne	6	4.3
Strpu, Danre, Galga, Homsa, Cryne, Roden	32	4.1
Thepa, Plafa	65	3.3
Caeel, Thepa	23	3.0
Caeel, Strpu, Cioin, Danre, Galga, Homsa, Arath, Orysa, Roden	4	2.9
Aspfu, Arath	3	2.6
Dicdi, Strpu, Danre, Galga, Homsa, Aspfu, Neucr, Roden	4	2.6
Caeel, Strpu, Danre, Galga, Homsa, Drome, Anoga, Arath, Orysa, Roden	7	2.3
Caeel, Strpu, Danre, Galga, Homsa, Schpo	14	2.3
Arath, Orysa, Thepa, Plafa	3	2.3
Cioin, Danre, Galga, Homsa, Arath, Orysa, Roden	7	2.2
Danre, Galga, Homsa, Drome, Anoga	3	2.0

Species and lineages abbreviations are as in Figure 1.

Given that most of the other studies report overall estimates on a smaller (8 species) data set [13], a direct comparison of their results with the present ones is not feasible due to the apparent non-uniformity in the extent of parallel gain in different parts of the tree. Nevertheless, an overall parallel gain of ~10% has been computed also for the smaller data set. Rogozin *et al*. [13] used simulations to assess the extent of parallel gain and showed that, under various assumptions regarding the density of proto-splice sites, parallel gains could be responsible to ~2–40% of the shared intron positions. Accounting more accurately for the likely density of proto-splice sites, Sverdlov et al. estimated the contribution of parallel gains to the observed sharing of intron positions to be in the range of 5–10% [23]. An even higher estimate, 18.5% parallel gains, was obtained by Nguyen et al. using a branch-specific maximum-likelihood model [18].

### Intron retention

We conclude, therefore, that a substantial majority of the shared intron positions are due to evolutionary conservation, hence intron positions tend to be retained for long times. To further validate this conclusion, we explicitly computed the probability of an intron to survive along given paths in the phylogenetic tree. To this end, let *B *denote the set of branches that comprise a path in the tree. Then, intron retention probability along this path is

PB=∑k'=1Kθfk'θ∏t∈B(1−φt)e−θk'gΔt,
 MathType@MTEF@5@5@+=feaafiart1ev1aaatCvAUfKttLearuWrP9MDH5MBPbIqV92AaeXatLxBI9gBaebbnrfifHhDYfgasaacH8akY=wiFfYdH8Gipec8Eeeu0xXdbba9frFj0=OqFfea0dXdd9vqai=hGuQ8kuc9pgc9s8qqaq=dirpe0xb9q8qiLsFr0=vr0=vr0dc8meaabaqaciaacaGaaeqabaqabeGadaaakeaacqWGqbaudaWgaaWcbaGaemOqaieabeaakiabg2da9maaqahabaGaemOzay2aa0baaSqaaiabdUgaRjabcEcaNaqaaGGaciab=H7aXbaakmaarafabaGaeiikaGIaeGymaeJaeyOeI0Iae8NXdy2aaSbaaSqaaiabdsha0bqabaGccqGGPaqkcqWGLbqzdaahaaWcbeqaaiabgkHiTiab=H7aXnaaBaaameaacqWGRbWAcqGGNaWjcqWGNbWzaeqaaSGaeuiLdq0aaSbaaWqaaiabdsha0bqabaaaaaWcbaGaemiDaqNaeyicI4SaemOqaieabeqdcqGHpis1aaWcbaGaem4AaSMaei4jaCIaeyypa0JaeGymaedabaGaem4saS0aaSbaaWqaaiab=H7aXbqabaaaniabggHiLdGccqGGSaalaaa@57D4@

where (1−φt)e−θk'gΔt
 MathType@MTEF@5@5@+=feaafiart1ev1aaatCvAUfKttLearuWrP9MDH5MBPbIqV92AaeXatLxBI9gBaebbnrfifHhDYfgasaacH8akY=wiFfYdH8Gipec8Eeeu0xXdbba9frFj0=OqFfea0dXdd9vqai=hGuQ8kuc9pgc9s8qqaq=dirpe0xb9q8qiLsFr0=vr0=vr0dc8meaabaqaciaacaGaaeqabaqabeGadaaakeaacqGGOaakcqaIXaqmcqGHsisliiGacqWFgpGzdaWgaaWcbaGaemiDaqhabeaakiabcMcaPiabdwgaLnaaCaaaleqabaGaeyOeI0Iae8hUde3aaSbaaWqaaiabdUgaRjabcEcaNiabdEgaNbqabaWccqqHuoardaWgaaadbaGaemiDaqhabeaaaaaaaa@3E88@ is the retention probability along branch *t*, and fk'θ
 MathType@MTEF@5@5@+=feaafiart1ev1aaatCvAUfKttLearuWrP9MDH5MBPbIqV92AaeXatLxBI9gBaebbnrfifHhDYfgasaacH8akY=wiFfYdH8Gipec8Eeeu0xXdbba9frFj0=OqFfea0dXdd9vqai=hGuQ8kuc9pgc9s8qqaq=dirpe0xb9q8qiLsFr0=vr0=vr0dc8meaabaqaciaacaGaaeqabaqabeGadaaakeaacqWGMbGzdaqhaaWcbaGaem4AaSMaei4jaCcabaacciGae8hUdehaaaaa@3220@ is the probability of being at loss-rate category *k'*.

In accord with the above conclusions on the high level of evolutionary conservation, this probability is, typically, high, even for very long paths in the phylogenetic tree (Table 3). For example, an intron present in the last common ancestor of the metazoa has a probability of 0.83 to be retained in humans whereas an intron present in the last common ancestor of multicellular life (AME) has a probability of 0.57 to be retained in extant plants.

**Table 3:** Probabilities of an intron to be retained along selected paths in the phylogenetic tree

If an intron was present in	chances are	that it would be present in	95% confidence interval
AME	0.63	Homsa	[0.57 – 0.68]
Metazoa	0.83	Homsa	[0.79 – 0.84]
Deuterostomia	0.86	Homsa	[0.85 – 0.88]
Vertebrata	0.95	Homsa	[0.94 – 0.96]
Mammals	0.96	Homsa	[0.95 – 0.97]
Fungi	0.01	Sacce	[0.00 – 0.01]
Fungi	0.26	Aspfu	[0.24 – 0.27]
Apicomplexa	0.26	Plafa	[0.20 – 0.33]
Apicomplexa	0.69	Thepa	[0.57 – 0.80]
AME	0.57	Arath	[0.50 – 0.64]
AME	0.57	Orysa	[0.50 – 0.65]

Species and lineage abbreviations are as in Fig. 1.

**Figure 2:**
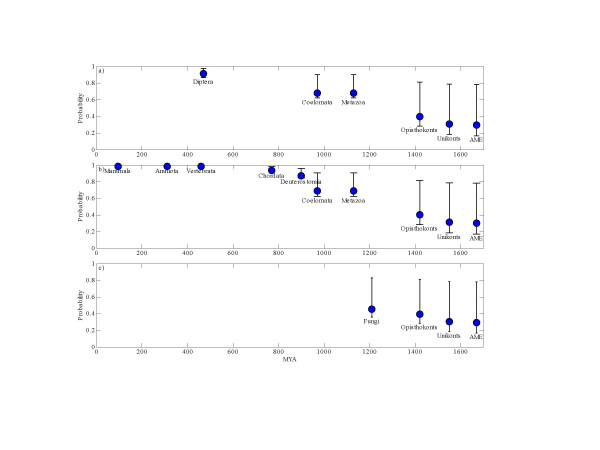
The probability of an intron in extant species to be present in ancient ancestors. **a) **an intron in *D. melanogaster*, **b) **an intron in *H. sapiens*, **c) **an intron in *C. neoformans*. AME stands for the last common ancestor of multicellular eukaryotes.

A complementary approach involves the computation of the probability of extant introns to be of ancient origin. To calculate this probability, we assumed that a site is known to host an intron in one species, and that no information is available on this site in other species (that is, their state is *). Then, we used our model to compute the probability that this intron was present in any of the ancestors of that species (Table 4 and Fig. 2). Clearly, these probabilities decay quite slowly with evolutionary time. For instance, an intron in *C. elegans*, *H. sapiens *and *D. melanogaster *has a probability of 0.44, 0.69, and 0.68, respectively, to have been present in the last common ancestor of metazoa.

**Table 4:** Probability of an intron in extant species to be inherited from an ancestral node

intron is present in	Probability	that it is also present in	95% confidence interval
Dicdi	0.71	AME	[0.46 – 0.92]
Caeel	0.19	AME	[0.10 – 0.74]
Strpu	0.31	AME	[0.17 – 0.79]
Cioin	0.22	AME	[0.12 – 0.75]
Danre	0.29	AME	[0.16 – 0.78]
Galga	0.29	AME	[0.16 – 0.78]
Homsa	0.30	AME	[0.17 – 0.78]
Roden	0.30	AME	[0.17 – 0.78]
Drome	0.30	AME	[0.17 – 0.78]
Anoga	0.28	AME	[0.16 – 0.78]
Cryne	0.29	AME	[0.17 – 0.78]
Schpo	0.39	AME	[0.24 – 0.82]
Sacce	0.27	AME	[0.16 – 0.77]
Aspfu	0.27	AME	[0.15 – 0.77]
Neucr	0.21	AME	[0.11 – 0.75]
Arath	0.31	AME	[0.18 – 0.79]
Orysa	0.30	AME	[0.17 – 0.78]
Thepa	0.30	Apicomplexa	[0.10 – 0.77]
Plafa	0.46	Apicomplexa	[0.21 – 0.81]

Confidence intervals are wide due to the large amount of uncertainty associated with the input patterns (each pattern has 18 out of 19 sites with the presence/absence of the intron marked as unknown). Species and lineages abbreviations are as in Table 1.

## Conclusion

Despite the extensive attention given to the evolution of eukaryotic gene structure over the last three decades, the fundamental characteristics of this process remain controversial. In particular, depending on the methods and data sets used, different researchers have reached opposite conclusions on the causes of the remarkably high fraction of shared introns in orthologous genes from distant eukaryotic species. Some attribute it (almost) entirely to a remarkable evolutionary conservation of intron positions and others, largely, to parallel gain of introns. To resolve these contradictions, it is important to analyze the evolution of introns by using a probabilistic model that minimally relies on arbitrary assumptions. To this end, we developed a model that allows for variability of intron gain and loss rates over branches of the phylogenetic tree, individual genes, and individual sites. Applying this model to an extended set of conserved eukaryotic genes, we found that parallel gain, on average, accounts for only ~8% of the shared intron positions. However, the distribution of parallel gains over the phylogenetic tree of eukaryotes is highly non-uniform such that there are, practically, no parallel gains in closely related lineages, whereas for distant lineages, such as animals and plants, parallel gains might have contributed up to 20% of the shared intron positions. Given the distinctly non-uniform distribution of the inferred gain events over the phylogenetic tree of eukaryotes [20], most of the recently diverged lineages have amassed very few gains during their separate evolution; by contrast, deeply diverged lineages, such as animals and plants appear to have gone through independent stages of extensive intron gain, which would explain the greater share of parallel gains.

We estimated that ancestral introns have a high probability to be retained in extant genomes, and conversely, many of the extant introns are ancient. The reasons for this remarkable endurance of a substantial fraction of the introns are not clear. One possibility is mechanistic, i.e., removing existing introns might be imprecise and hence potentially deleterious. Another possibility is functional, i.e., introns might have acquired many functional roles since entering eukaryotic genomes. The latter possibility is compatible with the recent observation of a negative correlation between the rate of intron gain and coding sequence evolution rate of a gene [19] which suggests that at least some of the introns are functionally relevant.

## Methods

### The data set

The methods and criteria used in compiling the data set have been described previously [19,20]. Briefly, the analyzed data set consisted of the reliable alignments of 391 genes from 19 eukaryotic species (a total of 289,902 sites). These include 9 animals (*Caenorhabditis elegans*, *Strongylocentrotus purpuratus*, *Ciona intestinalis*, *Danio rerio*, *Gallus gallus*, *Homo sapiens*, rodents (*Mus musculus *and *Rattus norvegicus *combined), *Drosophila melanogaster*, *Anopheles gambiae*); 5 fungi (*Cryptococcus neoformans*, *Schizosaccharomyces pombe*, *Saccharomyces cerevisiae*, *Aspergillus fumigatus*, *Neurospora crassa*); two plants (*Arabidopsis thaliana*, *Oryza sativa*); two apicomplexans (*Theileria parva*, *Plasmodium falciparum*); and the amoebozoan *Dictyostelium discoideum*.

### Phylogenetic tree topology

Throughout this paper we assumed the traditional "crown-group" tree topology (Additional file 4). Specifically, the root position is between the Apicomplexa and the common ancestor of multicellular eukaryotes (plants and animals) [40], as opposed to the alternative Unikont-Bikont division [41]. We furthermore assume the Coelomata topology (Deuterostomia and insects are grouped together to the exclusion of nematodes) [42,43] as opposed to the Ecdysozoa topology (insects and nematodes are grouped together to the exclusion of Deuterostomia) [44,45]. The results, however, are not sensitive to the exact tree topology, as explicitly shown elsewhere [19,20].

### The EM Algorithm

For each site, the *S *leaves form a set of observed random variables, their states being described by the corresponding pattern *ω*_*p*_. The states of all the internal nodes, denoted *σ*, form a set of hidden random variables, that is, random variables whose states are not observed. In order to account for rate variability across sites, we associate with each pattern two hidden random variables, ρpη
 MathType@MTEF@5@5@+=feaafiart1ev1aaatCvAUfKttLearuWrP9MDH5MBPbIqV92AaeXatLxBI9gBaebbnrfifHhDYfgasaacH8akY=wiFfYdH8Gipec8Eeeu0xXdbba9frFj0=OqFfea0dXdd9vqai=hGuQ8kuc9pgc9s8qqaq=dirpe0xb9q8qiLsFr0=vr0=vr0dc8meaabaqaciaacaGaaeqabaqabeGadaaakeaaiiGacqWFbpGCdaqhaaWcbaGaemiCaahabaGae83TdGgaaaaa@31B0@ and ρpθ
 MathType@MTEF@5@5@+=feaafiart1ev1aaatCvAUfKttLearuWrP9MDH5MBPbIqV92AaeXatLxBI9gBaebbnrfifHhDYfgasaacH8akY=wiFfYdH8Gipec8Eeeu0xXdbba9frFj0=OqFfea0dXdd9vqai=hGuQ8kuc9pgc9s8qqaq=dirpe0xb9q8qiLsFr0=vr0=vr0dc8meaabaqaciaacaGaaeqabaqabeGadaaakeaaiiGacqWFbpGCdaqhaaWcbaGaemiCaahabaGae8hUdehaaaaa@31BA@, that determine the value of the rate variables in that site. To sum up, the observed random variables are *ω*_*p*_, and the hidden random variables are (*σ*, ρpη
 MathType@MTEF@5@5@+=feaafiart1ev1aaatCvAUfKttLearuWrP9MDH5MBPbIqV92AaeXatLxBI9gBaebbnrfifHhDYfgasaacH8akY=wiFfYdH8Gipec8Eeeu0xXdbba9frFj0=OqFfea0dXdd9vqai=hGuQ8kuc9pgc9s8qqaq=dirpe0xb9q8qiLsFr0=vr0=vr0dc8meaabaqaciaacaGaaeqabaqabeGadaaakeaaiiGacqWFbpGCdaqhaaWcbaGaemiCaahabaGae83TdGgaaaaa@31B0@, ρpθ
 MathType@MTEF@5@5@+=feaafiart1ev1aaatCvAUfKttLearuWrP9MDH5MBPbIqV92AaeXatLxBI9gBaebbnrfifHhDYfgasaacH8akY=wiFfYdH8Gipec8Eeeu0xXdbba9frFj0=OqFfea0dXdd9vqai=hGuQ8kuc9pgc9s8qqaq=dirpe0xb9q8qiLsFr0=vr0=vr0dc8meaabaqaciaacaGaaeqabaqabeGadaaakeaaiiGacqWFbpGCdaqhaaWcbaGaemiCaahabaGae8hUdehaaaaa@31BA@).

We assume that sites within a gene, as well as the genes themselves, evolve independently. Therefore, the total likelihood can be decomposed as

L(M1,…,MG|Θ)=∏g=1GL(Mg|Ξ,Ψg,Λ)=∏g=1G∏p=1ΩL(ωp|Ξ,Ψg,Λ)ngp.
 MathType@MTEF@5@5@+=feaafiart1ev1aaatCvAUfKttLearuWrP9MDH5MBPbIqV92AaeXatLxBI9gBaebbnrfifHhDYfgasaacH8akY=wiFfYdH8Gipec8Eeeu0xXdbba9frFj0=OqFfea0dXdd9vqai=hGuQ8kuc9pgc9s8qqaq=dirpe0xb9q8qiLsFr0=vr0=vr0dc8meaabaqaciaacaGaaeqabaqabeGadaaakeaacqWGmbatcqGGOaakcqWGnbqtdaWgaaWcbaGaeGymaedabeaakiabcYcaSiablAciljabcYcaSiabd2eannaaBaaaleaacqWGhbWraeqaaOGaeiiFaWNaeuiMdeLaeiykaKIaeyypa0ZaaebCaeaacqWGmbatcqGGOaakcqWGnbqtdaWgaaWcbaGaem4zaCgabeaakiabcYha8jabf65ayjabcYcaSiabfI6aznaaBaaaleaacqWGNbWzaeqaaOGaeiilaWIaeu4MdWKaeiykaKcaleaacqWGNbWzcqGH9aqpcqaIXaqmaeaacqWGhbWra0Gaey4dIunakiabg2da9maarahabaWaaebCaeaacqWGmbatcqGGOaakiiGacqWFjpWDdaWgaaWcbaGaemiCaahabeaakiabcYha8jabf65ayjabcYcaSiabfI6aznaaBaaaleaacqWGNbWzaeqaaOGaeiilaWIaeu4MdWKaeiykaKYaaWbaaSqabeaacqWGUbGBdaWgaaadbaGaem4zaCMaemiCaahabeaaaaGccqGGUaGlaSqaaiabdchaWjabg2da9iabigdaXaqaaiabfM6axbqdcqGHpis1aaWcbaGaem4zaCMaeyypa0JaeGymaedabaGaem4raCeaniabg+Givdaaaa@744F@

and so

log⁡L(M1,…,MG|Θ)=∑g=1G∑p=1Ωngplog⁡L(ωp|Ξ,Ψg,Λ).
 MathType@MTEF@5@5@+=feaafiart1ev1aaatCvAUfKttLearuWrP9MDH5MBPbIqV92AaeXatLxBI9gBaebbnrfifHhDYfgasaacH8akY=wiFfYdH8Gipec8Eeeu0xXdbba9frFj0=OqFfea0dXdd9vqai=hGuQ8kuc9pgc9s8qqaq=dirpe0xb9q8qiLsFr0=vr0=vr0dc8meaabaqaciaacaGaaeqabaqabeGadaaakeaacyGGSbaBcqGGVbWBcqGGNbWzcqWGmbatcqGGOaakcqWGnbqtdaWgaaWcbaGaeGymaedabeaakiabcYcaSiablAciljabcYcaSiabd2eannaaBaaaleaacqWGhbWraeqaaOGaeiiFaWNaeuiMdeLaeiykaKIaeyypa0ZaaabCaeaadaaeWbqaaiabd6gaUnaaBaaaleaacqWGNbWzcqWGWbaCaeqaaOGagiiBaWMaei4Ba8Maei4zaCMaemitaWKaeiikaGccciGae8xYdC3aaSbaaSqaaiabdchaWbqabaGccqGG8baFcqqHEoawcqGGSaalcqqHOoqwdaWgaaWcbaGaem4zaCgabeaakiabcYcaSiabfU5amjabcMcaPaWcbaGaemiCaaNaeyypa0JaeGymaedabaGaeuyQdCfaniabggHiLdaaleaacqWGNbWzcqGH9aqpcqaIXaqmaeaacqWGhbWra0GaeyyeIuoakiabc6caUaaa@660C@

According to the well-known EM paradigm [35], log *L*(*M*_1_, ..., *M*_*G*_|Θ) is guaranteed to increase as long as we maximize the auxiliary function

Q(Θ,Θ0)=∑g=1G∑p=1ΩngpQgp(Ξ,Ψg,Λ,Ξ0,Ψg0,Λ0),
 MathType@MTEF@5@5@+=feaafiart1ev1aaatCvAUfKttLearuWrP9MDH5MBPbIqV92AaeXatLxBI9gBaebbnrfifHhDYfgasaacH8akY=wiFfYdH8Gipec8Eeeu0xXdbba9frFj0=OqFfea0dXdd9vqai=hGuQ8kuc9pgc9s8qqaq=dirpe0xb9q8qiLsFr0=vr0=vr0dc8meaabaqaciaacaGaaeqabaqabeGadaaakeaacqWGrbqucqGGOaakcqqHyoqucqGGSaalcqqHyoqudaahaaWcbeqaaiabicdaWaaakiabcMcaPiabg2da9maaqahabaWaaabCaeaacqWGUbGBdaWgaaWcbaGaem4zaCMaemiCaahabeaakiabdgfarnaaBaaaleaacqWGNbWzcqWGWbaCaeqaaOGaeiikaGIaeuONdGLaeiilaWIaeuiQdK1aaSbaaSqaaiabdEgaNbqabaGccqGGSaalcqqHBoatcqGGSaalcqqHEoawdaahaaWcbeqaaiabicdaWaaakiabcYcaSiabfI6aznaaDaaaleaacqWGNbWzaeaacqaIWaamaaGccqGGSaalcqqHBoatdaahaaWcbeqaaiabicdaWaaakiabcMcaPaWcbaGaemiCaaNaeyypa0JaeGymaedabaGaeuyQdCfaniabggHiLdaaleaacqWGNbWzcqGH9aqpcqaIXaqmaeaacqWGhbWra0GaeyyeIuoakiabcYcaSaaa@6232@

where

Qgp(Ξ,Ψg,Λ,Ξ0,Ψg0,Λ0)=∑σ,ρpη,ρpθPr⁡(σ,ρpη,ρpθ|ωp,Ξ0,Ψg0,Λ0)log⁡Pr⁡(ωp,σ,ρpη,ρpθ|Ξ,Ψg,Λ).
 MathType@MTEF@5@5@+=feaafiart1ev1aaatCvAUfKttLearuWrP9MDH5MBPbIqV92AaeXatLxBI9gBaebbnrfifHhDYfgasaacH8akY=wiFfYdH8Gipec8Eeeu0xXdbba9frFj0=OqFfea0dXdd9vqai=hGuQ8kuc9pgc9s8qqaq=dirpe0xb9q8qiLsFr0=vr0=vr0dc8meaabaqaciaacaGaaeqabaqabeGadaaakqaabeqaaiabdgfarnaaBaaaleaacqWGNbWzcqWGWbaCaeqaaOGaeiikaGIaeuONdGLaeiilaWIaeuiQdK1aaSbaaSqaaiabdEgaNbqabaGccqGGSaalcqqHBoatcqGGSaalcqqHEoawdaahaaWcbeqaaiabicdaWaaakiabcYcaSiabfI6aznaaDaaaleaacqWGNbWzaeaacqaIWaamaaGccqGGSaalcqqHBoatdaahaaWcbeqaaiabicdaWaaakiabcMcaPiabg2da9aqaaiaaxMaadaaeqbqaaiGbccfaqjabckhaYjabcIcaOGGaciab=n8aZjabcYcaSiab=f8aYnaaDaaaleaacqWGWbaCaeaacqWF3oaAaaGccqGGSaalcqWFbpGCdaqhaaWcbaGaemiCaahabaGae8hUdehaaOGaeiiFaWNae8xYdC3aaSbaaSqaaiabdchaWbqabaGccqGGSaalcqqHEoawdaahaaWcbeqaaiabicdaWaaakiabcYcaSiabfI6aznaaDaaaleaacqWGNbWzaeaacqaIWaamaaGccqGGSaalcqqHBoatdaahaaWcbeqaaiabicdaWaaakiabcMcaPiGbcYgaSjabc+gaVjabcEgaNjGbccfaqjabckhaYjabcIcaOiab=L8a3naaBaaaleaacqWGWbaCaeqaaOGaeiilaWIae83WdmNaeiilaWIae8xWdi3aa0baaSqaaiabdchaWbqaaiab=D7aObaakiabcYcaSiab=f8aYnaaDaaaleaacqWGWbaCaeaacqWF4oqCaaGccqGG8baFcqqHEoawcqGGSaalcqqHOoqwdaWgaaWcbaGaem4zaCgabeaakiabcYcaSiabfU5amjabcMcaPaWcbaGae83WdmNaeiilaWIae8xWdi3aa0baaWqaaiabdchaWbqaaiab=D7aObaaliabcYcaSiab=f8aYnaaDaaameaacqWGWbaCaeaacqWF4oqCaaaaleqaniabggHiLdGccqGGUaGlaaaa@9ED5@

If we replace the formal summing over all states of ρpη
 MathType@MTEF@5@5@+=feaafiart1ev1aaatCvAUfKttLearuWrP9MDH5MBPbIqV92AaeXatLxBI9gBaebbnrfifHhDYfgasaacH8akY=wiFfYdH8Gipec8Eeeu0xXdbba9frFj0=OqFfea0dXdd9vqai=hGuQ8kuc9pgc9s8qqaq=dirpe0xb9q8qiLsFr0=vr0=vr0dc8meaabaqaciaacaGaaeqabaqabeGadaaakeaaiiGacqWFbpGCdaqhaaWcbaGaemiCaahabaGae83TdGgaaaaa@31B0@ and ρpθ
 MathType@MTEF@5@5@+=feaafiart1ev1aaatCvAUfKttLearuWrP9MDH5MBPbIqV92AaeXatLxBI9gBaebbnrfifHhDYfgasaacH8akY=wiFfYdH8Gipec8Eeeu0xXdbba9frFj0=OqFfea0dXdd9vqai=hGuQ8kuc9pgc9s8qqaq=dirpe0xb9q8qiLsFr0=vr0=vr0dc8meaabaqaciaacaGaaeqabaqabeGadaaakeaaiiGacqWFbpGCdaqhaaWcbaGaemiCaahabaGae8hUdehaaaaa@31BA@ in (6) by a direct sum, we get

Qgp(Ξ,Ψg,Λ,Ξ0,Ψg0,Λ0)=∑k=1Kη∑k'=1Kθ∑σPr⁡(σ,ρpη=k,ρpθ=k'|ωp,Ξ0,Ψg0,Λ0)log⁡Pr⁡(ωp,σ,ρpη=k,ρpθ=k'|Ξ,Ψg,Λ).
 MathType@MTEF@5@5@+=feaafiart1ev1aaatCvAUfKttLearuWrP9MDH5MBPbIqV92AaeXatLxBI9gBaebbnrfifHhDYfgasaacH8akY=wiFfYdH8Gipec8Eeeu0xXdbba9frFj0=OqFfea0dXdd9vqai=hGuQ8kuc9pgc9s8qqaq=dirpe0xb9q8qiLsFr0=vr0=vr0dc8meaabaqaciaacaGaaeqabaqabeGadaaakqaabeqaaiabdgfarnaaBaaaleaacqWGNbWzcqWGWbaCaeqaaOGaeiikaGIaeuONdGLaeiilaWIaeuiQdK1aaSbaaSqaaiabdEgaNbqabaGccqGGSaalcqqHBoatcqGGSaalcqqHEoawdaahaaWcbeqaaiabicdaWaaakiabcYcaSiabfI6aznaaDaaaleaacqWGNbWzaeaacqaIWaamaaGccqGGSaalcqqHBoatdaahaaWcbeqaaiabicdaWaaakiabcMcaPiabg2da9aqaaiaaxMaadaaeWbqaamaaqahabaWaaabuaeaacyGGqbaucqGGYbGCcqGGOaakiiGacqWFdpWCcqGGSaalcqWFbpGCdaqhaaWcbaGaemiCaahabaGae83TdGgaaOGaeyypa0Jaem4AaSMaeiilaWIae8xWdi3aa0baaSqaaiabdchaWbqaaiab=H7aXbaakiabg2da9iabdUgaRjabcEcaNiabcYha8jab=L8a3naaBaaaleaacqWGWbaCaeqaaOGaeiilaWIaeuONdG1aaWbaaSqabeaacqaIWaamaaGccqGGSaalcqqHOoqwdaqhaaWcbaGaem4zaCgabaGaeGimaadaaOGaeiilaWIaeu4MdW0aaWbaaSqabeaacqaIWaamaaGccqGGPaqkcyGGSbaBcqGGVbWBcqGGNbWzcyGGqbaucqGGYbGCcqGGOaakcqWFjpWDdaWgaaWcbaGaemiCaahabeaakiabcYcaSiab=n8aZjabcYcaSiab=f8aYnaaDaaaleaacqWGWbaCaeaacqWF3oaAaaGccqGH9aqpcqWGRbWAcqGGSaalcqWFbpGCdaqhaaWcbaGaemiCaahabaGae8hUdehaaOGaeyypa0Jaem4AaSMaei4jaCIaeiiFaWNaeuONdGLaeiilaWIaeuiQdK1aaSbaaSqaaiabdEgaNbqabaGccqGGSaalcqqHBoatcqGGPaqkaSqaaiab=n8aZbqab0GaeyyeIuoaaSqaaiabdUgaRjabcEcaNiabg2da9iabigdaXaqaaiabdUealnaaBaaameaacqWF4oqCaeqaaaqdcqGHris5aaWcbaGaem4AaSMaeyypa0JaeGymaedabaGaem4saS0aaSbaaWqaaiab=D7aObqabaaaniabggHiLdGccqGGUaGlaaaa@B037@

Using our notational conventions, we can write the first term in (7) as

Pr⁡(σ,ρpη=k,ρpθ=k'|ωp,Ξ0,Ψg0,Λ0)=Pr⁡(ρpη=k,ρpθ=k'|ωp,Ξ0,Ψg0,Λ0)⋅Pr⁡(σ|ωp,Ξ0,Ψgkk'0),
 MathType@MTEF@5@5@+=feaafiart1ev1aaatCvAUfKttLearuWrP9MDH5MBPbIqV92AaeXatLxBI9gBaebbnrfifHhDYfgasaacH8akY=wiFfYdH8Gipec8Eeeu0xXdbba9frFj0=OqFfea0dXdd9vqai=hGuQ8kuc9pgc9s8qqaq=dirpe0xb9q8qiLsFr0=vr0=vr0dc8meaabaqaciaacaGaaeqabaqabeGadaaakqaabeqaaiGbccfaqjabckhaYjabcIcaOGGaciab=n8aZjabcYcaSiab=f8aYnaaDaaaleaacqWGWbaCaeaacqWF3oaAaaGccqGH9aqpcqWGRbWAcqGGSaalcqWFbpGCdaqhaaWcbaGaemiCaahabaGae8hUdehaaOGaeyypa0Jaem4AaSMaei4jaCIaeiiFaWNae8xYdC3aaSbaaSqaaiabdchaWbqabaGccqGGSaalcqqHEoawdaahaaWcbeqaaiabicdaWaaakiabcYcaSiabfI6aznaaDaaaleaacqWGNbWzaeaacqaIWaamaaGccqGGSaalcqqHBoatdaahaaWcbeqaaiabicdaWaaakiabcMcaPiabg2da9aqaaiaaxMaacyGGqbaucqGGYbGCcqGGOaakcqWFbpGCdaqhaaWcbaGaemiCaahabaGae83TdGgaaOGaeyypa0Jaem4AaSMaeiilaWIae8xWdi3aa0baaSqaaiabdchaWbqaaiab=H7aXbaakiabg2da9iabdUgaRjabcEcaNiabcYha8jab=L8a3naaBaaaleaacqWGWbaCaeqaaOGaeiilaWIaeuONdG1aaWbaaSqabeaacqaIWaamaaGccqGGSaalcqqHOoqwdaqhaaWcbaGaem4zaCgabaGaeGimaadaaOGaeiilaWIaeu4MdW0aaWbaaSqabeaacqaIWaamaaGccqGGPaqkcqGHflY1cyGGqbaucqGGYbGCcqGGOaakcqWFdpWCcqGG8baFcqWFjpWDdaWgaaWcbaGaemiCaahabeaakiabcYcaSiabf65aynaaCaaaleqabaGaeGimaadaaOGaeiilaWIaeuiQdK1aa0baaSqaaiabdEgaNjabdUgaRjabdUgaRjabcEcaNaqaaiabicdaWaaakiabcMcaPiabcYcaSaaaaa@967A@

and the second term as

log⁡Pr⁡(ωp,σ,ρpη=k,ρpθ=k'|Ξ,Ψg,Λ)=log⁡Pr⁡(ρpη=k|Ξ,Ψg,Λ)++log⁡Pr⁡(ρpθ=k'|Ξ,Ψg,Λ)+log⁡Pr⁡(ωp,σ|Ξ,Ψgkk')=log⁡fkη+log⁡fk'θ+log⁡Pr⁡(ωp,σ|Ξ,Ψgkk').
 MathType@MTEF@5@5@+=feaafiart1ev1aaatCvAUfKttLearuWrP9MDH5MBPbIqV92AaeXatLxBI9gBaebbnrfifHhDYfgasaacH8akY=wiFfYdH8Gipec8Eeeu0xXdbba9frFj0=OqFfea0dXdd9vqai=hGuQ8kuc9pgc9s8qqaq=dirpe0xb9q8qiLsFr0=vr0=vr0dc8meaabaqaciaacaGaaeqabaqabeGadaaakqaabeqaaiGbcYgaSjabc+gaVjabcEgaNjGbccfaqjabckhaYjabcIcaOGGaciab=L8a3naaBaaaleaacqWGWbaCaeqaaOGaeiilaWIae83WdmNaeiilaWIae8xWdi3aa0baaSqaaiabdchaWbqaaiab=D7aObaakiabg2da9iabdUgaRjabcYcaSiab=f8aYnaaDaaaleaacqWGWbaCaeaacqWF4oqCaaGccqGH9aqpcqWGRbWAcqGGNaWjcqGG8baFcqqHEoawcqGGSaalcqqHOoqwdaWgaaWcbaGaem4zaCgabeaakiabcYcaSiabfU5amjabcMcaPiabg2da9iGbcYgaSjabc+gaVjabcEgaNjGbccfaqjabckhaYjabcIcaOiab=f8aYnaaDaaaleaacqWGWbaCaeaacqWF3oaAaaGccqGH9aqpcqWGRbWAcqGG8baFcqqHEoawcqGGSaalcqqHOoqwdaWgaaWcbaGaem4zaCgabeaakiabcYcaSiabfU5amjabcMcaPiabgUcaRaqaaiaaxMaacqGHRaWkcyGGSbaBcqGGVbWBcqGGNbWzcyGGqbaucqGGYbGCcqGGOaakcqWFbpGCdaqhaaWcbaGaemiCaahabaGae8hUdehaaOGaeyypa0Jaem4AaSMaei4jaCIaeiiFaWNaeuONdGLaeiilaWIaeuiQdK1aaSbaaSqaaiabdEgaNbqabaGccqGGSaalcqqHBoatcqGGPaqkcqGHRaWkcyGGSbaBcqGGVbWBcqGGNbWzcyGGqbaucqGGYbGCcqGGOaakcqWFjpWDdaWgaaWcbaGaemiCaahabeaakiabcYcaSiab=n8aZjabcYha8jabf65ayjabcYcaSiabfI6aznaaBaaaleaacqWGNbWzcqWGRbWAcqWGRbWAcqGGNaWjaeqaaOGaeiykaKIaeyypa0dabaGaaCzcaiGbcYgaSjabc+gaVjabcEgaNjabdAgaMnaaDaaaleaacqWGRbWAaeaacqWF3oaAaaGccqGHRaWkcyGGSbaBcqGGVbWBcqGGNbWzcqWGMbGzdaqhaaWcbaGaem4AaSMaei4jaCcabaGae8hUdehaaOGaey4kaSIagiiBaWMaei4Ba8Maei4zaCMagiiuaaLaeiOCaiNaeiikaGIae8xYdC3aaSbaaSqaaiabdchaWbqabaGccqGGSaalcqWFdpWCcqGG8baFcqqHEoawcqGGSaalcqqHOoqwdaWgaaWcbaGaem4zaCMaem4AaSMaem4AaSMaei4jaCcabeaakiabcMcaPiabc6caUaaaaa@D5EE@

Substituting (8) and (9) back into (7) gives

Qgp(Ξ,Ψg,Λ,Ξ0,Ψg0,Λ0)=∑k=1Kη∑k'=1Kθ[Pr⁡(ρpη=k,ρpθ=k'|ωp,Ξ0,Ψg0,Λ0)]⋅     ⋅[∑σPr⁡(σ|ωp,Ξ0,Ψgkk'0)⋅{log⁡fkη+log⁡fk'θ+log⁡Pr⁡(ωp,σ|Ξ,Ψgkk')}]
 MathType@MTEF@5@5@+=feaafiart1ev1aaatCvAUfKttLearuWrP9MDH5MBPbIqV92AaeXatLxBI9gBaebbnrfifHhDYfgasaacH8akY=wiFfYdH8Gipec8Eeeu0xXdbba9frFj0=OqFfea0dXdd9vqai=hGuQ8kuc9pgc9s8qqaq=dirpe0xb9q8qiLsFr0=vr0=vr0dc8meaabaqaciaacaGaaeqabaqabeGadaaakqaabeqaaiabdgfarnaaBaaaleaacqWGNbWzcqWGWbaCaeqaaOGaeiikaGIaeuONdGLaeiilaWIaeuiQdK1aaSbaaSqaaiabdEgaNbqabaGccqGGSaalcqqHBoatcqGGSaalcqqHEoawdaahaaWcbeqaaiabicdaWaaakiabcYcaSiabfI6aznaaDaaaleaacqWGNbWzaeaacqaIWaamaaGccqGGSaalcqqHBoatdaahaaWcbeqaaiabicdaWaaakiabcMcaPiabg2da9aqaaiaaxMaadaaeWbqaamaaqahabaWaamWaaeaacyGGqbaucqGGYbGCcqGGOaakiiGacqWFbpGCdaqhaaWcbaGaemiCaahabaGae83TdGgaaOGaeyypa0Jaem4AaSMaeiilaWIae8xWdi3aa0baaSqaaiabdchaWbqaaiab=H7aXbaakiabg2da9iabdUgaRjabcEcaNiabcYha8jab=L8a3naaBaaaleaacqWGWbaCaeqaaOGaeiilaWIaeuONdG1aaWbaaSqabeaacqaIWaamaaGccqGGSaalcqqHOoqwdaqhaaWcbaGaem4zaCgabaGaeGimaadaaOGaeiilaWIaeu4MdW0aaWbaaSqabeaacqaIWaamaaGccqGGPaqkaiaawUfacaGLDbaacqGHflY1aSqaaiabdUgaRjabcEcaNiabg2da9iabigdaXaqaaiabdUealnaaBaaameaacqWF4oqCaeqaaaqdcqGHris5aaWcbaGaem4AaSMaeyypa0JaeGymaedabaGaem4saS0aaSbaaWqaaiab=D7aObqabaaaniabggHiLdaakeaacaWLjaGaaCzcaiabgwSixpaadmaabaWaaabuaeaacyGGqbaucqGGYbGCcqGGOaakcqWFdpWCcqGG8baFcqWFjpWDdaWgaaWcbaGaemiCaahabeaakiabcYcaSiabf65aynaaCaaaleqabaGaeGimaadaaOGaeiilaWIaeuiQdK1aa0baaSqaaiabdEgaNjabdUgaRjabdUgaRjabcEcaNaqaaiabicdaWaaakiabcMcaPiabgwSixpaacmaabaGagiiBaWMaei4Ba8Maei4zaCMaemOzay2aa0baaSqaaiabdUgaRbqaaiab=D7aObaakiabgUcaRiGbcYgaSjabc+gaVjabcEgaNjabdAgaMnaaDaaaleaacqWGRbWAcqGGNaWjaeaacqWF4oqCaaGccqGHRaWkcyGGSbaBcqGGVbWBcqGGNbWzcyGGqbaucqGGYbGCcqGGOaakcqWFjpWDdaWgaaWcbaGaemiCaahabeaakiabcYcaSiab=n8aZjabcYha8jabf65ayjabcYcaSiabfI6aznaaBaaaleaacqWGNbWzcqWGRbWAcqWGRbWAcqGGNaWjaeqaaOGaeiykaKcacaGL7bGaayzFaaaaleaacqWFdpWCaeqaniabggHiLdaakiaawUfacaGLDbaaaaaa@D5C1@

Denoting by *w*_*gpkk*' _and *Q*_*gpkk*' _the first and second square brackets, respectively, this expression becomes

Qgp(Ξ,Ψg,Λ,Ξ0,Ψg0,Λ0)=∑k=1Kη∑k'=1Kθwgpkk'Qgpkk',
 MathType@MTEF@5@5@+=feaafiart1ev1aaatCvAUfKttLearuWrP9MDH5MBPbIqV92AaeXatLxBI9gBaebbnrfifHhDYfgasaacH8akY=wiFfYdH8Gipec8Eeeu0xXdbba9frFj0=OqFfea0dXdd9vqai=hGuQ8kuc9pgc9s8qqaq=dirpe0xb9q8qiLsFr0=vr0=vr0dc8meaabaqaciaacaGaaeqabaqabeGadaaakeaacqWGrbqudaWgaaWcbaGaem4zaCMaemiCaahabeaakiabcIcaOiabf65ayjabcYcaSiabfI6aznaaBaaaleaacqWGNbWzaeqaaOGaeiilaWIaeu4MdWKaeiilaWIaeuONdG1aaWbaaSqabeaacqaIWaamaaGccqGGSaalcqqHOoqwdaqhaaWcbaGaem4zaCgabaGaeGimaadaaOGaeiilaWIaeu4MdW0aaWbaaSqabeaacqaIWaamaaGccqGGPaqkcqGH9aqpdaaeWbqaamaaqahabaGaem4DaC3aaSbaaSqaaiabdEgaNjabdchaWjabdUgaRjabdUgaRjabcEcaNaqabaaabaGaem4AaSMaei4jaCIaeyypa0JaeGymaedabaGaem4saS0aaSbaaWqaaGGaciab=H7aXbqabaaaniabggHiLdaaleaacqWGRbWAcqGH9aqpcqaIXaqmaeaacqWGlbWsdaWgaaadbaGae83TdGgabeaaa0GaeyyeIuoakiabdgfarnaaBaaaleaacqWGNbWzcqWGWbaCcqWGRbWAcqWGRbWAcqGGNaWjaeqaaOGaeiilaWcaaa@69D3@

And, consequently.

Q(Θ,Θ0)=∑g=1G∑p=1Ω∑k=1Kη∑k'=1Kθngpwgpkk'Qgpkk'
 MathType@MTEF@5@5@+=feaafiart1ev1aaatCvAUfKttLearuWrP9MDH5MBPbIqV92AaeXatLxBI9gBaebbnrfifHhDYfgasaacH8akY=wiFfYdH8Gipec8Eeeu0xXdbba9frFj0=OqFfea0dXdd9vqai=hGuQ8kuc9pgc9s8qqaq=dirpe0xb9q8qiLsFr0=vr0=vr0dc8meaabaqaciaacaGaaeqabaqabeGadaaakeaacqWGrbqucqGGOaakcqqHyoqucqGGSaalcqqHyoqudaahaaWcbeqaaiabicdaWaaakiabcMcaPiabg2da9maaqahabaWaaabCaeaadaaeWbqaamaaqahabaGaemOBa42aaSbaaSqaaiabdEgaNjabdchaWbqabaGccqWG3bWDdaWgaaWcbaGaem4zaCMaemiCaaNaem4AaSMaem4AaSMaei4jaCcabeaakiabdgfarnaaBaaaleaacqWGNbWzcqWGWbaCcqWGRbWAcqWGRbWAcqGGNaWjaeqaaaqaaiabdUgaRjabcEcaNiabg2da9iabigdaXaqaaiabdUealnaaBaaameaaiiGacqWF4oqCaeqaaaqdcqGHris5aaWcbaGaem4AaSMaeyypa0JaeGymaedabaGaem4saS0aaSbaaWqaaiab=D7aObqabaaaniabggHiLdaaleaacqWGWbaCcqGH9aqpcqaIXaqmaeaacqqHPoWva0GaeyyeIuoaaSqaaiabdEgaNjabg2da9iabigdaXaqaaiabdEeahbqdcqGHris5aaaa@6953@

#### The E-step

In this step, the function *Q*(Θ, Θ^0^) or, equivalently, the set of coefficients *w*_*gpkk*' _and *Q*_*gpkk*' _is computed by using inward-outward recursion on the tree.

#### The inward (*γ*) recursion

Here we suggest a variation on the well-known Felsenstein's pruning algorithm [46]. Let us associate with each node *t *(except for the root) a vector γigpkk'(t)=Pr⁡(Vt|qtP=i,Ξ0,Ψgkk'0)
 MathType@MTEF@5@5@+=feaafiart1ev1aaatCvAUfKttLearuWrP9MDH5MBPbIqV92AaeXatLxBI9gBaebbnrfifHhDYfgasaacH8akY=wiFfYdH8Gipec8Eeeu0xXdbba9frFj0=OqFfea0dXdd9vqai=hGuQ8kuc9pgc9s8qqaq=dirpe0xb9q8qiLsFr0=vr0=vr0dc8meaabaqaciaacaGaaeqabaqabeGadaaakeaaiiGacqWFZoWzdaqhaaWcbaGaemyAaKgabaGaem4zaCMaemiCaaNaem4AaSMaem4AaSMaei4jaCcaaOGaeiikaGIaemiDaqNaeiykaKIaeyypa0JagiiuaaLaeiOCaiNaeiikaGIaemOvay1aaSbaaSqaaiabdsha0bqabaGccqGG8baFcqWGXbqCdaqhaaWcbaGaemiDaqhabaGaemiuaafaaOGaeyypa0JaemyAaKMaeiilaWIaeuONdG1aaWbaaSqabeaacqaIWaamaaGccqGGSaalcqqHOoqwdaqhaaWcbaGaem4zaCMaem4AaSMaem4AaSMaei4jaCcabaGaeGimaadaaOGaeiykaKcaaa@55B2@. This is the probability of observing the nodes *V*_*t *_(which are a subset of the pattern *ω*_*p*_) for a gene *g*, when the gain and loss rate variables are rkη
 MathType@MTEF@5@5@+=feaafiart1ev1aaatCvAUfKttLearuWrP9MDH5MBPbIqV92AaeXatLxBI9gBaebbnrfifHhDYfgasaacH8akY=wiFfYdH8Gipec8Eeeu0xXdbba9frFj0=OqFfea0dXdd9vqai=hGuQ8kuc9pgc9s8qqaq=dirpe0xb9q8qiLsFr0=vr0=vr0dc8meaabaqaciaacaGaaeqabaqabeGadaaakeaacqWGYbGCdaqhaaWcbaGaem4AaSgabaacciGae83TdGgaaaaa@3158@ and rk'θ
 MathType@MTEF@5@5@+=feaafiart1ev1aaatCvAUfKttLearuWrP9MDH5MBPbIqV92AaeXatLxBI9gBaebbnrfifHhDYfgasaacH8akY=wiFfYdH8Gipec8Eeeu0xXdbba9frFj0=OqFfea0dXdd9vqai=hGuQ8kuc9pgc9s8qqaq=dirpe0xb9q8qiLsFr0=vr0=vr0dc8meaabaqaciaacaGaaeqabaqabeGadaaakeaacqWGYbGCdaqhaaWcbaGaem4AaSMaei4jaCcabaacciGae8hUdehaaaaa@3238@, respectively, and the parent node of *t *is known to be in state *i*. By definition, this function is initialized at all leaves (*t *∈ *V*_0_) by

γ(t∈V0)={(1−ξt(1−e−ηgkΔt)1−(1−φt)e−θgk'Δt)qt=0(ξt(1−e−ηgkΔt)(1−φt)e−θgk'Δt)qt=1.
 MathType@MTEF@5@5@+=feaafiart1ev1aaatCvAUfKttLearuWrP9MDH5MBPbIqV92AaeXatLxBI9gBaebbnrfifHhDYfgasaacH8akY=wiFfYdH8Gipec8Eeeu0xXdbba9frFj0=OqFfea0dXdd9vqai=hGuQ8kuc9pgc9s8qqaq=dirpe0xb9q8qiLsFr0=vr0=vr0dc8meaabaqaciaacaGaaeqabaqabeGadaaakeaaiiGacqWFZoWzcqGGOaakcqWG0baDcqGHiiIZcqWGwbGvdaWgaaWcbaGaeGimaadabeaakiabcMcaPiabg2da9maaceqabaqbaeaabiGaaaqaamaabmaabaqbaeqabiqaaaqaaiabigdaXiabgkHiTiab=57a4naaBaaaleaacqWG0baDaeqaaOGaeiikaGIaeGymaeJaeyOeI0Iaemyzau2aaWbaaSqabeaacqGHsislcqWF3oaAdaWgaaadbaGaem4zaCMaem4AaSgabeaaliabfs5aenaaBaaameaacqWG0baDaeqaaaaakiabcMcaPaqaaiabigdaXiabgkHiTiabcIcaOiabigdaXiabgkHiTiab=z8aMnaaBaaaleaacqWG0baDaeqaaOGaeiykaKIaemyzau2aaWbaaSqabeaacqGHsislcqWF4oqCdaWgaaadbaGaem4zaCMaem4AaSMaei4jaCcabeaaliabfs5aenaaBaaameaacqWG0baDaeqaaaaaaaaakiaawIcacaGLPaaaaeaacqWGXbqCdaWgaaWcbaGaemiDaqhabeaakiabg2da9iabicdaWaqaamaabmaabaqbaeqabiqaaaqaaiab=57a4naaBaaaleaacqWG0baDaeqaaOGaeiikaGIaeGymaeJaeyOeI0Iaemyzau2aaWbaaSqabeaacqGHsislcqWF3oaAdaWgaaadbaGaem4zaCMaem4AaSgabeaaliabfs5aenaaBaaameaacqWG0baDaeqaaaaakiabcMcaPaqaaiabcIcaOiabigdaXiabgkHiTiab=z8aMnaaBaaaleaacqWG0baDaeqaaOGaeiykaKIaemyzau2aaWbaaSqabeaacqGHsislcqWF4oqCdaWgaaadbaGaem4zaCMaem4AaSMaei4jaCcabeaaliabfs5aenaaBaaameaacqWG0baDaeqaaaaaaaaakiaawIcacaGLPaaaaeaacqWGXbqCdaWgaaWcbaGaemiDaqhabeaakiabg2da9iabigdaXiabc6caUaaaaiaawUhaaaaa@8F1E@

Here and in the derivations to follow, we omit the superscript from *γ*. For all internal nodes (except for the root), *γ *is computed using the recursion

γi(t)=∑j=01Tij(g,t)γ˜j(t),
 MathType@MTEF@5@5@+=feaafiart1ev1aaatCvAUfKttLearuWrP9MDH5MBPbIqV92AaeXatLxBI9gBaebbnrfifHhDYfgasaacH8akY=wiFfYdH8Gipec8Eeeu0xXdbba9frFj0=OqFfea0dXdd9vqai=hGuQ8kuc9pgc9s8qqaq=dirpe0xb9q8qiLsFr0=vr0=vr0dc8meaabaqaciaacaGaaeqabaqabeGadaaakeaaiiGacqWFZoWzdaWgaaWcbaGaemyAaKgabeaakiabcIcaOiabdsha0jabcMcaPiabg2da9maaqahabaGaemivaq1aaSbaaSqaaiabdMgaPjabdQgaQbqabaGccqGGOaakcqWGNbWzcqGGSaalcqWG0baDcqGGPaqkcuWFZoWzgaacamaaBaaaleaacqWGQbGAaeqaaOGaeiikaGIaemiDaqNaeiykaKcaleaacqWGQbGAcqGH9aqpcqaIWaamaeaacqaIXaqma0GaeyyeIuoakiabcYcaSaaa@4B61@

where γ˜j(t)
 MathType@MTEF@5@5@+=feaafiart1ev1aaatCvAUfKttLearuWrP9MDH5MBPbIqV92AaeXatLxBI9gBaebbnrfifHhDYfgasaacH8akY=wiFfYdH8Gipec8Eeeu0xXdbba9frFj0=OqFfea0dXdd9vqai=hGuQ8kuc9pgc9s8qqaq=dirpe0xb9q8qiLsFr0=vr0=vr0dc8meaabaqaciaacaGaaeqabaqabeGadaaakeaaiiGacuWFZoWzgaacamaaBaaaleaacqWGQbGAaeqaaOGaeiikaGIaemiDaqNaeiykaKcaaa@331F@ is defined as *γ*_*j*_(L(*t*))*γ *_*j*_(R(*t*)). This is easy to see, as

γi(t)=Pr⁡(Vt|qtP=i)=Pr⁡(VtL,VtR|qtP=i)=∑j=01Pr⁡(VtL,VtR,qt=j|qtP=i)==∑j=01Pr⁡(qt=j|qtP=i)⋅Pr⁡(VtL|qt=j,qtP=i)⋅Pr⁡(VtR|VtL,qt=j,qtP=i).
 MathType@MTEF@5@5@+=feaafiart1ev1aaatCvAUfKttLearuWrP9MDH5MBPbIqV92AaeXatLxBI9gBaebbnrfifHhDYfgasaacH8akY=wiFfYdH8Gipec8Eeeu0xXdbba9frFj0=OqFfea0dXdd9vqai=hGuQ8kuc9pgc9s8qqaq=dirpe0xb9q8qiLsFr0=vr0=vr0dc8meaabaqaciaacaGaaeqabaqabeGadaaakqaabeqaaGGaciab=n7aNnaaBaaaleaacqWGPbqAaeqaaOGaeiikaGIaemiDaqNaeiykaKIaeyypa0JagiiuaaLaeiOCaiNaeiikaGIaemOvay1aaSbaaSqaaiabdsha0bqabaGccqGG8baFcqWGXbqCdaqhaaWcbaGaemiDaqhabaGaemiuaafaaOGaeyypa0JaemyAaKMaeiykaKIaeyypa0JagiiuaaLaeiOCaiNaeiikaGIaemOvay1aa0baaSqaaiabdsha0bqaaiabdYeambaakiabcYcaSiabdAfawnaaDaaaleaacqWG0baDaeaacqWGsbGuaaGccqGG8baFcqWGXbqCdaqhaaWcbaGaemiDaqhabaGaemiuaafaaOGaeyypa0JaemyAaKMaeiykaKIaeyypa0ZaaabCaeaacyGGqbaucqGGYbGCcqGGOaakcqWGwbGvdaqhaaWcbaGaemiDaqhabaGaemitaWeaaOGaeiilaWIaemOvay1aa0baaSqaaiabdsha0bqaaiabdkfasbaakiabcYcaSiabdghaXnaaBaaaleaacqWG0baDaeqaaOGaeyypa0JaemOAaOMaeiiFaWNaemyCae3aa0baaSqaaiabdsha0bqaaiabdcfaqbaakiabg2da9iabdMgaPjabcMcaPaWcbaGaemOAaOMaeyypa0JaeGimaadabaGaeGymaedaniabggHiLdGccqGH9aqpaeaacaWLjaGaeyypa0ZaaabCaeaacyGGqbaucqGGYbGCcqGGOaakcqWGXbqCdaWgaaWcbaGaemiDaqhabeaakiabg2da9iabdQgaQjabcYha8jabdghaXnaaDaaaleaacqWG0baDaeaacqWGqbauaaGccqGH9aqpcqWGPbqAcqGGPaqkaSqaaiabdQgaQjabg2da9iabicdaWaqaaiabigdaXaqdcqGHris5aOGaeyyXICTagiiuaaLaeiOCaiNaeiikaGIaemOvay1aa0baaSqaaiabdsha0bqaaiabdYeambaakiabcYha8jabdghaXnaaBaaaleaacqWG0baDaeqaaOGaeyypa0JaemOAaOMaeiilaWIaemyCae3aa0baaSqaaiabdsha0bqaaiabdcfaqbaakiabg2da9iabdMgaPjabcMcaPiabgwSixlGbccfaqjabckhaYjabcIcaOiabdAfawnaaDaaaleaacqWG0baDaeaacqWGsbGuaaGccqGG8baFcqWGwbGvdaqhaaWcbaGaemiDaqhabaGaemitaWeaaOGaeiilaWIaemyCae3aaSbaaSqaaiabdsha0bqabaGccqGH9aqpcqWGQbGAcqGGSaalcqWGXbqCdaqhaaWcbaGaemiDaqhabaGaemiuaafaaOGaeyypa0JaemyAaKMaeiykaKIaeiOla4caaaa@CFCB@

The first term is, simply, the definition of *T*_*ij *_(*g, t*). Given *q*_*t*_, VtL
 MathType@MTEF@5@5@+=feaafiart1ev1aaatCvAUfKttLearuWrP9MDH5MBPbIqV92AaeXatLxBI9gBaebbnrfifHhDYfgasaacH8akY=wiFfYdH8Gipec8Eeeu0xXdbba9frFj0=OqFfea0dXdd9vqai=hGuQ8kuc9pgc9s8qqaq=dirpe0xb9q8qiLsFr0=vr0=vr0dc8meaabaqaciaacaGaaeqabaqabeGadaaakeaacqWGwbGvdaqhaaWcbaGaemiDaqhabaGaemitaWeaaaaa@30A0@ is independent of qtP
 MathType@MTEF@5@5@+=feaafiart1ev1aaatCvAUfKttLearuWrP9MDH5MBPbIqV92AaeXatLxBI9gBaebbnrfifHhDYfgasaacH8akY=wiFfYdH8Gipec8Eeeu0xXdbba9frFj0=OqFfea0dXdd9vqai=hGuQ8kuc9pgc9s8qqaq=dirpe0xb9q8qiLsFr0=vr0=vr0dc8meaabaqaciaacaGaaeqabaqabeGadaaakeaacqWGXbqCdaqhaaWcbaGaemiDaqhabaGaemiuaafaaaaa@30DE@, thus the second term is just Pr(VtL
 MathType@MTEF@5@5@+=feaafiart1ev1aaatCvAUfKttLearuWrP9MDH5MBPbIqV92AaeXatLxBI9gBaebbnrfifHhDYfgasaacH8akY=wiFfYdH8Gipec8Eeeu0xXdbba9frFj0=OqFfea0dXdd9vqai=hGuQ8kuc9pgc9s8qqaq=dirpe0xb9q8qiLsFr0=vr0=vr0dc8meaabaqaciaacaGaaeqabaqabeGadaaakeaacqWGwbGvdaqhaaWcbaGaemiDaqhabaGaemitaWeaaaaa@30A0@ | *q*_*t *_= *j*) = *γ*_*j*_(*t*^*L*^) . By similar argument, the third term is simply Pr(VtR
 MathType@MTEF@5@5@+=feaafiart1ev1aaatCvAUfKttLearuWrP9MDH5MBPbIqV92AaeXatLxBI9gBaebbnrfifHhDYfgasaacH8akY=wiFfYdH8Gipec8Eeeu0xXdbba9frFj0=OqFfea0dXdd9vqai=hGuQ8kuc9pgc9s8qqaq=dirpe0xb9q8qiLsFr0=vr0=vr0dc8meaabaqaciaacaGaaeqabaqabeGadaaakeaacqWGwbGvdaqhaaWcbaGaemiDaqhabaGaemOuaifaaaaa@30AC@ | *q*_*t *_= *j*) = *γ*_*j*_(*t*^*R*^). Substituting these results in (14), we recover the recursion formula (13).

The *γ*-recursion allows for computing the likelihood of any observed pattern *ω*_*p*_, given the values of the rate variables:

Pr⁡(ωp|Ξ0,Ψgkk'0)=Pr⁡(V0|Ξ0,Ψgkk'0)=Pr⁡(V0L,V0R|Ξ0,Ψgkk'0)==∑i=01Pr⁡(V0L,V0R,q0=i|Ξ0,Ψgkk'0)==∑i=01Pr⁡(q0=i|Ξ0,Ψgkk'0)⋅Pr⁡(V0L|q0=i,Ξ0,Ψgkk'0)⋅Pr⁡(V0R|V0L,q0=i,Ξ0,Ψgkk'0).
 MathType@MTEF@5@5@+=feaafiart1ev1aaatCvAUfKttLearuWrP9MDH5MBPbIqV92AaeXatLxBI9gBaebbnrfifHhDYfgasaacH8akY=wiFfYdH8Gipec8Eeeu0xXdbba9frFj0=OqFfea0dXdd9vqai=hGuQ8kuc9pgc9s8qqaq=dirpe0xb9q8qiLsFr0=vr0=vr0dc8meaabaqaciaacaGaaeqabaqabeGadaaakqaabeqaaiGbccfaqjabckhaYjabcIcaOGGaciab=L8a3naaBaaaleaacqWGWbaCaeqaaOGaeiiFaWNaeuONdG1aaWbaaSqabeaacqaIWaamaaGccqGGSaalcqqHOoqwdaqhaaWcbaGaem4zaCMaem4AaSMaem4AaSMaei4jaCcabaGaeGimaadaaOGaeiykaKIaeyypa0JagiiuaaLaeiOCaiNaeiikaGIaemOvay1aaSbaaSqaaiabicdaWaqabaGccqGG8baFcqqHEoawdaahaaWcbeqaaiabicdaWaaakiabcYcaSiabfI6aznaaDaaaleaacqWGNbWzcqWGRbWAcqWGRbWAcqGGNaWjaeaacqaIWaamaaGccqGGPaqkcqGH9aqpcyGGqbaucqGGYbGCcqGGOaakcqWGwbGvdaqhaaWcbaGaeGimaadabaGaemitaWeaaOGaeiilaWIaemOvay1aa0baaSqaaiabicdaWaqaaiabdkfasbaakiabcYha8jabf65aynaaCaaaleqabaGaeGimaadaaOGaeiilaWIaeuiQdK1aa0baaSqaaiabdEgaNjabdUgaRjabdUgaRjabcEcaNaqaaiabicdaWaaakiabcMcaPiabg2da9aqaaiaaxMaacqGH9aqpdaaeWbqaaiGbccfaqjabckhaYjabcIcaOiabdAfawnaaDaaaleaacqaIWaamaeaacqWGmbataaGccqGGSaalcqWGwbGvdaqhaaWcbaGaeGimaadabaGaemOuaifaaOGaeiilaWIaemyCae3aaSbaaSqaaiabicdaWaqabaGccqGH9aqpcqWGPbqAcqGG8baFcqqHEoawdaahaaWcbeqaaiabicdaWaaakiabcYcaSiabfI6aznaaDaaaleaacqWGNbWzcqWGRbWAcqWGRbWAcqGGNaWjaeaacqaIWaamaaGccqGGPaqkaSqaaiabdMgaPjabg2da9iabicdaWaqaaiabigdaXaqdcqGHris5aOGaeyypa0dabaGaaCzcaiabg2da9maaqahabaGagiiuaaLaeiOCaiNaeiikaGIaemyCae3aaSbaaSqaaiabicdaWaqabaGccqGH9aqpcqWGPbqAcqGG8baFcqqHEoawdaahaaWcbeqaaiabicdaWaaakiabcYcaSiabfI6aznaaDaaaleaacqWGNbWzcqWGRbWAcqWGRbWAcqGGNaWjaeaacqaIWaamaaGccqGGPaqkcqGHflY1cyGGqbaucqGGYbGCcqGGOaakcqWGwbGvdaqhaaWcbaGaeGimaadabaGaemitaWeaaOGaeiiFaWNaemyCae3aaSbaaSqaaiabicdaWaqabaGccqGH9aqpcqWGPbqAcqGGSaalcqqHEoawdaahaaWcbeqaaiabicdaWaaakiabcYcaSiabfI6aznaaDaaaleaacqWGNbWzcqWGRbWAcqWGRbWAcqGGNaWjaeaacqaIWaamaaGccqGGPaqkcqGHflY1cyGGqbaucqGGYbGCcqGGOaakcqWGwbGvdaqhaaWcbaGaeGimaadabaGaemOuaifaaOGaeiiFaWNaemOvay1aa0baaSqaaiabicdaWaqaaiabdYeambaakiabcYcaSiabdghaXnaaBaaaleaacqaIWaamaeqaaOGaeyypa0JaemyAaKMaeiilaWIaeuONdG1aaWbaaSqabeaacqaIWaamaaGccqGGSaalcqqHOoqwdaqhaaWcbaGaem4zaCMaem4AaSMaem4AaSMaei4jaCcabaGaeGimaadaaOGaeiykaKcaleaacqWGPbqAcqGH9aqpcqaIWaamaeaacqaIXaqma0GaeyyeIuoakiabc6caUaaaaa@F487@

Given *q*_0_, V0R
 MathType@MTEF@5@5@+=feaafiart1ev1aaatCvAUfKttLearuWrP9MDH5MBPbIqV92AaeXatLxBI9gBaebbnrfifHhDYfgasaacH8akY=wiFfYdH8Gipec8Eeeu0xXdbba9frFj0=OqFfea0dXdd9vqai=hGuQ8kuc9pgc9s8qqaq=dirpe0xb9q8qiLsFr0=vr0=vr0dc8meaabaqaciaacaGaaeqabaqabeGadaaakeaacqWGwbGvdaqhaaWcbaGaeGimaadabaGaemOuaifaaaaa@3029@ is independent of V0L
 MathType@MTEF@5@5@+=feaafiart1ev1aaatCvAUfKttLearuWrP9MDH5MBPbIqV92AaeXatLxBI9gBaebbnrfifHhDYfgasaacH8akY=wiFfYdH8Gipec8Eeeu0xXdbba9frFj0=OqFfea0dXdd9vqai=hGuQ8kuc9pgc9s8qqaq=dirpe0xb9q8qiLsFr0=vr0=vr0dc8meaabaqaciaacaGaaeqabaqabeGadaaakeaacqWGwbGvdaqhaaWcbaGaeGimaadabaGaemitaWeaaaaa@301D@, and so

Pr⁡(V0R|V0L,q0=i,Ξ0,Ψgkk'0)=Pr⁡(V0R|q0=i,Ξ0,Ψgkk'0),
 MathType@MTEF@5@5@+=feaafiart1ev1aaatCvAUfKttLearuWrP9MDH5MBPbIqV92AaeXatLxBI9gBaebbnrfifHhDYfgasaacH8akY=wiFfYdH8Gipec8Eeeu0xXdbba9frFj0=OqFfea0dXdd9vqai=hGuQ8kuc9pgc9s8qqaq=dirpe0xb9q8qiLsFr0=vr0=vr0dc8meaabaqaciaacaGaaeqabaqabeGadaaakeaacyGGqbaucqGGYbGCcqGGOaakcqWGwbGvdaqhaaWcbaGaeGimaadabaGaemOuaifaaOGaeiiFaWNaemOvay1aa0baaSqaaiabicdaWaqaaiabdYeambaakiabcYcaSiabdghaXnaaBaaaleaacqaIWaamaeqaaOGaeyypa0JaemyAaKMaeiilaWIaeuONdG1aaWbaaSqabeaacqaIWaamaaGccqGGSaalcqqHOoqwdaqhaaWcbaGaem4zaCMaem4AaSMaem4AaSMaei4jaCcabaGaeGimaadaaOGaeiykaKIaeyypa0JagiiuaaLaeiOCaiNaeiikaGIaemOvay1aa0baaSqaaiabicdaWaqaaiabdkfasbaakiabcYha8jabdghaXnaaBaaaleaacqaIWaamaeqaaOGaeyypa0JaemyAaKMaeiilaWIaeuONdG1aaWbaaSqabeaacqaIWaamaaGccqGGSaalcqqHOoqwdaqhaaWcbaGaem4zaCMaem4AaSMaem4AaSMaei4jaCcabaGaeGimaadaaOGaeiykaKIaeiilaWcaaa@677B@

and

Pr⁡(ωp|Ξ0,Ψgkk'0)=∑i=01πiγ˜i(0).
 MathType@MTEF@5@5@+=feaafiart1ev1aaatCvAUfKttLearuWrP9MDH5MBPbIqV92AaeXatLxBI9gBaebbnrfifHhDYfgasaacH8akY=wiFfYdH8Gipec8Eeeu0xXdbba9frFj0=OqFfea0dXdd9vqai=hGuQ8kuc9pgc9s8qqaq=dirpe0xb9q8qiLsFr0=vr0=vr0dc8meaabaqaciaacaGaaeqabaqabeGadaaakeaacyGGqbaucqGGYbGCcqGGOaakiiGacqWFjpWDdaWgaaWcbaGaemiCaahabeaakiabcYha8jabf65aynaaCaaaleqabaGaeGimaadaaOGaeiilaWIaeuiQdK1aa0baaSqaaiabdEgaNjabdUgaRjabdUgaRjabcEcaNaqaaiabicdaWaaakiabcMcaPiabg2da9maaqahabaGae8hWda3aaSbaaSqaaiabdMgaPbqabaaabaGaemyAaKMaeyypa0JaeGimaadabaGaeGymaedaniabggHiLdGccuWFZoWzgaacamaaBaaaleaacqWGPbqAaeqaaOGaeiikaGIaeGimaaJaeiykaKIaeiOla4caaa@529A@

One of the useful features of this recursion is that is allows to treat missing data fairly easily. Only a single option has to be added to the initialization phase (12),

γ(t∈V0)=(11)qt=∗.
 MathType@MTEF@5@5@+=feaafiart1ev1aaatCvAUfKttLearuWrP9MDH5MBPbIqV92AaeXatLxBI9gBaebbnrfifHhDYfgasaacH8akY=wiFfYdH8Gipec8Eeeu0xXdbba9frFj0=OqFfea0dXdd9vqai=hGuQ8kuc9pgc9s8qqaq=dirpe0xb9q8qiLsFr0=vr0=vr0dc8meaabaqaciaacaGaaeqabaqabeGadaaakeaafaqabeqacaaabaacciGae83SdCMaeiikaGIaemiDaqNaeyicI4SaemOvay1aaSbaaSqaaiabicdaWaqabaGccqGGPaqkcqGH9aqpdaqadaqaauaabeqaceaaaeaacqaIXaqmaeaacqaIXaqmaaaacaGLOaGaayzkaaaabaGaemyCae3aaSbaaSqaaiabdsha0bqabaGccqGH9aqpcqGHxiIkcqGGUaGlaaaaaa@3FCE@

#### The outward (*α*-*β*) recursion

Once the *γ*-recursion is computed, we can use it to compute a second, complementary, recursion. To this end, let us associate with each node *t *(except for the root node) a matrix αijgpkk'(t)=Pr⁡(qt=j,qtP=i|ωp,Ξ0,Ψgkk'0)
 MathType@MTEF@5@5@+=feaafiart1ev1aaatCvAUfKttLearuWrP9MDH5MBPbIqV92AaeXatLxBI9gBaebbnrfifHhDYfgasaacH8akY=wiFfYdH8Gipec8Eeeu0xXdbba9frFj0=OqFfea0dXdd9vqai=hGuQ8kuc9pgc9s8qqaq=dirpe0xb9q8qiLsFr0=vr0=vr0dc8meaabaqaciaacaGaaeqabaqabeGadaaakeaaiiGacqWFXoqydaqhaaWcbaGaemyAaKMaemOAaOgabaGaem4zaCMaemiCaaNaem4AaSMaem4AaSMaei4jaCcaaOGaeiikaGIaemiDaqNaeiykaKIaeyypa0JagiiuaaLaeiOCaiNaeiikaGIaemyCae3aaSbaaSqaaiabdsha0bqabaGccqGH9aqpcqWGQbGAcqGGSaalcqWGXbqCdaqhaaWcbaGaemiDaqhabaGaemiuaafaaOGaeyypa0JaemyAaKMaeiiFaWNae8xYdC3aaSbaaSqaaiabdchaWbqabaGccqGGSaalcqqHEoawdaahaaWcbeqaaiabicdaWaaakiabcYcaSiabfI6aznaaDaaaleaacqWGNbWzcqWGRbWAcqWGRbWAcqGGNaWjaeaacqaIWaamaaGccqGGPaqkaaa@5DE7@. It is convenient to define for each node *t *(except for the root node) a vector βjgpkk'(t)=∑i=01αijgpkk'(t)=Pr⁡(qt=j|ωp,Ξ0,Ψgkk'0)
 MathType@MTEF@5@5@+=feaafiart1ev1aaatCvAUfKttLearuWrP9MDH5MBPbIqV92AaeXatLxBI9gBaebbnrfifHhDYfgasaacH8akY=wiFfYdH8Gipec8Eeeu0xXdbba9frFj0=OqFfea0dXdd9vqai=hGuQ8kuc9pgc9s8qqaq=dirpe0xb9q8qiLsFr0=vr0=vr0dc8meaabaqaciaacaGaaeqabaqabeGadaaakeaaiiGacqWFYoGydaqhaaWcbaGaemOAaOgabaGaem4zaCMaemiCaaNaem4AaSMaem4AaSMaei4jaCcaaOGaeiikaGIaemiDaqNaeiykaKIaeyypa0ZaaabmaeaacqWFXoqydaqhaaWcbaGaemyAaKMaemOAaOgabaGaem4zaCMaemiCaaNaem4AaSMaem4AaSMaei4jaCcaaOGaeiikaGIaemiDaqNaeiykaKIaeyypa0daleaacqWGPbqAcqGH9aqpcqaIWaamaeaacqaIXaqma0GaeyyeIuoakiGbccfaqjabckhaYjabcIcaOiabdghaXnaaBaaaleaacqWG0baDaeqaaOGaeyypa0JaemOAaOMaeiiFaWNae8xYdC3aaSbaaSqaaiabdchaWbqabaGccqGGSaalcqqHEoawdaahaaWcbeqaaiabicdaWaaakiabcYcaSiabfI6aznaaDaaaleaacqWGNbWzcqWGRbWAcqWGRbWAcqGGNaWjaeaacqaIWaamaaGccqGGPaqkaaa@6A62@. Upon the computation of *α*, *β *is readily computed, too. Again, omitting the superscripts, *α *can be initialized from its definition on the two direct descendants of the root,

α(D(0))=1Pr⁡(ωp|Ξ0,Ψgkk'0){(π0γ0(D¯(0))T00(g,D(0))0π1γ1(D¯(0))T10(g,D(0))0)D(0)∈V0,q0D=0(0π0γ0(D¯(0))T01(g,D(0))0π1γ1(D¯(0))T11(g,D(0)))D(0)∈V0,q0D=1(π0γ0(D¯(0))γ˜0(D(0))T00(g,D(0))π0γ0(D¯(0))γ˜1(D(0))T01(D(0))π1γ1(D¯(0))γ˜0(D(0))T10(D(0))π1γ1(D¯(0))γ˜1(D(0))T11(D(0)))D(0)∉V0.
 MathType@MTEF@5@5@+=feaafiart1ev1aaatCvAUfKttLearuWrP9MDH5MBPbIqV92AaeXatLxBI9gBaebbnrfifHhDYfgasaacH8akY=wiFfYdH8Gipec8Eeeu0xXdbba9frFj0=OqFfea0dXdd9vqai=hGuQ8kuc9pgc9s8qqaq=dirpe0xb9q8qiLsFr0=vr0=vr0dc8meaabaqaciaacaGaaeqabaqabeGadaaakeaaiiGaju28aiab=f7aHjabcIcaOiabbseaejabcIcaOiabicdaWiabcMcaPiabcMcaPiabg2da9WWaaSaaaKqzZdqaaiabigdaXaqaaiGbccfaqjabckhaYjabcIcaOiab=L8a3XWaaSbaaKqzZdqaaiabdchaWbqabaGaeiiFaWNaeuONdGfddaahaaqcLnpabeqaaiabicdaWaaacqGGSaalcqqHOoqwmmaaDaaaju28aeaacqWGNbWzcqWGRbWAcqWGRbWAcqGGNaWjaeaacqaIWaamaaGaeiykaKcaaWWaaiqabeaafaqaaeWacaaabaWaaeWaaeaafaqabeGabaaabaqbaeqabeGaaaqaaiab=b8aWnaaBaaabaGaeGimaadabeaacqWFZoWzdaWgaaqaaiabicdaWaqabaGaeiikaGIafeiraqKbaebacqGGOaakcqaIWaamcqGGPaqkcqGGPaqkcqWGubavdaWgaaqaaiabicdaWiabicdaWaqabaGaeiikaGIaem4zaCMaeiilaWIaeeiraqKaeiikaGIaeGimaaJaeiykaKIaeiykaKcabaGaeGimaadaaaqaauaabeqabiaaaeaacqWFapaCdaWgaaqaaiabigdaXaqabaGae83SdC2aaSbaaeaacqaIXaqmaeqaaiabcIcaOiqbbseaezaaraGaeiikaGIaeGimaaJaeiykaKIaeiykaKIaemivaq1aaSbaaeaacqaIXaqmcqaIWaamaeqaaiabcIcaOiabdEgaNjabcYcaSiabbseaejabcIcaOiabicdaWiabcMcaPiabcMcaPaqaaiabicdaWaaaaaaacaGLOaGaayzkaaaabaGaeeiraqKaeiikaGIaeGimaaJaeiykaKIaeyicI4SaemOvay1aaSbaaeaacqaIWaamaeqaaiabcYcaSiabdghaXnaaDaaabaGaeGimaadabaGaemiraqeaaiabg2da9iabicdaWaqaamaabmaabaqbaeaabiqaaaqaauaabeqabiaaaeaacqaIWaamaeaacqWFapaCdaWgaaqaaiabicdaWaqabaGae83SdC2aaSbaaeaacqaIWaamaeqaaiabcIcaOiqbbseaezaaraGaeiikaGIaeGimaaJaeiykaKIaeiykaKIaemivaq1aaSbaaeaacqaIWaamcqaIXaqmaeqaaiabcIcaOiabdEgaNjabcYcaSiabbseaejabcIcaOiabicdaWiabcMcaPiabcMcaPaaaaeaafaqabeqacaaabaGaeGimaadabaGae8hWda3aaSbaaeaacqaIXaqmaeqaaiab=n7aNnaaBaaabaGaeGymaedabeaacqGGOaakcuqGebargaqeaiabcIcaOiabicdaWiabcMcaPiabcMcaPiabdsfaunaaBaaabaGaeGymaeJaeGymaedabeaacqGGOaakcqWGNbWzcqGGSaalcqqGebarcqGGOaakcqaIWaamcqGGPaqkcqGGPaqkaaaaaaGaayjkaiaawMcaaaqaaiabbseaejabcIcaOiabicdaWiabcMcaPiabgIGiolabdAfawnaaBaaabaGaeGimaadabeaacqGGSaalcqWGXbqCdaqhaaqaaiabicdaWaqaaiabdseaebaacqGH9aqpcqaIXaqmaeaadaqadaqaauaabeqaciaaaeaacqWFapaCdaWgaaqaaiabicdaWaqabaGae83SdC2aaSbaaeaacqaIWaamaeqaaiabcIcaOiqbbseaezaaraGaeiikaGIaeGimaaJaeiykaKIaeiykaKIaf83SdCMbaGaadaWgaaqaaiabicdaWaqabaGaeiikaGIaeeiraqKaeiikaGIaeGimaaJaeiykaKIaeiykaKIaemivaq1aaSbaaeaacqaIWaamcqaIWaamaeqaaiabcIcaOiabdEgaNjabcYcaSiabbseaejabcIcaOiabicdaWiabcMcaPiabcMcaPaqaaiab=b8aWnaaBaaabaGaeGimaadabeaacqWFZoWzdaWgaaqaaiabicdaWaqabaGaeiikaGIafeiraqKbaebacqGGOaakcqaIWaamcqGGPaqkcqGGPaqkcuWFZoWzgaacamaaBaaabaGaeGymaedabeaacqGGOaakcqqGebarcqGGOaakcqaIWaamcqGGPaqkcqGGPaqkcqWGubavdaWgaaqaaiabicdaWiabigdaXaqabaGaeiikaGIaeeiraqKaeiikaGIaeGimaaJaeiykaKIaeiykaKcabaGae8hWda3aaSbaaeaacqaIXaqmaeqaaiab=n7aNnaaBaaabaGaeGymaedabeaacqGGOaakcuqGebargaqeaiabcIcaOiabicdaWiabcMcaPiabcMcaPiqb=n7aNzaaiaWaaSbaaeaacqaIWaamaeqaaiabcIcaOiabbseaejabcIcaOiabicdaWiabcMcaPiabcMcaPiabdsfaunaaBaaabaGaeGymaeJaeGimaadabeaacqGGOaakcqqGebarcqGGOaakcqaIWaamcqGGPaqkcqGGPaqkaeaacqWFapaCdaWgaaqaaiabigdaXaqabaGae83SdC2aaSbaaeaacqaIXaqmaeqaaiabcIcaOiqbbseaezaaraGaeiikaGIaeGimaaJaeiykaKIaeiykaKIaf83SdCMbaGaadaWgaaqaaiabigdaXaqabaGaeiikaGIaeeiraqKaeiikaGIaeGimaaJaeiykaKIaeiykaKIaemivaq1aaSbaaeaacqaIXaqmcqaIXaqmaeqaaiabcIcaOiabbseaejabcIcaOiabicdaWiabcMcaPiabcMcaPaaaaiaawIcacaGLPaaaaeaacqqGebarcqGGOaakcqaIWaamcqGGPaqkcqGHjiYZcqWGwbGvdaWgaaqaaiabicdaWaqabaGaeiOla4caaaGaay5Eaaaaaa@4697@

Here, D(0) stands for any one of the direct descendants of the root, and D¯(0)
 MathType@MTEF@5@5@+=feaafiart1ev1aaatCvAUfKttLearuWrP9MDH5MBPbIqV92AaeXatLxBI9gBaebbnrfifHhDYfgasaacH8akY=wiFfYdH8Gipec8Eeeu0xXdbba9frFj0=OqFfea0dXdd9vqai=hGuQ8kuc9pgc9s8qqaq=dirpe0xb9q8qiLsFr0=vr0=vr0dc8meaabaqaciaacaGaaeqabaqabeGadaaakeaacuqGebargaqeaiabcIcaOiabicdaWiabcMcaPaaa@3073@ is its sibling. For any other internal node, *α *is computed using the outward-recursion

α(t)=(β0(P(t))γ˜0(t)T00(g,t)/γ0(t)β0(P(t))γ˜1(t)T01(g,t)/γ0(t)β1(P(t))γ˜0(t)T10(g,t)/γ1(t)β1(P(t))γ˜1(t)T11(g,t)/γ1(t)).
 MathType@MTEF@5@5@+=feaafiart1ev1aaatCvAUfKttLearuWrP9MDH5MBPbIqV92AaeXatLxBI9gBaebbnrfifHhDYfgasaacH8akY=wiFfYdH8Gipec8Eeeu0xXdbba9frFj0=OqFfea0dXdd9vqai=hGuQ8kuc9pgc9s8qqaq=dirpe0xb9q8qiLsFr0=vr0=vr0dc8meaabaqaciaacaGaaeqabaqabeGadaaakeaaiiGacqWFXoqycqGGOaakcqWG0baDcqGGPaqkcqGH9aqpdaqadaqaauaabeqaciaaaeaacqWFYoGydaWgaaWcbaGaeGimaadabeaakiabcIcaOiabbcfaqjabcIcaOiabdsha0jabcMcaPiabcMcaPiqb=n7aNzaaiaWaaSbaaSqaaiabicdaWaqabaGccqGGOaakcqWG0baDcqGGPaqkcqWGubavdaWgaaWcbaGaeGimaaJaeGimaadabeaakiabcIcaOiabdEgaNjabcYcaSiabdsha0jabcMcaPiabc+caViab=n7aNnaaBaaaleaacqaIWaamaeqaaOGaeiikaGIaemiDaqNaeiykaKcabaGae8NSdi2aaSbaaSqaaiabicdaWaqabaGccqGGOaakcqqGqbaucqGGOaakcqWG0baDcqGGPaqkcqGGPaqkcuWFZoWzgaacamaaBaaaleaacqaIXaqmaeqaaOGaeiikaGIaemiDaqNaeiykaKIaemivaq1aaSbaaSqaaiabicdaWiabigdaXaqabaGccqGGOaakcqWGNbWzcqGGSaalcqWG0baDcqGGPaqkcqGGVaWlcqWFZoWzdaWgaaWcbaGaeGimaadabeaakiabcIcaOiabdsha0jabcMcaPaqaaiab=j7aInaaBaaaleaacqaIXaqmaeqaaOGaeiikaGIaeeiuaaLaeiikaGIaemiDaqNaeiykaKIaeiykaKIaf83SdCMbaGaadaWgaaWcbaGaeGimaadabeaakiabcIcaOiabdsha0jabcMcaPiabdsfaunaaBaaaleaacqaIXaqmcqaIWaamaeqaaOGaeiikaGIaem4zaCMaeiilaWIaemiDaqNaeiykaKIaei4la8Iae83SdC2aaSbaaSqaaiabigdaXaqabaGccqGGOaakcqWG0baDcqGGPaqkaeaacqWFYoGydaWgaaWcbaGaeGymaedabeaakiabcIcaOiabbcfaqjabcIcaOiabdsha0jabcMcaPiabcMcaPiqb=n7aNzaaiaWaaSbaaSqaaiabigdaXaqabaGccqGGOaakcqWG0baDcqGGPaqkcqWGubavdaWgaaWcbaGaeGymaeJaeGymaedabeaakiabcIcaOiabdEgaNjabcYcaSiabdsha0jabcMcaPiabc+caViab=n7aNnaaBaaaleaacqaIXaqmaeqaaOGaeiikaGIaemiDaqNaeiykaKcaaaGaayjkaiaawMcaaiabc6caUaaa@AD8C@

To prove this recursion, let us start with the definition of *α*,

αij(t)=Pr⁡(qt=j,qtP=i|ωp)=Pr⁡(qt=j,qtP=i|V0)=Pr⁡(qtP=i|V0)⋅Pr⁡(qt=j|qtP=i,V0)=βi(P(t))⋅Pr⁡(qt=j|qtP=i,V0),
 MathType@MTEF@5@5@+=feaafiart1ev1aaatCvAUfKttLearuWrP9MDH5MBPbIqV92AaeXatLxBI9gBaebbnrfifHhDYfgasaacH8akY=wiFfYdH8Gipec8Eeeu0xXdbba9frFj0=OqFfea0dXdd9vqai=hGuQ8kuc9pgc9s8qqaq=dirpe0xb9q8qiLsFr0=vr0=vr0dc8meaabaqaciaacaGaaeqabaqabeGadaaakqaabeqaaGGacKqzafGae8xSde2cdaWgaaqaaKqzafGaemyAaKMaemOAaOgaleqaaKqzafGaeiikaGIaemiDaqNaeiykaKIaeyypa0JagiiuaaLaeiOCaiNaeiikaGIaemyCae3cdaWgaaqaaKqzafGaemiDaqhaleqaaKqzafGaeyypa0JaemOAaOMaeiilaWIaemyCae3cdaqhaaqaaKqzafGaemiDaqhaleaajugqbiabdcfaqbaacqGH9aqpcqWGPbqAcqGG8baFcqWFjpWDlmaaBaaabaqcLbuacqWGWbaCaSqabaqcLbuacqGGPaqkcqGH9aqpcyGGqbaucqGGYbGCcqGGOaakcqWGXbqClmaaBaaabaqcLbuacqWG0baDaSqabaqcLbuacqGH9aqpcqWGQbGAcqGGSaalcqWGXbqClmaaDaaabaqcLbuacqWG0baDaSqaaKqzafGaemiuaafaaiabg2da9iabdMgaPjabcYha8jabdAfawTWaaSbaaeaajugqbiabicdaWaWcbeaajugqbiabcMcaPiabg2da9aGcbaqcLbuacaWLjaGagiiuaaLaeiOCaiNaeiikaGIaemyCae3cdaqhaaqaaKqzafGaemiDaqhaleaajugqbiabdcfaqbaacqGH9aqpcqWGPbqAcqGG8baFcqWGwbGvlmaaBaaabaqcLbuacqaIWaamaSqabaqcLbuacqGGPaqkcqGHflY1cyGGqbaucqGGYbGCcqGGOaakcqWGXbqClmaaBaaabaqcLbuacqWG0baDaSqabaqcLbuacqGH9aqpcqWGQbGAcqGG8baFcqWGXbqClmaaDaaabaqcLbuacqWG0baDaSqaaKqzafGaemiuaafaaiabg2da9iabdMgaPjabcYcaSiabdAfawTWaaSbaaeaajugqbiabicdaWaWcbeaajugqbiabcMcaPiabg2da9iab=j7aITWaaSbaaeaajugqbiabdMgaPbWcbeaajugqbiabcIcaOiabbcfaqjabcIcaOiabdsha0jabcMcaPiabcMcaPiabgwSixlGbccfaqjabckhaYjabcIcaOiabdghaXTWaaSbaaeaajugqbiabdsha0bWcbeaajugqbiabg2da9iabdQgaQjabcYha8jabdghaXTWaa0baaeaajugqbiabdsha0bWcbaqcLbuacqWGqbauaaGaeyypa0JaemyAaKMaeiilaWIaemOvay1cdaWgaaqaaKqzafGaeGimaadaleqaaKqzafGaeiykaKIaeiilaWcaaaa@C331@

and make the decomposition *V*_0 _= *V*_*t *_+ V¯t
 MathType@MTEF@5@5@+=feaafiart1ev1aaatCvAUfKttLearuWrP9MDH5MBPbIqV92AaeXatLxBI9gBaebbnrfifHhDYfgasaacH8akY=wiFfYdH8Gipec8Eeeu0xXdbba9frFj0=OqFfea0dXdd9vqai=hGuQ8kuc9pgc9s8qqaq=dirpe0xb9q8qiLsFr0=vr0=vr0dc8meaabaqaciaacaGaaeqabaqabeGadaaakeaacuWGwbGvgaqeamaaBaaaleaacqWG0baDaeqaaaaa@2F96@, with V¯t
 MathType@MTEF@5@5@+=feaafiart1ev1aaatCvAUfKttLearuWrP9MDH5MBPbIqV92AaeXatLxBI9gBaebbnrfifHhDYfgasaacH8akY=wiFfYdH8Gipec8Eeeu0xXdbba9frFj0=OqFfea0dXdd9vqai=hGuQ8kuc9pgc9s8qqaq=dirpe0xb9q8qiLsFr0=vr0=vr0dc8meaabaqaciaacaGaaeqabaqabeGadaaakeaacuWGwbGvgaqeamaaBaaaleaacqWG0baDaeqaaaaa@2F96@ being the set of all leaves such that node *t *is not among their ancestors. But, given qtP
 MathType@MTEF@5@5@+=feaafiart1ev1aaatCvAUfKttLearuWrP9MDH5MBPbIqV92AaeXatLxBI9gBaebbnrfifHhDYfgasaacH8akY=wiFfYdH8Gipec8Eeeu0xXdbba9frFj0=OqFfea0dXdd9vqai=hGuQ8kuc9pgc9s8qqaq=dirpe0xb9q8qiLsFr0=vr0=vr0dc8meaabaqaciaacaGaaeqabaqabeGadaaakeaacqWGXbqCdaqhaaWcbaGaemiDaqhabaGaemiuaafaaaaa@30DE@, the state of node *t *is independent on V¯t
 MathType@MTEF@5@5@+=feaafiart1ev1aaatCvAUfKttLearuWrP9MDH5MBPbIqV92AaeXatLxBI9gBaebbnrfifHhDYfgasaacH8akY=wiFfYdH8Gipec8Eeeu0xXdbba9frFj0=OqFfea0dXdd9vqai=hGuQ8kuc9pgc9s8qqaq=dirpe0xb9q8qiLsFr0=vr0=vr0dc8meaabaqaciaacaGaaeqabaqabeGadaaakeaacuWGwbGvgaqeamaaBaaaleaacqWG0baDaeqaaaaa@2F96@, and therefore (19) becomes

αij(t)=βi(P(t))⋅Pr⁡(qt=j|qtP=i,Vt)
 MathType@MTEF@5@5@+=feaafiart1ev1aaatCvAUfKttLearuWrP9MDH5MBPbIqV92AaeXatLxBI9gBaebbnrfifHhDYfgasaacH8akY=wiFfYdH8Gipec8Eeeu0xXdbba9frFj0=OqFfea0dXdd9vqai=hGuQ8kuc9pgc9s8qqaq=dirpe0xb9q8qiLsFr0=vr0=vr0dc8meaabaqaciaacaGaaeqabaqabeGadaaakeaaiiGacqWFXoqydaWgaaWcbaGaemyAaKMaemOAaOgabeaakiabcIcaOiabdsha0jabcMcaPiabg2da9iab=j7aInaaBaaaleaacqWGPbqAaeqaaOGaeiikaGIaeeiuaaLaeiikaGIaemiDaqNaeiykaKIaeiykaKIaeyyXICTagiiuaaLaeiOCaiNaeiikaGIaemyCae3aaSbaaSqaaiabdsha0bqabaGccqGH9aqpcqWGQbGAcqGG8baFcqWGXbqCdaqhaaWcbaGaemiDaqhabaGaemiuaafaaOGaeyypa0JaemyAaKMaeiilaWIaemOvay1aaSbaaSqaaiabdsha0bqabaGccqGGPaqkaaa@5672@

From Bayes formula,

Pr⁡(qt=j|qtP=i,Vt)=Pr⁡(qt=j,Vt|qtP=i)Pr⁡(Vt|qtP=i)=Pr⁡(qt=j|qtP=i)⋅Pr⁡(Vt|qt=j,qtP=i)γi(t)=Tij(g,t)γi(t)⋅Pr⁡(Vt|qt=j,qtP=i).
 MathType@MTEF@5@5@+=feaafiart1ev1aaatCvAUfKttLearuWrP9MDH5MBPbIqV92AaeXatLxBI9gBaebbnrfifHhDYfgasaacH8akY=wiFfYdH8Gipec8Eeeu0xXdbba9frFj0=OqFfea0dXdd9vqai=hGuQ8kuc9pgc9s8qqaq=dirpe0xb9q8qiLsFr0=vr0=vr0dc8meaabaqaciaacaGaaeqabaqabeGadaaakqaabeqaaiGbccfaqjabckhaYjabcIcaOiabdghaXnaaBaaaleaacqWG0baDaeqaaOGaeyypa0JaemOAaOMaeiiFaWNaemyCae3aa0baaSqaaiabdsha0bqaaiabdcfaqbaakiabg2da9iabdMgaPjabcYcaSiabdAfawnaaBaaaleaacqWG0baDaeqaaOGaeiykaKIaeyypa0ZaaSaaaeaacyGGqbaucqGGYbGCcqGGOaakcqWGXbqCdaWgaaWcbaGaemiDaqhabeaakiabg2da9iabdQgaQjabcYcaSiabdAfawnaaBaaaleaacqWG0baDaeqaaOGaeiiFaWNaemyCae3aa0baaSqaaiabdsha0bqaaiabdcfaqbaakiabg2da9iabdMgaPjabcMcaPaqaaiGbccfaqjabckhaYjabcIcaOiabdAfawnaaBaaaleaacqWG0baDaeqaaOGaeiiFaWNaemyCae3aa0baaSqaaiabdsha0bqaaiabdcfaqbaakiabg2da9iabdMgaPjabcMcaPaaacqGH9aqpaeaacaWLjaWaaSaaaeaacyGGqbaucqGGYbGCcqGGOaakcqWGXbqCdaWgaaWcbaGaemiDaqhabeaakiabg2da9iabdQgaQjabcYha8jabdghaXnaaDaaaleaacqWG0baDaeaacqWGqbauaaGccqGH9aqpcqWGPbqAcqGGPaqkcqGHflY1cyGGqbaucqGGYbGCcqGGOaakcqWGwbGvdaWgaaWcbaGaemiDaqhabeaakiabcYha8jabdghaXnaaBaaaleaacqWG0baDaeqaaOGaeyypa0JaemOAaOMaeiilaWIaemyCae3aa0baaSqaaiabdsha0bqaaiabdcfaqbaakiabg2da9iabdMgaPjabcMcaPaqaaGGaciab=n7aNnaaBaaaleaacqWGPbqAaeqaaOGaeiikaGIaemiDaqNaeiykaKcaaiabg2da9maalaaabaGaemivaq1aaSbaaSqaaiabdMgaPjabdQgaQbqabaGccqGGOaakcqWGNbWzcqGGSaalcqWG0baDcqGGPaqkaeaacqWFZoWzdaWgaaWcbaGaemyAaKgabeaakiabcIcaOiabdsha0jabcMcaPaaacqGHflY1cyGGqbaucqGGYbGCcqGGOaakcqWGwbGvdaWgaaWcbaGaemiDaqhabeaakiabcYha8jabdghaXnaaBaaaleaacqWG0baDaeqaaOGaeyypa0JaemOAaOMaeiilaWIaemyCae3aa0baaSqaaiabdsha0bqaaiabdcfaqbaakiabg2da9iabdMgaPjabcMcaPiabc6caUaaaaa@C3B3@

But, given *q*_*t*_, *V*_*t *_is independent of P(*t*) and therefore

Pr⁡(Vt|qt=j,qtP=i)=Pr⁡(Vt|qt=j)=γ˜j(t).
 MathType@MTEF@5@5@+=feaafiart1ev1aaatCvAUfKttLearuWrP9MDH5MBPbIqV92AaeXatLxBI9gBaebbnrfifHhDYfgasaacH8akY=wiFfYdH8Gipec8Eeeu0xXdbba9frFj0=OqFfea0dXdd9vqai=hGuQ8kuc9pgc9s8qqaq=dirpe0xb9q8qiLsFr0=vr0=vr0dc8meaabaqaciaacaGaaeqabaqabeGadaaakeaacyGGqbaucqGGYbGCcqGGOaakcqWGwbGvdaWgaaWcbaGaemiDaqhabeaakiabcYha8jabdghaXnaaBaaaleaacqWG0baDaeqaaOGaeyypa0JaemOAaOMaeiilaWIaemyCae3aa0baaSqaaiabdsha0bqaaiabdcfaqbaakiabg2da9iabdMgaPjabcMcaPiabg2da9iGbccfaqjabckhaYjabcIcaOiabdAfawnaaBaaaleaacqWG0baDaeqaaOGaeiiFaWNaemyCae3aaSbaaSqaaiabdsha0bqabaGccqGH9aqpcqWGQbGAcqGGPaqkcqGH9aqpiiGacuWFZoWzgaacamaaBaaaleaacqWGQbGAaeqaaOGaeiikaGIaemiDaqNaeiykaKIaeiOla4caaa@59BE@

Combining (22) and (21) in (20), we get

αij(t)=γ˜j(t)βi(P(t))γi(t)Tij(g,p),
 MathType@MTEF@5@5@+=feaafiart1ev1aaatCvAUfKttLearuWrP9MDH5MBPbIqV92AaeXatLxBI9gBaebbnrfifHhDYfgasaacH8akY=wiFfYdH8Gipec8Eeeu0xXdbba9frFj0=OqFfea0dXdd9vqai=hGuQ8kuc9pgc9s8qqaq=dirpe0xb9q8qiLsFr0=vr0=vr0dc8meaabaqaciaacaGaaeqabaqabeGadaaakeaaiiGacqWFXoqydaWgaaWcbaGaemyAaKMaemOAaOgabeaakiabcIcaOiabdsha0jabcMcaPiabg2da9maalaaabaGaf83SdCMbaGaadaWgaaWcbaGaemOAaOgabeaakiabcIcaOiabdsha0jabcMcaPiab=j7aInaaBaaaleaacqWGPbqAaeqaaOGaeiikaGIaeeiuaaLaeiikaGIaemiDaqNaeiykaKIaeiykaKcabaGae83SdC2aaSbaaSqaaiabdMgaPbqabaGccqGGOaakcqWG0baDcqGGPaqkaaGaemivaq1aaSbaaSqaaiabdMgaPjabdQgaQbqabaGccqGGOaakcqWGNbWzcqGGSaalcqWGWbaCcqGGPaqkcqGGSaalaaa@55B0@

which is just another form of (18). Finally, for each leaf that is not a descendant of the root

α(t)={(β0(P(t))0β1(P(t))0)qt=0(0β0(P(t))0β1(P(t)))qt=1.t∈V0,P(t)≠0
 MathType@MTEF@5@5@+=feaafiart1ev1aaatCvAUfKttLearuWrP9MDH5MBPbIqV92AaeXatLxBI9gBaebbnrfifHhDYfgasaacH8akY=wiFfYdH8Gipec8Eeeu0xXdbba9frFj0=OqFfea0dXdd9vqai=hGuQ8kuc9pgc9s8qqaq=dirpe0xb9q8qiLsFr0=vr0=vr0dc8meaabaqaciaacaGaaeqabaqabeGadaaakeaaiiGacqWFXoqycqGGOaakcqWG0baDcqGGPaqkcqGH9aqpdaGabaqaauaabeqabiaaaeaafaqabeGabaaabaqbaeqabeGaaaqaamaabmaabaqbaeqabiGaaaqaaiab=j7aInaaBaaabaGaeGimaadabeaacqGGOaakcqqGqbaucqGGOaakcqWG0baDcqGGPaqkcqGGPaqkaeaacqaIWaamaeaacqWFYoGydaWgaaqaaiabigdaXaqabaGaeiikaGIaeeiuaaLaeiikaGIaemiDaqNaeiykaKIaeiykaKcabaGaeGimaadaaaGaayjkaiaawMcaaaqaaiabdghaXnaaBaaaleaacqWG0baDaeqaaOGaeyypa0JaeGimaadaaaqaauaabeqabiaaaeaadaqadaqaauaabeqaciaaaeaacqaIWaamaeaacqWFYoGydaWgaaqaaiabicdaWaqabaGaeiikaGIaeeiuaaLaeiikaGIaemiDaqNaeiykaKIaeiykaKcabaGaeGimaadabaGae8NSdi2aaSbaaeaacqaIXaqmaeqaaiabcIcaOiabbcfaqjabcIcaOiabdsha0jabcMcaPiabcMcaPaaaaiaawIcacaGLPaaaaeaacqWGXbqCdaWgaaWcbaGaemiDaqhabeaakiabg2da9iabigdaXiabc6caUaaaaaaabaGaemiDaqNaeyicI4SaemOvay1aaSbaaSqaaiabicdaWaqabaGccqGGSaalcqqGqbaucqGGOaakcqWG0baDcqGGPaqkcqGHGjsUcqaIWaamaaaacaGL7baaaaa@7572@

When missing data are present, two simple modifications are required. First, we have to add to the initialization phase (17) an option

α(D(0))=1Pr⁡(ωp|Ξ0,Ψgkk'0)(π0γ0(D¯(0))T00(g,D(0))π0γ0(D¯(0))T01(D(0))π1γ1(D¯(0))T10(D(0))π1γ1(D¯(0))T11(D(0)))D(0)∈V0,q0D=∗
 MathType@MTEF@5@5@+=feaafiart1ev1aaatCvAUfKttLearuWrP9MDH5MBPbIqV92AaeXatLxBI9gBaebbnrfifHhDYfgasaacH8akY=wiFfYdH8Gipec8Eeeu0xXdbba9frFj0=OqFfea0dXdd9vqai=hGuQ8kuc9pgc9s8qqaq=dirpe0xb9q8qiLsFr0=vr0=vr0dc8meaabaqaciaacaGaaeqabaqabeGadaaakeaafaqabeqacaaabaacciqcLnpacqWFXoqycqGGOaakcqqGebarcqGGOaakcqaIWaamcqGGPaqkcqGGPaqkcqGH9aqpmmaalaaaju28aeaacqaIXaqmaeaacyGGqbaucqGGYbGCcqGGOaakcqWFjpWDmmaaBaaaju28aeaacqWGWbaCaeqaaiabcYha8jabf65ayXWaaWbaaKqzZdqabeaacqaIWaamaaGaeiilaWIaeuiQdKfddaqhaaqcLnpabaGaem4zaCMaem4AaSMaem4AaSMaei4jaCcabaGaeGimaadaaiabcMcaPaaammaabmaakeaaju28auaabeqaciaaaOqaaKqzZdGae8hWdahddaWgaaWcbaqcLnpacqaIWaamaSqabaqcLnpacqWFZoWzmmaaBaaaleaaju28aiabicdaWaWcbeaaju28aiabcIcaOiqbbseaezaaraGaeiikaGIaeGimaaJaeiykaKIaeiykaKIaemivaqfddaWgaaqcLnpabaGaeGimaaJaeGimaadabeaacqGGOaakcqWGNbWzcqGGSaalcqqGebarcqGGOaakcqaIWaamcqGGPaqkcqGGPaqkaOqaaKqzZdGae8hWdahddaWgaaWcbaqcLnpacqaIWaamaSqabaqcLnpacqWFZoWzmmaaBaaaleaaju28aiabicdaWaWcbeaaju28aiabcIcaOiqbbseaezaaraGaeiikaGIaeGimaaJaeiykaKIaeiykaKIaemivaqfddaWgaaqcLnpabaGaeGimaaJaeGymaedabeaacqGGOaakcqqGebarcqGGOaakcqaIWaamcqGGPaqkcqGGPaqkaOqaaKqzZdGae8hWdahddaWgaaWcbaqcLnpacqaIXaqmaSqabaqcLnpacqWFZoWzmmaaBaaaleaaju28aiabigdaXaWcbeaaju28aiabcIcaOiqbbseaezaaraGaeiikaGIaeGimaaJaeiykaKIaeiykaKIaemivaqfddaWgaaqcLnpabaGaeGymaeJaeGimaadabeaacqGGOaakcqqGebarcqGGOaakcqaIWaamcqGGPaqkcqGGPaqkaOqaaKqzZdGae8hWdahddaWgaaWcbaqcLnpacqaIXaqmaSqabaqcLnpacqWFZoWzmmaaBaaaleaaju28aiabigdaXaWcbeaaju28aiabcIcaOiqbbseaezaaraGaeiikaGIaeGimaaJaeiykaKIaeiykaKIaemivaqfddaWgaaqcLnpabaGaeGymaeJaeGymaedabeaacqGGOaakcqqGebarcqGGOaakcqaIWaamcqGGPaqkcqGGPaqkaaaakiaawIcacaGLPaaaaeaaju28aiabbseaejabcIcaOiabicdaWiabcMcaPiabgIGiolabdAfawXWaaSbaaKqzZdqaaiabicdaWaqabaGaeiilaWIaemyCaehddaqhaaqcLnpabaGaeGimaadabaGaemiraqeaaiabg2da9iabgEHiQaaaaaa@D8E7@

Second, we have to add to the finalization phase (23) an option

α(t)=(β0(P(t))T00(g,t)β0(P(t))T01(g,t)β1(P(t))T10(g,t)β1(P(t))T11(g,t))qt=∗.
 MathType@MTEF@5@5@+=feaafiart1ev1aaatCvAUfKttLearuWrP9MDH5MBPbIqV92AaeXatLxBI9gBaebbnrfifHhDYfgasaacH8akY=wiFfYdH8Gipec8Eeeu0xXdbba9frFj0=OqFfea0dXdd9vqai=hGuQ8kuc9pgc9s8qqaq=dirpe0xb9q8qiLsFr0=vr0=vr0dc8meaabaqaciaacaGaaeqabaqabeGadaaakeaafaqabeqacaaabaacciGae8xSdeMaeiikaGIaemiDaqNaeiykaKIaeyypa0ZaaeWaaeaafaqabeGacaaabaGae8NSdi2aaSbaaSqaaiabicdaWaqabaGccqGGOaakcqqGqbaucqGGOaakcqWG0baDcqGGPaqkcqGGPaqkcqWGubavdaWgaaWcbaGaeGimaaJaeGimaadabeaakiabcIcaOiabdEgaNjabcYcaSiabdsha0jabcMcaPaqaaiab=j7aInaaBaaaleaacqaIWaamaeqaaOGaeiikaGIaeeiuaaLaeiikaGIaemiDaqNaeiykaKIaeiykaKIaemivaq1aaSbaaSqaaiabicdaWiabigdaXaqabaGccqGGOaakcqWGNbWzcqGGSaalcqWG0baDcqGGPaqkaeaacqWFYoGydaWgaaWcbaGaeGymaedabeaakiabcIcaOiabbcfaqjabcIcaOiabdsha0jabcMcaPiabcMcaPiabdsfaunaaBaaaleaacqaIXaqmcqaIWaamaeqaaOGaeiikaGIaem4zaCMaeiilaWIaemiDaqNaeiykaKcabaGae8NSdi2aaSbaaSqaaiabigdaXaqabaGccqGGOaakcqqGqbaucqGGOaakcqWG0baDcqGGPaqkcqGGPaqkcqWGubavdaWgaaWcbaGaeGymaeJaeGymaedabeaakiabcIcaOiabdEgaNjabcYcaSiabdsha0jabcMcaPaaaaiaawIcacaGLPaaaaeaacqWGXbqCdaWgaaWcbaGaemiDaqhabeaakiabg2da9iabgEHiQaaacqGGUaGlaaa@7F7C@

These inward-outward recursions are the phylogenetic equivalent of the backward-forward recursions known from hidden Markov models, and other versions of this method have been developed previously [47,48]. The version developed here can be shown to be the realization of the junction tree algorithm [49] on rooted bifurcating trees. The junction tree algorithm is a scheme to compute marginal probabilities of maximal cliques on graphs by means of belief propagation on a modified junction tree. Indeed, the matrix *α *computes marginal probabilities of pairs (*t*, P(*t*)), but such pairs are nothing but maximal cliques on rooted bifurcating trees.

#### Computing the coefficients *w*_*gpkk'*_

Here we show that the *γ*-recursion is sufficient to compute the coefficients *w*_*gpkk*'_. From the definition, wgpkk'=Pr⁡(ρpη=k,ρpθ=k'|ωp,Ξ0,Ψg0,Λ0)
 MathType@MTEF@5@5@+=feaafiart1ev1aaatCvAUfKttLearuWrP9MDH5MBPbIqV92AaeXatLxBI9gBaebbnrfifHhDYfgasaacH8akY=wiFfYdH8Gipec8Eeeu0xXdbba9frFj0=OqFfea0dXdd9vqai=hGuQ8kuc9pgc9s8qqaq=dirpe0xb9q8qiLsFr0=vr0=vr0dc8meaabaqaciaacaGaaeqabaqabeGadaaakeaacqWG3bWDdaWgaaWcbaGaem4zaCMaemiCaaNaem4AaSMaem4AaSMaei4jaCcabeaakiabg2da9iGbccfaqjabckhaYjabcIcaOGGaciab=f8aYnaaDaaaleaacqWGWbaCaeaacqWF3oaAaaGccqGH9aqpcqWGRbWAcqGGSaalcqWFbpGCdaqhaaWcbaGaemiCaahabaGae8hUdehaaOGaeyypa0Jaem4AaSMaei4jaCIaeiiFaWNae8xYdC3aaSbaaSqaaiabdchaWbqabaGccqGGSaalcqqHEoawdaahaaWcbeqaaiabicdaWaaakiabcYcaSiabfI6aznaaDaaaleaacqWGNbWzaeaacqaIWaamaaGccqGGSaalcqqHBoatdaahaaWcbeqaaiabicdaWaaakiabcMcaPaaa@5B6A@. Using the Bayes formula Pr(*x, y *| *z*) = Pr(*x, y, z*)/Σ_*x*, *y *_Pr(*x, y, z*), we can rewrite it as

wgpkk'=Pr⁡(ρpη=k,ρpθ=k',ωp|Ξ0,Ψg0,Λ0)∑h,h'Pr⁡(ρpη=h,ρpθ=h',ωp|Ξ0,Ψg0,Λ0)==Pr⁡(ρpη=k|Ξ0,Ψg0,Λ0)⋅Pr⁡(ρpθ=k'|Ξ0,Ψg0,Λ0)⋅Pr⁡(ωp|Ξ0,Ψgkk'0)∑h,h'Pr⁡(ρpη=h|Ξ0,Ψg0,Λ0)⋅Pr⁡(ρpθ=h'|Ξ0,Ψg0,Λ0)⋅Pr⁡(ωp|Ξ0,Ψghh'0).
 MathType@MTEF@5@5@+=feaafiart1ev1aaatCvAUfKttLearuWrP9MDH5MBPbIqV92AaeXatLxBI9gBaebbnrfifHhDYfgasaacH8akY=wiFfYdH8Gipec8Eeeu0xXdbba9frFj0=OqFfea0dXdd9vqai=hGuQ8kuc9pgc9s8qqaq=dirpe0xb9q8qiLsFr0=vr0=vr0dc8meaabaqaciaacaGaaeqabaqabeGadaaakqaabeqaaiabdEha3naaBaaaleaacqWGNbWzcqWGWbaCcqWGRbWAcqWGRbWAcqGGNaWjaeqaaOGaeyypa0ZaaSaaaeaacyGGqbaucqGGYbGCcqGGOaakiiGacqWFbpGCdaqhaaWcbaGaemiCaahabaGae83TdGgaaOGaeyypa0Jaem4AaSMaeiilaWIae8xWdi3aa0baaSqaaiabdchaWbqaaiab=H7aXbaakiabg2da9iabdUgaRjabcEcaNiabcYcaSiab=L8a3naaBaaaleaacqWGWbaCaeqaaOGaeiiFaWNaeuONdG1aaWbaaSqabeaacqaIWaamaaGccqGGSaalcqqHOoqwdaqhaaWcbaGaem4zaCgabaGaeGimaadaaOGaeiilaWIaeu4MdW0aaWbaaSqabeaacqaIWaamaaGccqGGPaqkaeaadaaeqaqaaiGbccfaqjabckhaYjabcIcaOiab=f8aYnaaDaaaleaacqWGWbaCaeaacqWF3oaAaaGccqGH9aqpcqWGObaAcqGGSaalcqWFbpGCdaqhaaWcbaGaemiCaahabaGae8hUdehaaOGaeyypa0JaemiAaGMaei4jaCIaeiilaWIae8xYdC3aaSbaaSqaaiabdchaWbqabaGccqGG8baFcqqHEoawdaahaaWcbeqaaiabicdaWaaakiabcYcaSiabfI6aznaaDaaaleaacqWGNbWzaeaacqaIWaamaaGccqGGSaalcqqHBoatdaahaaWcbeqaaiabicdaWaaakiabcMcaPaWcbaGaemiAaGMaeiilaWIaemiAaGMaei4jaCcabeqdcqGHris5aaaakiabg2da9aqaaiaaxMaacqGH9aqpdaWcaaqaaiGbccfaqjabckhaYjabcIcaOiab=f8aYnaaDaaaleaacqWGWbaCaeaacqWF3oaAaaGccqGH9aqpcqWGRbWAcqGG8baFcqqHEoawdaahaaWcbeqaaiabicdaWaaakiabcYcaSiabfI6aznaaDaaaleaacqWGNbWzaeaacqaIWaamaaGccqGGSaalcqqHBoatdaahaaWcbeqaaiabicdaWaaakiabcMcaPiabgwSixlGbccfaqjabckhaYjabcIcaOiab=f8aYnaaDaaaleaacqWGWbaCaeaacqWF4oqCaaGccqGH9aqpcqWGRbWAcqGGNaWjcqGG8baFcqqHEoawdaahaaWcbeqaaiabicdaWaaakiabcYcaSiabfI6aznaaDaaaleaacqWGNbWzaeaacqaIWaamaaGccqGGSaalcqqHBoatdaahaaWcbeqaaiabicdaWaaakiabcMcaPiabgwSixlGbccfaqjabckhaYjabcIcaOiab=L8a3naaBaaaleaacqWGWbaCaeqaaOGaeiiFaWNaeuONdG1aaWbaaSqabeaacqaIWaamaaGccqGGSaalcqqHOoqwdaqhaaWcbaGaem4zaCMaem4AaSMaem4AaSMaei4jaCcabaGaeGimaadaaOGaeiykaKcabaWaaabeaeaacyGGqbaucqGGYbGCcqGGOaakcqWFbpGCdaqhaaWcbaGaemiCaahabaGae83TdGgaaOGaeyypa0JaemiAaGMaeiiFaWNaeuONdG1aaWbaaSqabeaacqaIWaamaaGccqGGSaalcqqHOoqwdaqhaaWcbaGaem4zaCgabaGaeGimaadaaOGaeiilaWIaeu4MdW0aaWbaaSqabeaacqaIWaamaaGccqGGPaqkcqGHflY1cyGGqbaucqGGYbGCcqGGOaakcqWFbpGCdaqhaaWcbaGaemiCaahabaGae8hUdehaaOGaeyypa0JaemiAaGMaei4jaCIaeiiFaWNaeuONdG1aaWbaaSqabeaacqaIWaamaaGccqGGSaalcqqHOoqwdaqhaaWcbaGaem4zaCgabaGaeGimaadaaOGaeiilaWIaeu4MdW0aaWbaaSqabeaacqaIWaamaaGccqGGPaqkcqGHflY1cyGGqbaucqGGYbGCcqGGOaakcqWFjpWDdaWgaaWcbaGaemiCaahabeaakiabcYha8jabf65aynaaCaaaleqabaGaeGimaadaaOGaeiilaWIaeuiQdK1aa0baaSqaaiabdEgaNjabdIgaOjabdIgaOjabcEcaNaqaaiabicdaWaaakiabcMcaPaWcbaGaemiAaGMaeiilaWIaemiAaGMaei4jaCcabeqdcqGHris5aaaakiabc6caUaaaaa@25DE@

But Pr⁡(ρpη=k|Ξ0,Ψg0,Λ0)
 MathType@MTEF@5@5@+=feaafiart1ev1aaatCvAUfKttLearuWrP9MDH5MBPbIqV92AaeXatLxBI9gBaebbnrfifHhDYfgasaacH8akY=wiFfYdH8Gipec8Eeeu0xXdbba9frFj0=OqFfea0dXdd9vqai=hGuQ8kuc9pgc9s8qqaq=dirpe0xb9q8qiLsFr0=vr0=vr0dc8meaabaqaciaacaGaaeqabaqabeGadaaakeaacyGGqbaucqGGYbGCcqGGOaakiiGacqWFbpGCdaqhaaWcbaGaemiCaahabaGae83TdGgaaOGaeyypa0Jaem4AaSMaeiiFaWNaeuONdG1aaWbaaSqabeaacqaIWaamaaGccqGGSaalcqqHOoqwdaqhaaWcbaGaem4zaCgabaGaeGimaadaaOGaeiilaWIaeu4MdW0aaWbaaSqabeaacqaIWaamaaGccqGGPaqkaaa@44F5@ is the current estimate of the probability of the gain rate variable to have the value rkη
 MathType@MTEF@5@5@+=feaafiart1ev1aaatCvAUfKttLearuWrP9MDH5MBPbIqV92AaeXatLxBI9gBaebbnrfifHhDYfgasaacH8akY=wiFfYdH8Gipec8Eeeu0xXdbba9frFj0=OqFfea0dXdd9vqai=hGuQ8kuc9pgc9s8qqaq=dirpe0xb9q8qiLsFr0=vr0=vr0dc8meaabaqaciaacaGaaeqabaqabeGadaaakeaacqWGYbGCdaqhaaWcbaGaem4AaSgabaacciGae83TdGgaaaaa@3158@, namely (fkη)0
 MathType@MTEF@5@5@+=feaafiart1ev1aaatCvAUfKttLearuWrP9MDH5MBPbIqV92AaeXatLxBI9gBaebbnrfifHhDYfgasaacH8akY=wiFfYdH8Gipec8Eeeu0xXdbba9frFj0=OqFfea0dXdd9vqai=hGuQ8kuc9pgc9s8qqaq=dirpe0xb9q8qiLsFr0=vr0=vr0dc8meaabaqaciaacaGaaeqabaqabeGadaaakeaacqGGOaakcqWGMbGzdaqhaaWcbaGaem4AaSgabaacciGae83TdGgaaOGaeiykaKYaaWbaaSqabeaacqaIWaamaaaaaa@3417@. Similarly, Pr⁡(ρpθ=k'|Ξ0,Ψg0,Λ0)
 MathType@MTEF@5@5@+=feaafiart1ev1aaatCvAUfKttLearuWrP9MDH5MBPbIqV92AaeXatLxBI9gBaebbnrfifHhDYfgasaacH8akY=wiFfYdH8Gipec8Eeeu0xXdbba9frFj0=OqFfea0dXdd9vqai=hGuQ8kuc9pgc9s8qqaq=dirpe0xb9q8qiLsFr0=vr0=vr0dc8meaabaqaciaacaGaaeqabaqabeGadaaakeaacyGGqbaucqGGYbGCcqGGOaakiiGacqWFbpGCdaqhaaWcbaGaemiCaahabaGae8hUdehaaOGaeyypa0Jaem4AaSMaei4jaCIaeiiFaWNaeuONdG1aaWbaaSqabeaacqaIWaamaaGccqGGSaalcqqHOoqwdaqhaaWcbaGaem4zaCgabaGaeGimaadaaOGaeiilaWIaeu4MdW0aaWbaaSqabeaacqaIWaamaaGccqGGPaqkaaa@45D5@ is just (fk'θ)0
 MathType@MTEF@5@5@+=feaafiart1ev1aaatCvAUfKttLearuWrP9MDH5MBPbIqV92AaeXatLxBI9gBaebbnrfifHhDYfgasaacH8akY=wiFfYdH8Gipec8Eeeu0xXdbba9frFj0=OqFfea0dXdd9vqai=hGuQ8kuc9pgc9s8qqaq=dirpe0xb9q8qiLsFr0=vr0=vr0dc8meaabaqaciaacaGaaeqabaqabeGadaaakeaacqGGOaakcqWGMbGzdaqhaaWcbaGaem4AaSMaei4jaCcabaacciGae8hUdehaaOGaeiykaKYaaWbaaSqabeaacqaIWaamaaaaaa@34F7@. Therefore, the expression for the coefficients *w*_*gpkk*' _reduces to

wgpkk'=(fkη)0(fk'θ)0Pr⁡(ωp|Ξ0,Ψgkk'0)∑h,h'(fhη)0(fh'θ)0Pr⁡(ωp|Ξ0,Ψghh'0).
 MathType@MTEF@5@5@+=feaafiart1ev1aaatCvAUfKttLearuWrP9MDH5MBPbIqV92AaeXatLxBI9gBaebbnrfifHhDYfgasaacH8akY=wiFfYdH8Gipec8Eeeu0xXdbba9frFj0=OqFfea0dXdd9vqai=hGuQ8kuc9pgc9s8qqaq=dirpe0xb9q8qiLsFr0=vr0=vr0dc8meaabaqaciaacaGaaeqabaqabeGadaaakeaacqWG3bWDdaWgaaWcbaGaem4zaCMaemiCaaNaem4AaSMaem4AaSMaei4jaCcabeaakiabg2da9maalaaabaGaeiikaGIaemOzay2aa0baaSqaaiabdUgaRbqaaGGaciab=D7aObaakiabcMcaPmaaCaaaleqabaGaeGimaadaaOGaeiikaGIaemOzay2aa0baaSqaaiabdUgaRjabcEcaNaqaaiab=H7aXbaakiabcMcaPmaaCaaaleqabaGaeGimaadaaOGagiiuaaLaeiOCaiNaeiikaGIae8xYdC3aaSbaaSqaaiabdchaWbqabaGccqGG8baFcqqHEoawdaahaaWcbeqaaiabicdaWaaakiabcYcaSiabfI6aznaaDaaaleaacqWGNbWzcqWGRbWAcqWGRbWAcqGGNaWjaeaacqaIWaamaaGccqGGPaqkaeaadaaeqaqaaiabcIcaOiabdAgaMnaaDaaaleaacqWGObaAaeaacqWF3oaAaaGccqGGPaqkdaahaaWcbeqaaiabicdaWaaakiabcIcaOiabdAgaMnaaDaaaleaacqWGObaAcqGGNaWjaeaacqWF4oqCaaGccqGGPaqkdaahaaWcbeqaaiabicdaWaaakiGbccfaqjabckhaYjabcIcaOiab=L8a3naaBaaaleaacqWGWbaCaeqaaOGaeiiFaWNaeuONdG1aaWbaaSqabeaacqaIWaamaaGccqGGSaalcqqHOoqwdaqhaaWcbaGaem4zaCMaemiAaGMaemiAaGMaei4jaCcabaGaeGimaadaaOGaeiykaKcaleaacqWGObaAcqGGSaalcqWGObaAcqGGNaWjaeqaniabggHiLdaaaOGaeiOla4caaa@8502@

The function Pr⁡(ωp|Ξ0,Ψgkk'0)
 MathType@MTEF@5@5@+=feaafiart1ev1aaatCvAUfKttLearuWrP9MDH5MBPbIqV92AaeXatLxBI9gBaebbnrfifHhDYfgasaacH8akY=wiFfYdH8Gipec8Eeeu0xXdbba9frFj0=OqFfea0dXdd9vqai=hGuQ8kuc9pgc9s8qqaq=dirpe0xb9q8qiLsFr0=vr0=vr0dc8meaabaqaciaacaGaaeqabaqabeGadaaakeaacyGGqbaucqGGYbGCcqGGOaakiiGacqWFjpWDdaWgaaWcbaGaemiCaahabeaakiabcYha8jabf65aynaaCaaaleqabaGaeGimaadaaOGaeiilaWIaeuiQdK1aa0baaSqaaiabdEgaNjabdUgaRjabdUgaRjabcEcaNaqaaiabicdaWaaakiabcMcaPaaa@410F@ is the likelihood of observing pattern *ω*_*p *_for gain and loss rate variables rkη
 MathType@MTEF@5@5@+=feaafiart1ev1aaatCvAUfKttLearuWrP9MDH5MBPbIqV92AaeXatLxBI9gBaebbnrfifHhDYfgasaacH8akY=wiFfYdH8Gipec8Eeeu0xXdbba9frFj0=OqFfea0dXdd9vqai=hGuQ8kuc9pgc9s8qqaq=dirpe0xb9q8qiLsFr0=vr0=vr0dc8meaabaqaciaacaGaaeqabaqabeGadaaakeaacqWGYbGCdaqhaaWcbaGaem4AaSgabaacciGae83TdGgaaaaa@3158@ and rk'θ
 MathType@MTEF@5@5@+=feaafiart1ev1aaatCvAUfKttLearuWrP9MDH5MBPbIqV92AaeXatLxBI9gBaebbnrfifHhDYfgasaacH8akY=wiFfYdH8Gipec8Eeeu0xXdbba9frFj0=OqFfea0dXdd9vqai=hGuQ8kuc9pgc9s8qqaq=dirpe0xb9q8qiLsFr0=vr0=vr0dc8meaabaqaciaacaGaaeqabaqabeGadaaakeaacqWGYbGCdaqhaaWcbaGaem4AaSMaei4jaCcabaacciGae8hUdehaaaaa@3238@, respectively. This is readily computed upon completion of the *γ*-recursion, using (15).

#### Computing the coefficients *Q*_*gpkk*'_

Here we show that those coefficients require the *α*, *β*-recursion. By definition,

Qgpkk'=∑σPr⁡(σ|ωp,Ξ0,Ψgkk'0)⋅[log⁡fkη+log⁡fk'θ+log⁡Pr⁡(ωp,σ|Ξ,Ψgkk')].
 MathType@MTEF@5@5@+=feaafiart1ev1aaatCvAUfKttLearuWrP9MDH5MBPbIqV92AaeXatLxBI9gBaebbnrfifHhDYfgasaacH8akY=wiFfYdH8Gipec8Eeeu0xXdbba9frFj0=OqFfea0dXdd9vqai=hGuQ8kuc9pgc9s8qqaq=dirpe0xb9q8qiLsFr0=vr0=vr0dc8meaabaqaciaacaGaaeqabaqabeGadaaakeaacqWGrbqudaWgaaWcbaGaem4zaCMaemiCaaNaem4AaSMaem4AaSMaei4jaCcabeaakiabg2da9maaqafabaGagiiuaaLaeiOCaiNaeiikaGccciGae83WdmNaeiiFaWNae8xYdC3aaSbaaSqaaiabdchaWbqabaGccqGGSaalcqqHEoawdaahaaWcbeqaaiabicdaWaaakiabcYcaSiabfI6aznaaDaaaleaacqWGNbWzcqWGRbWAcqWGRbWAcqGGNaWjaeaacqaIWaamaaGccqGGPaqkcqGHflY1cqGGBbWwcyGGSbaBcqGGVbWBcqGGNbWzcqWGMbGzdaqhaaWcbaGaem4AaSgabaGae83TdGgaaOGaey4kaSIagiiBaWMaei4Ba8Maei4zaCMaemOzay2aa0baaSqaaiabdUgaRjabcEcaNaqaaiab=H7aXbaakiabgUcaRiGbcYgaSjabc+gaVjabcEgaNjGbccfaqjabckhaYjabcIcaOiab=L8a3naaBaaaleaacqWGWbaCaeqaaOGaeiilaWIae83WdmNaeiiFaWNaeuONdGLaeiilaWIaeuiQdK1aaSbaaSqaaiabdEgaNjabdUgaRjabdUgaRjabcEcaNaqabaGccqGGPaqkcqGGDbqxaSqaaiab=n8aZbqab0GaeyyeIuoakiabc6caUaaa@8304@

The probability Pr(*ω*_*p*_, *σ *| Ξ, Ψ_*gkk*'_) is the likelihood of a particular realization of the tree, thus from (1)

log⁡Pr⁡(ωp,σ|Ξ,Ψgkk')=∑i=01δ(q0,i)⋅log⁡πi+∑i,j=01∑t=1N−1δ(qt,j)δ(qtP,i)⋅log⁡Tij(g,t).
 MathType@MTEF@5@5@+=feaafiart1ev1aaatCvAUfKttLearuWrP9MDH5MBPbIqV92AaeXatLxBI9gBaebbnrfifHhDYfgasaacH8akY=wiFfYdH8Gipec8Eeeu0xXdbba9frFj0=OqFfea0dXdd9vqai=hGuQ8kuc9pgc9s8qqaq=dirpe0xb9q8qiLsFr0=vr0=vr0dc8meaabaqaciaacaGaaeqabaqabeGadaaakeaacyGGSbaBcqGGVbWBcqGGNbWzcyGGqbaucqGGYbGCcqGGOaakiiGacqWFjpWDdaWgaaWcbaGaemiCaahabeaakiabcYcaSiab=n8aZjabcYha8jabf65ayjabcYcaSiabfI6aznaaBaaaleaacqWGNbWzcqWGRbWAcqWGRbWAcqGGNaWjaeqaaOGaeiykaKIaeyypa0ZaaabCaeaacqWF0oazcqGGOaakcqWGXbqCdaWgaaWcbaGaeGimaadabeaakiabcYcaSiabdMgaPjabcMcaPiabgwSixlGbcYgaSjabc+gaVjabcEgaNjab=b8aWnaaBaaaleaacqWGPbqAaeqaaaqaaiabdMgaPjabg2da9iabicdaWaqaaiabigdaXaqdcqGHris5aOGaey4kaSYaaabCaeaadaaeWbqaaiab=r7aKjabcIcaOiabdghaXnaaBaaaleaacqWG0baDaeqaaOGaeiilaWIaemOAaOMaeiykaKIae8hTdqMaeiikaGIaemyCae3aa0baaSqaaiabdsha0bqaaiabdcfaqbaakiabcYcaSiabdMgaPjabcMcaPiabgwSixlGbcYgaSjabc+gaVjabcEgaNjabdsfaunaaBaaaleaacqWGPbqAcqWGQbGAaeqaaOGaeiikaGIaem4zaCMaeiilaWIaemiDaqNaeiykaKcaleaacqWG0baDcqGH9aqpcqaIXaqmaeaacqWGobGtcqGHsislcqaIXaqma0GaeyyeIuoaaSqaaiabdMgaPjabcYcaSiabdQgaQjabg2da9iabicdaWaqaaiabigdaXaqdcqGHris5aOGaeiOla4caaa@9484@

Here, *δ*(*a, b*) is the Kronecker delta function, which is 1 for *a *= *b *and 0 otherwise. Denote the expectation over Pr⁡(σ|ωp,Ξ0,Ψgkk'0)
 MathType@MTEF@5@5@+=feaafiart1ev1aaatCvAUfKttLearuWrP9MDH5MBPbIqV92AaeXatLxBI9gBaebbnrfifHhDYfgasaacH8akY=wiFfYdH8Gipec8Eeeu0xXdbba9frFj0=OqFfea0dXdd9vqai=hGuQ8kuc9pgc9s8qqaq=dirpe0xb9q8qiLsFr0=vr0=vr0dc8meaabaqaciaacaGaaeqabaqabeGadaaakeaacyGGqbaucqGGYbGCcqGGOaakiiGacqWFdpWCcqGG8baFcqWFjpWDdaWgaaWcbaGaemiCaahabeaakiabcYcaSiabf65aynaaCaaaleqabaGaeGimaadaaOGaeiilaWIaeuiQdK1aa0baaSqaaiabdEgaNjabdUgaRjabdUgaRjabcEcaNaqaaiabicdaWaaakiabcMcaPaaa@43AD@ by *E*_*σ*_. Applying it to (25), we get

Eσ[log⁡Pr⁡(ωp,σ|Ξ,Ψgkk')]=∑i=01log⁡πi⋅Eσ[δ(q0,i)]+∑i,j=01∑t=1N−1log⁡Tij(g,t)⋅Eσ[δ(qt,j)δ(qtP,i)].
 MathType@MTEF@5@5@+=feaafiart1ev1aaatCvAUfKttLearuWrP9MDH5MBPbIqV92AaeXatLxBI9gBaebbnrfifHhDYfgasaacH8akY=wiFfYdH8Gipec8Eeeu0xXdbba9frFj0=OqFfea0dXdd9vqai=hGuQ8kuc9pgc9s8qqaq=dirpe0xb9q8qiLsFr0=vr0=vr0dc8meaabaqaciaacaGaaeqabaqabeGadaaakeaajugibiabdweafTWaaSbaaOqaaGGacKqzGeGae83WdmhakeqaaKqzGeGaei4waSLagiiBaWMaei4Ba8Maei4zaCMagiiuaaLaeiOCaiNaeiikaGIae8xYdC3cdaWgaaGcbaqcLbsacqWGWbaCaOqabaqcLbsacqGGSaalcqWFdpWCcqGG8baFcqqHEoawcqGGSaalcqqHOoqwlmaaBaaakeaajugibiabdEgaNjabdUgaRjabdUgaRjabcEcaNaGcbeaajugibiabcMcaPiabc2faDjabg2da9OWaaabCaeaajugibiGbcYgaSjabc+gaVjabcEgaNjab=b8aWTWaaSbaaOqaaKqzGeGaemyAaKgakeqaaKqzGeGaeyyXICTaemyrau0cdaWgaaGcbaqcLbsacqWFdpWCaOqabaqcLbsacqGGBbWwcqWF0oazcqGGOaakcqWGXbqClmaaBaaajugibeaacqaIWaamaeqaaiabcYcaSiabdMgaPjabcMcaPiabc2faDbWcbaGaemyAaKMaeyypa0JaeGimaadabaGaeGymaedaniabggHiLdqcLbsacqGHRaWkkmaaqahabaWaaabCaeaacyGGSbaBcqGGVbWBcqGGNbWzcqWGubavdaWgaaWcbaGaemyAaKMaemOAaOgabeaakiabcIcaOiabdEgaNjabcYcaSiabdsha0jabcMcaPiabgwSixlabdweafnaaBaaaleaacqWFdpWCaeqaaOGaei4waSLae8hTdqMaeiikaGIaemyCae3aaSbaaSqaaiabdsha0bqabaGccqGGSaalcqWGQbGAcqGGPaqkcqWF0oazcqGGOaakcqWGXbqCdaqhaaWcbaGaemiDaqhabaGaemiuaafaaOGaeiilaWIaemyAaKMaeiykaKIaeiyxa0faleaacqWG0baDcqGH9aqpcqaIXaqmaeaacqWGobGtcqGHsislcqaIXaqma0GaeyyeIuoaaSqaaiabdMgaPjabcYcaSiabdQgaQjabg2da9iabicdaWaqaaiabigdaXaqdcqGHris5aOGaeiOla4caaa@AD32@

But Eσ[δ(q0,i)]=Pr⁡(q0=i|ωp,Ξ0,Ψgkk'0)=βi(0)
 MathType@MTEF@5@5@+=feaafiart1ev1aaatCvAUfKttLearuWrP9MDH5MBPbIqV92AaeXatLxBI9gBaebbnrfifHhDYfgasaacH8akY=wiFfYdH8Gipec8Eeeu0xXdbba9frFj0=OqFfea0dXdd9vqai=hGuQ8kuc9pgc9s8qqaq=dirpe0xb9q8qiLsFr0=vr0=vr0dc8meaabaqaciaacaGaaeqabaqabeGadaaakeaacqWGfbqrdaWgaaWcbaacciGae83WdmhabeaakiabcUfaBjab=r7aKjabcIcaOiabdghaXnaaBaaaleaacqaIWaamaeqaaOGaeiilaWIaemyAaKMaeiykaKIaeiyxa0Laeyypa0JagiiuaaLaeiOCaiNaeiikaGIaemyCae3aaSbaaSqaaiabicdaWaqabaGccqGH9aqpcqWGPbqAcqGG8baFcqWFjpWDdaWgaaWcbaGaemiCaahabeaakiabcYcaSiabf65aynaaCaaaleqabaGaeGimaadaaOGaeiilaWIaeuiQdK1aa0baaSqaaiabdEgaNjabdUgaRjabdUgaRjabcEcaNaqaaiabicdaWaaakiabcMcaPiabg2da9iab=j7aInaaBaaaleaacqWGPbqAaeqaaOGaeiikaGIaeGimaaJaeiykaKcaaa@5C5B@, and similarly Eσ[δ(qt,j)δ(qtP,i)]=αij(t)
 MathType@MTEF@5@5@+=feaafiart1ev1aaatCvAUfKttLearuWrP9MDH5MBPbIqV92AaeXatLxBI9gBaebbnrfifHhDYfgasaacH8akY=wiFfYdH8Gipec8Eeeu0xXdbba9frFj0=OqFfea0dXdd9vqai=hGuQ8kuc9pgc9s8qqaq=dirpe0xb9q8qiLsFr0=vr0=vr0dc8meaabaqaciaacaGaaeqabaqabeGadaaakeaacqWGfbqrdaWgaaWcbaacciGae83WdmhabeaakiabcUfaBjab=r7aKjabcIcaOiabdghaXnaaBaaaleaacqWG0baDaeqaaOGaeiilaWIaemOAaOMaeiykaKIae8hTdqMaeiikaGIaemyCae3aa0baaSqaaiabdsha0bqaaiabdcfaqbaakiabcYcaSiabdMgaPjabcMcaPiabc2faDjabg2da9iab=f7aHnaaBaaaleaacqWGPbqAcqWGQbGAaeqaaOGaeiikaGIaemiDaqNaeiykaKcaaa@4D5A@.

Hence, *Q*_*gpkk*' _is given by

Qgpkk'=∑σPr⁡(σ|ωp,Ξ0,Ψgkk'0)[log⁡fkη+log⁡fk'θ+log⁡Pr⁡(ωp,σ|Ξ,Ψgkk')]==log⁡fkη+log⁡fk'θ+∑i=01βi(0)log⁡πi+∑i,j=01∑t=1N−1αij(t)log⁡Tij(g,t).
 MathType@MTEF@5@5@+=feaafiart1ev1aaatCvAUfKttLearuWrP9MDH5MBPbIqV92AaeXatLxBI9gBaebbnrfifHhDYfgasaacH8akY=wiFfYdH8Gipec8Eeeu0xXdbba9frFj0=OqFfea0dXdd9vqai=hGuQ8kuc9pgc9s8qqaq=dirpe0xb9q8qiLsFr0=vr0=vr0dc8meaabaqaciaacaGaaeqabaqabeGadaaakqaabeqaaiabdgfarnaaBaaaleaacqWGNbWzcqWGWbaCcqWGRbWAcqWGRbWAcqGGNaWjaeqaaOGaeyypa0ZaaabuaeaacyGGqbaucqGGYbGCcqGGOaakiiGacqWFdpWCcqGG8baFcqWFjpWDdaWgaaWcbaGaemiCaahabeaakiabcYcaSiabf65aynaaCaaaleqabaGaeGimaadaaOGaeiilaWIaeuiQdK1aa0baaSqaaiabdEgaNjabdUgaRjabdUgaRjabcEcaNaqaaiabicdaWaaakiabcMcaPiabcUfaBjGbcYgaSjabc+gaVjabcEgaNjabdAgaMnaaDaaaleaacqWGRbWAaeaacqWF3oaAaaGccqGHRaWkcyGGSbaBcqGGVbWBcqGGNbWzcqWGMbGzdaqhaaWcbaGaem4AaSMaei4jaCcabaGae8hUdehaaOGaey4kaSIagiiBaWMaei4Ba8Maei4zaCMagiiuaaLaeiOCaiNaeiikaGIae8xYdC3aaSbaaSqaaiabdchaWbqabaGccqGGSaalcqWFdpWCcqGG8baFcqqHEoawcqGGSaalcqqHOoqwdaWgaaWcbaGaem4zaCMaem4AaSMaem4AaSMaei4jaCcabeaakiabcMcaPiabc2faDbWcbaGae83WdmhabeqdcqGHris5aOGaeyypa0dabaGaaCzcaiabg2da9iGbcYgaSjabc+gaVjabcEgaNjabdAgaMnaaDaaaleaacqWGRbWAaeaacqWF3oaAaaGccqGHRaWkcyGGSbaBcqGGVbWBcqGGNbWzcqWGMbGzdaqhaaWcbaGaem4AaSMaei4jaCcabaGae8hUdehaaOGaey4kaSYaaabCaeaacqWFYoGydaWgaaWcbaGaemyAaKgabeaakiabcIcaOiabicdaWiabcMcaPiGbcYgaSjabc+gaVjabcEgaNjab=b8aWnaaBaaaleaacqWGPbqAaeqaaaqaaiabdMgaPjabg2da9iabicdaWaqaaiabigdaXaqdcqGHris5aOGaey4kaSYaaabCaeaadaaeWbqaaiab=f7aHnaaBaaaleaacqWGPbqAcqWGQbGAaeqaaOGaeiikaGIaemiDaqNaeiykaKIagiiBaWMaei4Ba8Maei4zaCMaemivaq1aaSbaaSqaaiabdMgaPjabdQgaQbqabaGccqGGOaakcqWGNbWzcqGGSaalcqWG0baDcqGGPaqkcqGGUaGlaSqaaiabdsha0jabg2da9iabigdaXaqaaiabd6eaojabgkHiTiabigdaXaqdcqGHris5aaWcbaGaemyAaKMaeiilaWIaemOAaOMaeyypa0JaeGimaadabaGaeGymaedaniabggHiLdaaaaa@D2AA@

### The M-step

Substituting (26) in (11), we obtain an explicit form of the function whose maximization guarantees stepping up-hill in the likelihood landscape,

Q=∑g=1G∑p=1Ω∑k=1Kη∑k'=1Kθngpwgpkk'(log⁡fkη+log⁡fk'θ)++∑g=1G∑p=1Ω∑k=1Kη∑k'=1Kθngpwgpkk'[β0gpkk'(0)log⁡π0+β1gpkk'(0)log⁡π1]++∑g=1G∑p=1Ω∑k=1Kη∑k'=1Kθ∑t=1N−1ngpwgpkk'α00gpkk'(t)log⁡[1−ξt(1−e−ηgkΔt)]++∑g=1G∑p=1Ω∑k=1Kη∑k'=1Kθ∑t=1N−1ngpwgpkk'α01gpkk'(t)[log⁡ξt+log⁡(1−e−ηgkΔt)]++∑g=1G∑p=1Ω∑k=1Kη∑k'=1Kθ∑t=1N−1ngpwgpkk'α10gpkk'(t)log⁡[1−(1−φt)e−θgk'Δt]++∑g=1G∑p=1Ω∑k=1Kη∑k'=1Kθ∑t=1N−1ngpwgpkk'α11gpkk'(t)[log⁡(1−φt)−θgk'Δt].
 MathType@MTEF@5@5@+=feaafiart1ev1aaatCvAUfKttLearuWrP9MDH5MBPbIqV92AaeXatLxBI9gBaebbnrfifHhDYfgasaacH8akY=wiFfYdH8Gipec8Eeeu0xXdbba9frFj0=OqFfea0dXdd9vqai=hGuQ8kuc9pgc9s8qqaq=dirpe0xb9q8qiLsFr0=vr0=vr0dc8meaabaqaciaacaGaaeqabaqabeGadaaakeaafaqadeGbbaaaaeaacqWGrbqucqGH9aqpdaaeWbqaamaaqahabaWaaabCaeaadaaeWbqaaiabd6gaUnaaBaaaleaacqWGNbWzcqWGWbaCaeqaaOGaem4DaC3aaSbaaSqaaiabdEgaNjabdchaWjabdUgaRjabdUgaRjabcEcaNaqabaGccqGGOaakcyGGSbaBcqGGVbWBcqGGNbWzcqWGMbGzdaqhaaWcbaGaem4AaSgabaacciGae83TdGgaaOGaey4kaSIagiiBaWMaei4Ba8Maei4zaCMaemOzay2aa0baaSqaaiabdUgaRjabcEcaNaqaaiab=H7aXbaakiabcMcaPaWcbaGaem4AaSMaei4jaCIaeyypa0JaeGymaedabaGaem4saS0aaSbaaWqaaiab=H7aXbqabaaaniabggHiLdaaleaacqWGRbWAcqGH9aqpcqaIXaqmaeaacqWGlbWsdaWgaaadbaGae83TdGgabeaaa0GaeyyeIuoaaSqaaiabdchaWjabg2da9iabigdaXaqaaiabfM6axbqdcqGHris5aaWcbaGaem4zaCMaeyypa0JaeGymaedabaGaem4raCeaniabggHiLdGccqGHRaWkaeaacqGHRaWkdaaeWbqaamaaqahabaWaaabCaeaadaaeWbqaaiabd6gaUnaaBaaaleaacqWGNbWzcqWGWbaCaeqaaOGaem4DaC3aaSbaaSqaaiabdEgaNjabdchaWjabdUgaRjabdUgaRjabcEcaNaqabaGccqGGBbWwcqWFYoGydaqhaaWcbaGaeGimaadabaGaem4zaCMaemiCaaNaem4AaSMaem4AaSMaei4jaCcaaOGaeiikaGIaeGimaaJaeiykaKIagiiBaWMaei4Ba8Maei4zaCMae8hWda3aaSbaaSqaaiabicdaWaqabaGccqGHRaWkcqWFYoGydaqhaaWcbaGaeGymaedabaGaem4zaCMaemiCaaNaem4AaSMaem4AaSMaei4jaCcaaOGaeiikaGIaeGimaaJaeiykaKIagiiBaWMaei4Ba8Maei4zaCMae8hWda3aaSbaaSqaaiabigdaXaqabaGccqGGDbqxaSqaaiabdUgaRjabcEcaNiabg2da9iabigdaXaqaaiabdUealnaaBaaameaacqWF4oqCaeqaaaqdcqGHris5aaWcbaGaem4AaSMaeyypa0JaeGymaedabaGaem4saS0aaSbaaWqaaiab=D7aObqabaaaniabggHiLdaaleaacqWGWbaCcqGH9aqpcqaIXaqmaeaacqqHPoWva0GaeyyeIuoaaSqaaiabdEgaNjabg2da9iabigdaXaqaaiabdEeahbqdcqGHris5aOGaey4kaScabaGaey4kaSYaaabCaeaadaaeWbqaamaaqahabaWaaabCaeaadaaeWbqaaiabd6gaUnaaBaaaleaacqWGNbWzcqWGWbaCaeqaaOGaem4DaC3aaSbaaSqaaiabdEgaNjabdchaWjabdUgaRjabdUgaRjabcEcaNaqabaaabaGaemiDaqNaeyypa0JaeGymaedabaGaemOta4KaeyOeI0IaeGymaedaniabggHiLdGccqWFXoqydaqhaaWcbaGaeGimaaJaeGimaadabaGaem4zaCMaemiCaaNaem4AaSMaem4AaSMaei4jaCcaaOGaeiikaGIaemiDaqNaeiykaKIagiiBaWMaei4Ba8Maei4zaCMaei4waSLaeGymaeJaeyOeI0Iae8NVdG3aaSbaaSqaaiabdsha0bqabaGccqGGOaakcqaIXaqmcqGHsislcqWGLbqzdaahaaWcbeqaaiabgkHiTiab=D7aOnaaBaaameaacqWGNbWzcqWGRbWAaeqaaSGaeuiLdq0aaSbaaWqaaiabdsha0bqabaaaaOGaeiykaKIaeiyxa0faleaacqWGRbWAcqGGNaWjcqGH9aqpcqaIXaqmaeaacqWGlbWsdaWgaaadbaGae8hUdehabeaaa0GaeyyeIuoaaSqaaiabdUgaRjabg2da9iabigdaXaqaaiabdUealnaaBaaameaacqWF3oaAaeqaaaqdcqGHris5aaWcbaGaemiCaaNaeyypa0JaeGymaedabaGaeuyQdCfaniabggHiLdaaleaacqWGNbWzcqGH9aqpcqaIXaqmaeaacqWGhbWra0GaeyyeIuoakiabgUcaRaqaaiabgUcaRmaaqahabaWaaabCaeaadaaeWbqaamaaqahabaWaaabCaeaacqWGUbGBdaWgaaWcbaGaem4zaCMaemiCaahabeaakiabdEha3naaBaaaleaacqWGNbWzcqWGWbaCcqWGRbWAcqWGRbWAcqGGNaWjaeqaaaqaaiabdsha0jabg2da9iabigdaXaqaaiabd6eaojabgkHiTiabigdaXaqdcqGHris5aOGae8xSde2aa0baaSqaaiabicdaWiabigdaXaqaaiabdEgaNjabdchaWjabdUgaRjabdUgaRjabcEcaNaaakiabcIcaOiabdsha0jabcMcaPiabcUfaBjGbcYgaSjabc+gaVjabcEgaNjab=57a4naaBaaaleaacqWG0baDaeqaaOGaey4kaSIagiiBaWMaei4Ba8Maei4zaCMaeiikaGIaeGymaeJaeyOeI0Iaemyzau2aaWbaaSqabeaacqGHsislcqWF3oaAdaWgaaadbaGaem4zaCMaem4AaSgabeaaliabfs5aenaaBaaameaacqWG0baDaeqaaaaakiabcMcaPiabc2faDbWcbaGaem4AaSMaei4jaCIaeyypa0JaeGymaedabaGaem4saS0aaSbaaWqaaiab=H7aXbqabaaaniabggHiLdaaleaacqWGRbWAcqGH9aqpcqaIXaqmaeaacqWGlbWsdaWgaaadbaGae83TdGgabeaaa0GaeyyeIuoaaSqaaiabdchaWjabg2da9iabigdaXaqaaiabfM6axbqdcqGHris5aaWcbaGaem4zaCMaeyypa0JaeGymaedabaGaem4raCeaniabggHiLdGccqGHRaWkaeaacqGHRaWkdaaeWbqaamaaqahabaWaaabCaeaadaaeWbqaamaaqahabaGaemOBa42aaSbaaSqaaiabdEgaNjabdchaWbqabaGccqWG3bWDdaWgaaWcbaGaem4zaCMaemiCaaNaem4AaSMaem4AaSMaei4jaCcabeaaaeaacqWG0baDcqGH9aqpcqaIXaqmaeaacqWGobGtcqGHsislcqaIXaqma0GaeyyeIuoakiab=f7aHnaaDaaaleaacqaIXaqmcqaIWaamaeaacqWGNbWzcqWGWbaCcqWGRbWAcqWGRbWAcqGGNaWjaaGccqGGOaakcqWG0baDcqGGPaqkcyGGSbaBcqGGVbWBcqGGNbWzcqGGBbWwcqaIXaqmcqGHsislcqGGOaakcqaIXaqmcqGHsislcqWFgpGzdaWgaaWcbaGaemiDaqhabeaakiabcMcaPiabdwgaLnaaCaaaleqabaGaeyOeI0Iae8hUde3aaSbaaWqaaiabdEgaNjabdUgaRjabcEcaNaqabaWccqqHuoardaWgaaadbaGaemiDaqhabeaaaaGccqGGDbqxaSqaaiabdUgaRjabcEcaNiabg2da9iabigdaXaqaaiabdUealnaaBaaameaacqWF4oqCaeqaaaqdcqGHris5aaWcbaGaem4AaSMaeyypa0JaeGymaedabaGaem4saS0aaSbaaWqaaiab=D7aObqabaaaniabggHiLdaaleaacqWGWbaCcqGH9aqpcqaIXaqmaeaacqqHPoWva0GaeyyeIuoaaSqaaiabdEgaNjabg2da9iabigdaXaqaaiabdEeahbqdcqGHris5aOGaey4kaScabaGaey4kaSYaaabCaeaadaaeWbqaamaaqahabaWaaabCaeaadaaeWbqaaiabd6gaUnaaBaaaleaacqWGNbWzcqWGWbaCaeqaaOGaem4DaC3aaSbaaSqaaiabdEgaNjabdchaWjabdUgaRjabdUgaRjabcEcaNaqabaaabaGaemiDaqNaeyypa0JaeGymaedabaGaemOta4KaeyOeI0IaeGymaedaniabggHiLdGccqWFXoqydaqhaaWcbaGaeGymaeJaeGymaedabaGaem4zaCMaemiCaaNaem4AaSMaem4AaSMaei4jaCcaaOGaeiikaGIaemiDaqNaeiykaKIaei4waSLagiiBaWMaei4Ba8Maei4zaCMaeiikaGIaeGymaeJaeyOeI0Iae8NXdy2aaSbaaSqaaiabdsha0bqabaGccqGGPaqkcqGHsislcqWF4oqCdaWgaaWcbaGaem4zaCMaem4AaSMaei4jaCcabeaakiabfs5aenaaBaaaleaacqWG0baDaeqaaOGaeiyxa0faleaacqWGRbWAcqGGNaWjcqGH9aqpcqaIXaqmaeaacqWGlbWsdaWgaaadbaGae8hUdehabeaaa0GaeyyeIuoaaSqaaiabdUgaRjabg2da9iabigdaXaqaaiabdUealnaaBaaameaacqWF3oaAaeqaaaqdcqGHris5aaWcbaGaemiCaaNaeyypa0JaeGymaedabaGaeuyQdCfaniabggHiLdaaleaacqWGNbWzcqGH9aqpcqaIXaqmaeaacqWGhbWra0GaeyyeIuoakiabc6caUaaaaaa@3DED@

Actually, any increase in *Q *is sufficient to guarantee an increase in the likelihood, suggesting that the precise maximization of *Q *is not particularly important. Therefore, we speed up the computations by performing low-tolerance maximization with respect to each of the parameters individually. Except for the parameters *λ*_*η *_and *λ*_*θ*_, it is easy to differentiate *Q *twice with respect to any parameter. This lends itself to using simple zero-finding algorithms of which we chose the Newton-Raphson algorithm [50].

Maximizing *Q *with respect to the shape parameters *λ*_*η *_and *λ*_*θ *_is more involved, as *Q *depends on these parameters only through the discrete approximation of the rate variability distributions (3). In our implementation, we used Yang's quantile method [34] to compute the discrete levels of the gamma distributions such that each level has equal probability. Formally, f1η=ν,fkη=(1−ν)/(Kη−1)
 MathType@MTEF@5@5@+=feaafiart1ev1aaatCvAUfKttLearuWrP9MDH5MBPbIqV92AaeXatLxBI9gBaebbnrfifHhDYfgasaacH8akY=wiFfYdH8Gipec8Eeeu0xXdbba9frFj0=OqFfea0dXdd9vqai=hGuQ8kuc9pgc9s8qqaq=dirpe0xb9q8qiLsFr0=vr0=vr0dc8meaabaqaciaacaGaaeqabaqabeGadaaakeaacqWGMbGzdaqhaaWcbaGaeGymaedabaacciGae83TdGgaaOGaeyypa0Jae8xVd4MaeiilaWIaemOzay2aa0baaSqaaiabdUgaRbqaaiab=D7aObaakiabg2da9iabcIcaOiabigdaXiabgkHiTiab=17aUjabcMcaPiabc+caViabcIcaOiabdUealnaaBaaaleaacqWF3oaAaeqaaOGaeyOeI0IaeGymaeJaeiykaKcaaa@46BF@ for *k *= 2, ..., *K*_*η *_and fkθ=1/Kθ
 MathType@MTEF@5@5@+=feaafiart1ev1aaatCvAUfKttLearuWrP9MDH5MBPbIqV92AaeXatLxBI9gBaebbnrfifHhDYfgasaacH8akY=wiFfYdH8Gipec8Eeeu0xXdbba9frFj0=OqFfea0dXdd9vqai=hGuQ8kuc9pgc9s8qqaq=dirpe0xb9q8qiLsFr0=vr0=vr0dc8meaabaqaciaacaGaaeqabaqabeGadaaakeaacqWGMbGzdaqhaaWcbaGaem4AaSgabaacciGae8hUdehaaOGaeyypa0JaeGymaeJaei4la8Iaem4saS0aaSbaaSqaaiab=H7aXbqabaaaaa@372C@ for *k *= 1, ..., *K*_*θ*_. To perform the maximization in this case, we used Brent's maximization algorithm that does not require derivatives [50].

#### Reconstruction of Ancestral States and Events

Given the *α*, *β*, *γ*-recursions on the tree with the final model parameters, it is straightforward to reconstruct the history of intron evolution, and to assign gains and losses to specific branches. The number of introns in an internal node *t *for a gene *g*, assuming gain rate variable rkη
 MathType@MTEF@5@5@+=feaafiart1ev1aaatCvAUfKttLearuWrP9MDH5MBPbIqV92AaeXatLxBI9gBaebbnrfifHhDYfgasaacH8akY=wiFfYdH8Gipec8Eeeu0xXdbba9frFj0=OqFfea0dXdd9vqai=hGuQ8kuc9pgc9s8qqaq=dirpe0xb9q8qiLsFr0=vr0=vr0dc8meaabaqaciaacaGaaeqabaqabeGadaaakeaacqWGYbGCdaqhaaWcbaGaem4AaSgabaacciGae83TdGgaaaaa@3158@ and loss rate variable rk'θ
 MathType@MTEF@5@5@+=feaafiart1ev1aaatCvAUfKttLearuWrP9MDH5MBPbIqV92AaeXatLxBI9gBaebbnrfifHhDYfgasaacH8akY=wiFfYdH8Gipec8Eeeu0xXdbba9frFj0=OqFfea0dXdd9vqai=hGuQ8kuc9pgc9s8qqaq=dirpe0xb9q8qiLsFr0=vr0=vr0dc8meaabaqaciaacaGaaeqabaqabeGadaaakeaacqWGYbGCdaqhaaWcbaGaem4AaSMaei4jaCcabaacciGae8hUdehaaaaa@3238@, given that the observed pattern is *ω*_*p*_, is ngpωgpkk'β1gpkk'(t)
 MathType@MTEF@5@5@+=feaafiart1ev1aaatCvAUfKttLearuWrP9MDH5MBPbIqV92AaeXatLxBI9gBaebbnrfifHhDYfgasaacH8akY=wiFfYdH8Gipec8Eeeu0xXdbba9frFj0=OqFfea0dXdd9vqai=hGuQ8kuc9pgc9s8qqaq=dirpe0xb9q8qiLsFr0=vr0=vr0dc8meaabaqaciaacaGaaeqabaqabeGadaaakeaacqWGUbGBdaWgaaWcbaGaem4zaCMaemiCaahabeaaiiGakiab=L8a3naaBaaaleaacqWGNbWzcqWGWbaCcqWGRbWAcqWGRbWAcqGGNaWjaeqaaOGae8NSdi2aa0baaSqaaiabigdaXaqaaiabdEgaNjabdchaWjabdUgaRjabdUgaRjabcEcaNaaakiabcIcaOiabdsha0jabcMcaPaaa@459F@. Similarly, the number of loss events along the branch *t *is ngpωgpkk'α10gpkk'(t)
 MathType@MTEF@5@5@+=feaafiart1ev1aaatCvAUfKttLearuWrP9MDH5MBPbIqV92AaeXatLxBI9gBaebbnrfifHhDYfgasaacH8akY=wiFfYdH8Gipec8Eeeu0xXdbba9frFj0=OqFfea0dXdd9vqai=hGuQ8kuc9pgc9s8qqaq=dirpe0xb9q8qiLsFr0=vr0=vr0dc8meaabaqaciaacaGaaeqabaqabeGadaaakeaacqWGUbGBdaWgaaWcbaGaem4zaCMaemiCaahabeaaiiGakiab=L8a3naaBaaaleaacqWGNbWzcqWGWbaCcqWGRbWAcqWGRbWAcqGGNaWjaeqaaOGae8xSde2aa0baaSqaaiabigdaXiabicdaWaqaaiabdEgaNjabdchaWjabdUgaRjabdUgaRjabcEcaNaaakiabcIcaOiabdsha0jabcMcaPaaa@468B@, and the number of gain events along this branch is ngpωgpkk'α01gpkk'(t)
 MathType@MTEF@5@5@+=feaafiart1ev1aaatCvAUfKttLearuWrP9MDH5MBPbIqV92AaeXatLxBI9gBaebbnrfifHhDYfgasaacH8akY=wiFfYdH8Gipec8Eeeu0xXdbba9frFj0=OqFfea0dXdd9vqai=hGuQ8kuc9pgc9s8qqaq=dirpe0xb9q8qiLsFr0=vr0=vr0dc8meaabaqaciaacaGaaeqabaqabeGadaaakeaacqWGUbGBdaWgaaWcbaGaem4zaCMaemiCaahabeaaiiGakiab=L8a3naaBaaaleaacqWGNbWzcqWGWbaCcqWGRbWAcqWGRbWAcqGGNaWjaeqaaOGae8xSde2aa0baaSqaaiabicdaWiabigdaXaqaaiabdEgaNjabdchaWjabdUgaRjabdUgaRjabcEcaNaaakiabcIcaOiabdsha0jabcMcaPaaa@468B@.

#### Confidence Intervals

In order to obtain confidence intervals on the model parameters, we used the profile likelihood technique. In brief, if ϑ is one parameter in the model, and Θ¯
 MathType@MTEF@5@5@+=feaafiart1ev1aaatCvAUfKttLearuWrP9MDH5MBPbIqV92AaeXatLxBI9gBaebbnrfifHhDYfgasaacH8akY=wiFfYdH8Gipec8Eeeu0xXdbba9frFj0=OqFfea0dXdd9vqai=hGuQ8kuc9pgc9s8qqaq=dirpe0xb9q8qiLsFr0=vr0=vr0dc8meaabaqaciaacaGaaeqabaqabeGadaaakeaacuqHyoqugaqeaaaa@2E3B@ is the set of remaining parameters, then the profile likelihood of ϑ is defined as

L(ϑ)=max⁡Θ¯Pr⁡(ϑ,Θ¯).
 MathType@MTEF@5@5@+=feaafiart1ev1aaatCvAUfKttLearuWrP9MDH5MBPbIqV92AaeXatLxBI9gBaebbnrfifHhDYfgasaacH8akY=wiFfYdH8Gipec8Eeeu0xXdbba9frFj0=OqFfea0dXdd9vqai=hGuQ8kuc9pgc9s8qqaq=dirpe0xb9q8qiLsFr0=vr0=vr0dc8meaabaqaciaacaGaaeqabaqabeGadaaakeaacqWGmbatcqGGOaakcqaHrpGscqGGPaqkcqGH9aqpdaWfqaqaaiGbc2gaTjabcggaHjabcIha4bWcbaGafuiMdeLbaebaaeqaaOGagiiuaaLaeiOCaiNaeiikaGIaeqy0dOKaeiilaWIafuiMdeLbaebacqGGPaqkcqGGUaGlaaa@4168@

That is, we compute the maximum likelihood under the constraint that the value of ϑ is given. If we denote the overall maximum likelihood by *L*(Θ) = max_Θ _Pr(Θ), then the likelihood ratio

λ=−2ln⁡L(ϑ0)L(Θ),
 MathType@MTEF@5@5@+=feaafiart1ev1aaatCvAUfKttLearuWrP9MDH5MBPbIqV92AaeXatLxBI9gBaebbnrfifHhDYfgasaacH8akY=wiFfYdH8Gipec8Eeeu0xXdbba9frFj0=OqFfea0dXdd9vqai=hGuQ8kuc9pgc9s8qqaq=dirpe0xb9q8qiLsFr0=vr0=vr0dc8meaabaqaciaacaGaaeqabaqabeGadaaakeaaiiGacqWF7oaBcqGH9aqpcqGHsislcqaIYaGmcyGGSbaBcqGGUbGBdaWcaaqaaiabdYeamjabcIcaOiabeg9aknaaBaaaleaacqaIWaamaeqaaOGaeiykaKcabaGaemitaWKaeiikaGIaeuiMdeLaeiykaKcaaiabcYcaSaaa@3EEB@

under the hypothesis that ϑ = ϑ_0 _is distributed according to the *χ*^2 ^distribution with one degree of freedom. In order to find the 95% confidence interval of the parameter ϑ around its optimal value ϑ_0_, we find the value of ϑ for which the likelihood ratio (28) exceeds the value 3.84 (95%-percentile of the *χ*^2^(1) distribution). This value is found numerically using Ridder's method [50].

## Authors' contributions

LC contributed to the development of the probabilistic model, developed the EM algorithm, and drafted the manuscript; IBR collected the data and contributed to the development of the probabilistic model; YIW contributed to the development of the probabilistic model; EVK conceived of the study, provided the biological interpretation of the results, and finalized the manuscript. All authors read and approved the final manuscript.

## Supplementary Material

Additional file 1 Maximum likelihood estimation of the model parameters. Each parameter is associated with three values – the lower 95% confidence bound, the optimal value, and the upper 95% confidence bound. The confidence intervals were computed using the profile likelihood technique. Click here for file

Additional file 2 Comparison of the number of shared intron positions with the number expected by chance. All pairs of species (20), where the number of shared intron positions is not significantly greater than the number expected by chance alone. Click here for file

Additional file 3 Pattern-by-pattern analysis of parallel gains. The order of species in each pattern is Dicdi, Caeel, Strpu, Cioin, Danre, Galga, Homsa, Roden, Drome, Anoga, Cryne, Schpo, Sacce, Aspfu, Neucr, Arath, Orysa, Thepa, and Plafa. The frequency of a pattern is the number of times it was observed in our data. Click here for file

Additional file 4 The phylogenetic tree of eukaryotes used in the present study. Species and lineage abbreviations: Caeel (*Caenorhabditis elegans*), Strpu (*Strongylocentrotus purpuratus*), Cioin (*Ciona intestinalis*), Danre (*Danio rerio*), Galga (*Gallus gallus*), Homsa (*Homo sapiens*), roden (*Mus musculus *and *Rattus norvegicus *combined), Drome (*Drosophila melanogaster*), Anoga (*Anopheles gambiae*), cryne (*Cryptococcus neoformans*), Schpo (*Schizosaccharomyces pombe*), Sacce (*Saccharomyces cerevisiae*), Aspfu (*Aspergillus fumigatus*), Neucr (*Neurospora crassa*), Arath (*Arabidopsis thaliana*), Orysa (*Oryza sativa*), Thepa (*Theileria parva*), Plafa (*Plasmodium falciparum*), Dicdi (*Dictyostelium discoideum*), AME (Ancestor of Multicellular Eukaryotes). Click here for file
