# Supplementary material for: Patterns of intron gain and conservation in eukaryotic genes
Source: BMC Evol Biol. 2007 Oct 12;7:192. doi: 10.1186/1471-2148-7-192 (PMC2151770; doi:10.1186/1471-2148-7-192)
Supplement: Additional file 1 — Maximum likelihood estimation of the model parameters. Each parameter is associated with three values – the lower 95% confidence bound, the optimal value, and the upper 95% confidence bound. The confidence intervals were computed using the profile likelihood technique. [file 1471-2148-7-192-S1.doc]

**Additional Table**. Maximum likelihood estimation of the model parameters. Each parameter is associated with three values – the lower 95% confidence bound, the optimal value, and the upper 95% confidence bound. The confidence intervals were computed using the profile likelihood technique.

| $\pi_{0}$ | 0.9904 | 0.9969 | 0.9984 | $\varphi$(Caeel) | 0.7254 | 0.7546 | 0.7817 |
| --- | --- | --- | --- | --- | --- | --- | --- |
| $\theta$ | 0.0059 | 0.0280 | 0.0581 | $\varphi$(Strpu) | 0.1023 | 0.1295 | 0.1591 |
| $\varphi$(AME) | 0.4812 | 0.5740 | 0.6492 | $\varphi$(Cioin) | 0.4819 | 0.5092 | 0.5363 |
| $\varphi$(DicdiUnikonts) | 0.0158 | 0.1117 | 0.2112 | $\varphi$(Danre) | 0.0166 | 0.0291 | 0.0428 |
| $\varphi$(Unikonts) | 0.0000 | 0.0228 | 0.0914 | $\varphi$(Galga) | 0.0374 | 0.0502 | 0.0640 |
| $\varphi$(Metazoa) | 0.0488 | 0.1064 | 0.1614 | $\varphi$(Homsa) | 0.0280 | 0.0372 | 0.0479 |
| $\varphi$(Coelomata) | 0.0000 | 0.0047 | 0.0623 | $\varphi$(Drome) | 0.3091 | 0.3729 | 0.4392 |
| $\varphi$(Deuterostomia) | 0.0000 | 0.0297 | 0.0677 | $\varphi$(Anoga) | 0.3691 | 0.4319 | 0.4957 |
| $\varphi$(Diptera) | 0.6690 | 0.7090 | 0.7446 | $\varphi$(Cryne) | 0.2454 | 0.3095 | 0.3762 |
| $\varphi$(Fungi) | 0.2315 | 0.3108 | 0.3837 | $\varphi$(Schpo) | 0.6636 | 0.7291 | 0.7880 |
| $\varphi$(Ascomycota) | 0.1952 | 0.3483 | 0.4637 | $\varphi$(Sacce) | 0.9641 | 0.9834 | 0.9941 |
| $\varphi$(ScAfNc) | 0.0000 | 0.4641 | 0.6195 | $\varphi$(Aspfu) | 0.0479 | 0.1035 | 0.1742 |
| $\varphi$(Magnoliophyta) | 0.3105 | 0.3970 | 0.4696 | $\varphi$(Neucr) | 0.3962 | 0.4665 | 0.5367 |
| $\varphi$(Chordata) | 0.0270 | 0.0486 | 0.0722 | $\varphi$(Arath) | 0.0030 | 0.0191 | 0.0407 |
| $\varphi$(Vertebrata) | 0.0184 | 0.0342 | 0.0531 | $\varphi$(Orysa) | 0.0027 | 0.0171 | 0.0373 |
| $\varphi$(Apicomplexa) | 0.0000 | 0.2914 | 0.7514 | $\varphi$(Thepa) | 0.1638 | 0.2883 | 0.4091 |
| $\varphi$(Pezizomycotina) | 0.0000 | 0.1423 | 0.5722 | $\varphi$(Plafa) | 0.6554 | 0.7353 | 0.7964 |
| $\varphi$(Amniota) | 0.0000 | 0.0000 | 0.0071 | $\varphi$(Roden) | 0.1270 | 0.1434 | 0.1609 |
| $\varphi$(Mammals) | 0.0000 | 0.0000 | 0.0029 | $\lambda_{\theta}$ | 0.0500 | 0.0500 | ∞ |
| $\varphi$(Dicdi) | 0.6932 | 0.7379 | 0.7787 | $\nu$ | 0.6313 | 0.8622 | 0.9279 |
| $\eta$ | 0.0470 | 0.1263 | 0.2383 | $\xi$(Caeel) | 0.0199 | 0.0804 | 0.1193 |
| $\xi$(AME) | 0.0000 | 0.3972 | 0.7890 | $\xi$(Strpu) | 0.0052 | 0.0386 | 0.0612 |
| $\xi$(DicdiUnikonts) | 0.0000 | 0.0609 | 0.3360 | $\xi$(Cioin) | 0.0139 | 0.1055 | 0.1596 |
| $\xi$(Unikonts) | 0.0337 | 0.3071 | 0.5932 | $\xi$(Danre) | 0.0031 | 0.0377 | 0.0597 |
| $\xi$(Metazoa) | 0.0547 | 0.3989 | 0.7275 | $\xi$(Galga) | 0.0070 | 0.0492 | 0.0788 |
| $\xi$(Coelomata) | 0.0000 | 0.0000 | 0.1769 | $\xi$(Homsa) | 0.0059 | 0.0530 | 0.0929 |
| $\xi$(Deuterostomia) | 0.1100 | 1.0000 | 1.0000 | $\xi$(Drome) | 0.0015 | 0.0134 | 0.0271 |
| $\xi$(Diptera) | 0.0044 | 0.0568 | 0.0964 | $\xi$(Anoga) | 0.0014 | 0.0212 | 0.0372 |
| $\xi$(Fungi) | 0.0115 | 0.0973 | 0.1972 | $\xi$(Cryne) | 0.0262 | 0.1030 | 0.1526 |
| $\xi$(Ascomycota) | 0.0500 | 0.5103 | 1.0000 | $\xi$(Schpo) | 0.0007 | 0.0061 | 0.0122 |
| $\xi$(ScAfNc) | 0.0000 | 0.0000 | 0.3274 | $\xi$(Sacce) | 0.0001 | 0.0010 | 0.0025 |
| $\xi$(Magnoliophyta) | 0.0308 | 0.1412 | 0.2076 | $\xi$(Aspfu) | 0.0012 | 0.0162 | 0.0332 |
| $\xi$(Chordata) | 0.0209 | 0.1821 | 0.3414 | $\xi$(Neucr) | 0.0042 | 0.0372 | 0.0582 |
| $\xi$(Vertebrata) | 0.0076 | 0.0565 | 0.1004 | $\xi$(Arath) | 0.0092 | 0.0687 | 0.1143 |
| $\xi$(Apicomplexa) | 0.0000 | 0.0000 | 0.0852 | $\xi$(Orysa) | 0.0122 | 0.0916 | 0.1483 |
| $\xi$(Pezizomycotina) | 0.0192 | 0.1526 | 0.2687 | $\xi$(Thepa) | 0.0134 | 0.0496 | 0.0788 |
| $\xi$(Amniota) | 0.0000 | 0.0000 | 0.0092 | $\xi$(Plafa) | 0.0015 | 0.0074 | 0.0136 |
| $\xi$(Mammals) | 0.0000 | 0.0008 | 0.0058 | $\xi$(Roden) | 0.0053 | 0.0474 | 0.0841 |
| $\xi$(Dicdi) | 0.0022 | 0.0084 | 0.0133 | $\lambda_{\eta}$ | 0.0600 | 0.7628 | ∞ |
