# Supplementary material for: Patterns of intron gain and conservation in eukaryotic genes
Source: BMC Evol Biol. 2007 Oct 12;7:192. doi: 10.1186/1471-2148-7-192 (PMC2151770; doi:10.1186/1471-2148-7-192)
Supplement: Additional file 2 — Comparison of the number of shared intron positions with the number expected by chance. All pairs of species (20), where the number of shared intron positions is not significantly greater than the number expected by chance alone. [file 1471-2148-7-192-S2.doc]

**Additional Table**. All pairs of species (20), where the number of shared intron positions is not significantly greater than the number expected by chance alone.

| **Species 1** | **Species 2** | **p-value** | **Observed number of shared positions** | **Expected number of shared positions** |
| --- | --- | --- | --- | --- |
| Dicdi | Sacce | 0.008 | 2 | 0.1 |
| Caeel | Sacce | 0.049 | 2 | 0.4 |
| Strpu | Sacce | 0.045 | 3 | 0.8 |
| Cioin | Sacce | 0.021 | 3 | 0.6 |
| Danre | Sacce | 0.011 | 4 | 0.9 |
| Galga | Sacce | 0.010 | 4 | 0.8 |
| Homsa | Sacce | 0.051 | 3 | 0.8 |
| Drome | Sacce | 0.014 | 2 | 0.2 |
| Anoga | Sacce | 0.013 | 2 | 0.2 |
| Cryne | Sacce | 0.002 | 4 | 0.5 |
| Sacce | Neucr | 0.001 | 3 | 0.2 |
| Sacce | Arath | 0.153 | 2 | 0.7 |
| Sacce | Orysa | 0.158 | 2 | 0.7 |
| Caeel | Thepa | 0.074 | 34 | 26.0 |
| Drome | Thepa | 0.015 | 22 | 13.1 |
| Sacce | Thepa | 0.297 | 1 | 0.4 |
| Caeel | Plafa | 0.036 | 13 | 7.3 |
| Sacce | Plafa | 1.000 | 0 | 0.1 |
| Aspfu | Plafa | 0.003 | 12 | 4.7 |
| Sacce | Roden | 0.038 | 3 | 0.7 |
