# Supplementary material for: Patterns of intron gain and conservation in eukaryotic genes
Source: BMC Evol Biol. 2007 Oct 12;7:192. doi: 10.1186/1471-2148-7-192 (PMC2151770; doi:10.1186/1471-2148-7-192)
Supplement: Additional file 3 — Pattern-by-pattern analysis of parallel gains. The order of species in each pattern is Dicdi, Caeel, Strpu, Cioin, Danre, Galga, Homsa, Roden, Drome, Anoga, Cryne, Schpo, Sacce, Aspfu, Neucr, Arath, Orysa, Thepa, and Plafa. The frequency of a pattern is the number of times it was observed in our data. [file 1471-2148-7-192-S3.doc]

**Additional Table**. Pattern-by-pattern analysis of parallel gains. The order of species in each pattern is Dicdi, Caeel, Strpu, Cioin, Danre, Galga, Homsa, Roden, Drome, Anoga, Cryne, Schpo, Sacce, Aspfu, Neucr, Arath, Orysa, Thepa, and Plafa. The frequency of a pattern is the number of times it was observed in our data.

| pattern | frequency | probability of parallel gain |
| --- | --- | --- |
| 0000001000000010000 | 1 | 99.6% |
| 0000000100000001000 | 1 | 98.3% |
| 0000100000000001000 | 1 | 97.0% |
| 0000100000000100000 | 1 | 96.7% |
| 0001000000000001000 | 1 | 96.7% |
| 0100000000000010000 | 1 | 96.6% |
| 0000000001000010000 | 1 | 96.2% |
| 0001000000000100000 | 1 | 95.7% |
| 0000000010000010000 | 1 | 94.9% |
| 0001000000000110000 | 1 | 93.8% |
| 0000100000100000000 | 1 | 92.7% |
| 0100000000000000100 | 2 | 92.7% |
| 0000010000000001100 | 1 | 92.3% |
| 0000001000000001100 | 1 | 92.0% |
| 0001000000100000000 | 5 | 89.2% |
| 0001000000000001100 | 11 | 87.5% |
| 0000000000000100100 | 1 | 87.5% |
| 0000100001000000000 | 2 | 87.2% |
| 0100000000000100000 | 3 | 86.6% |
| 0000000000000101000 | 1 | 86.1% |
| 0000111100000010000 | 2 | 80.6% |
| 0101000000000000000 | 12 | 79.9% |
| 0100000000010000000 | 1 | 75.8% |
| 0000000000100000100 | 3 | 75.3% |
| 0001000001000000000 | 1 | 75.0% |
| 0101000000000010000 | 1 | 74.8% |
| 0100000000000001100 | 20 | 74.6% |
| 1001000000000000000 | 1 | 74.5% |
| 0001100000000000000 | 1 | 73.4% |
| 0000000000100001000 | 4 | 73.2% |
| 0000000001000001100 | 2 | 72.8% |
| 0010000000000001000 | 1 | 72.3% |
| 0100000000100000000 | 6 | 72.2% |
| 0000111100000000100 | 2 | 71.5% |
| 0000000001100000000 | 1 | 70.0% |
| 0000111100000001000 | 1 | 69.3% |
| 0001000010000000000 | 1 | 69.3% |
| 0000000010000001100 | 3 | 68.2% |
| 0001000000000011100 | 1 | 66.9% |
| 0000000010100000000 | 1 | 64.3% |
| 0000100000100110000 | 1 | 64.1% |
| 0100000000010110000 | 1 | 64.1% |
| 0000000000000101100 | 4 | 64.0% |
| 0000000011000001100 | 3 | 59.9% |
| 0001000100010110000 | 1 | 59.5% |
| 0010000000000100000 | 3 | 58.1% |
| 0010111100000010000 | 1 | 57.6% |
| 0100100000100000000 | 1 | 57.4% |
| 0000111100000100000 | 2 | 54.4% |
| 0000000011100000000 | 1 | 54.4% |
| 0010010000000000000 | 2 | 51.1% |
| 0100000001000000000 | 2 | 50.8% |
| 1000000001000000000 | 1 | 50.0% |
| 0010110100000000100 | 1 | 49.3% |
| 0010111100000000100 | 2 | 49.3% |
| 0011111100000000100 | 3 | 49.3% |
| 0010111100000001000 | 1 | 46.7% |
| 0011101100000001000 | 1 | 46.7% |
| 0011111000000001000 | 1 | 46.7% |
| 0011111100000001000 | 1 | 46.7% |
| 0000111100000110000 | 1 | 46.6% |
| 0000000011010100000 | 1 | 45.5% |
| 0000000000100010000 | 4 | 45.5% |
| 0000000000100001100 | 28 | 45.1% |
| 0101000000000001100 | 2 | 44.9% |
| 0010000000000001100 | 13 | 43.9% |
| 0100000010000000000 | 4 | 43.7% |
| 0110111100000010000 | 2 | 43.6% |
| 0001110000000100000 | 1 | 43.6% |
| 0010111011000010000 | 1 | 43.1% |
| 0010100000000000000 | 2 | 42.6% |
| 0000011100000001100 | 1 | 40.6% |
| 0000111000000001100 | 2 | 40.6% |
| 0000111100000001100 | 13 | 40.6% |
| 0101000000100000000 | 1 | 39.5% |
| 0000100000100001100 | 1 | 39.4% |
| 1000000000000001000 | 1 | 37.8% |
| 0000111100010000000 | 1 | 37.7% |
| 0010000000100000000 | 16 | 36.4% |
| 0001111000000110000 | 1 | 35.9% |
| 0110111100000001000 | 1 | 35.4% |
| 0000010000010101100 | 1 | 35.3% |
| 0011111111000001000 | 1 | 34.9% |
| 0000000000110100100 | 2 | 33.8% |
| 0000101000100000000 | 1 | 33.8% |
| 0000000000100111000 | 1 | 33.6% |
| 0000101100100000000 | 1 | 33.0% |
| 0000111000100000000 | 1 | 33.0% |
| 0000111100100000000 | 7 | 33.0% |
| 0100000011000000000 | 1 | 32.7% |
| 0001000000100001100 | 1 | 31.7% |
| 0001011100000001100 | 2 | 30.9% |
| 0001101100000001100 | 1 | 30.9% |
| 0001111000000001100 | 1 | 30.9% |
| 0001111100000001100 | 7 | 30.9% |
| 0010011100000100000 | 1 | 28.6% |
| 0010110100000100000 | 1 | 28.6% |
| 0010111100000100000 | 1 | 28.6% |
| 0011110100000100000 | 1 | 28.5% |
| 0011111100000100000 | 5 | 28.5% |
| 0010000000010110000 | 1 | 28.4% |
| 0001010000110110000 | 1 | 27.2% |
| 0000111100010100000 | 1 | 25.6% |
| 0011000000000110000 | 1 | 24.7% |
| 0100000010000001100 | 2 | 23.6% |
| 0001111000100000000 | 1 | 23.4% |
| 0001111100100000000 | 8 | 23.4% |
| 0011110100000110000 | 1 | 22.2% |
| 0011000000000001100 | 2 | 21.3% |
| 1000000000100000000 | 5 | 20.0% |
| 0101111100000100000 | 1 | 19.7% |
| 0010011100000001100 | 5 | 19.2% |
| 0010101100000001100 | 1 | 19.2% |
| 0010110100000001100 | 3 | 19.2% |
| 0010111000000001100 | 3 | 19.2% |
| 0010111100000001100 | 39 | 19.2% |
| 0011011000000001100 | 1 | 19.2% |
| 0011011100000001100 | 2 | 19.2% |
| 0011111000000001100 | 10 | 19.2% |
| 0011111100000001100 | 32 | 19.2% |
| 1010000000000000000 | 2 | 19.1% |
| 0100000000100001100 | 2 | 18.4% |
| 0111111000000100000 | 1 | 18.2% |
| 0011110111000100000 | 1 | 17.8% |
| 0011111111000100000 | 1 | 17.8% |
| 0110111011000100000 | 1 | 17.6% |
| 1000111100000000000 | 2 | 16.9% |
| 0110000000000000000 | 12 | 16.5% |
| 0000000000100100000 | 14 | 16.4% |
| 0010111000010000000 | 1 | 15.8% |
| 0010111100010000000 | 4 | 15.8% |
| 0011111100010000000 | 2 | 15.8% |
| 0100011000000000000 | 1 | 15.2% |
| 0110000000000001100 | 2 | 15.0% |
| 0010111100000111000 | 1 | 14.9% |
| 0011000000100000000 | 2 | 14.9% |
| 0100111100000001100 | 2 | 14.5% |
| 0100111100000000000 | 11 | 14.2% |
| 0010000000100010000 | 1 | 14.1% |
| 0000111101000001100 | 1 | 13.8% |
| 0010000010000001100 | 1 | 13.5% |
| 0010110110000110000 | 1 | 13.3% |
| 0010111100100000100 | 1 | 13.3% |
| 0011111100100000100 | 1 | 13.3% |
| 0101111100000001100 | 1 | 13.2% |
| 1000000000000001100 | 9 | 13.1% |
| 0010001100100000000 | 1 | 13.1% |
| 0010110000100000000 | 2 | 13.0% |
| 0010101000100000000 | 2 | 12.9% |
| 0010101100100000000 | 1 | 12.9% |
| 0010111000100000000 | 3 | 12.9% |
| 0010111100100000000 | 32 | 12.9% |
| 0011110100100000000 | 1 | 12.9% |
| 0011111000100000000 | 5 | 12.9% |
| 0011111100100000000 | 23 | 12.9% |
| 0010000001000000000 | 2 | 12.6% |
| 0000000000100101100 | 2 | 12.5% |
| 0110111000000001100 | 1 | 12.2% |
| 0110111100000001100 | 7 | 12.2% |
| 0111011100000001100 | 1 | 12.2% |
| 0111101100000001100 | 1 | 12.2% |
| 0111111000000001100 | 2 | 12.2% |
| 0111111100000001100 | 6 | 12.2% |
| 0011101101000001100 | 1 | 12.1% |
| 0011111101000001100 | 2 | 12.1% |
| 0010111010000001100 | 1 | 12.0% |
| 0010111110000001100 | 1 | 12.0% |
| 0011111110000001100 | 3 | 12.0% |
| 0011111100100001000 | 1 | 12.0% |
| 0000010000100101100 | 1 | 11.9% |
| 0010111111000001100 | 1 | 11.9% |
| 0011111111000001100 | 1 | 11.9% |
| 0101111110000001100 | 1 | 11.8% |
| 0111101001000001100 | 1 | 11.8% |
| 0111111101000001100 | 1 | 11.8% |
| 0110111111000001100 | 1 | 11.8% |
| 0111111111000001100 | 1 | 11.8% |
| 0111000000100000100 | 1 | 11.8% |
| 0000000000100110000 | 10 | 11.7% |
| 0000000000100111100 | 1 | 11.5% |
| 0110111100010001000 | 1 | 11.0% |
| 1001111100000000000 | 2 | 11.0% |
| 0000000000110001100 | 1 | 10.8% |
| 0000111101000000000 | 3 | 10.7% |
| 0110111100100001000 | 1 | 10.5% |
| 0010101000100100100 | 1 | 10.3% |
| 0010111100100100100 | 1 | 10.3% |
| 0000000000110111100 | 1 | 10.2% |
| 0000110000000000000 | 2 | 10.2% |
| 0010001111100100100 | 1 | 10.1% |
| 0111111100110000100 | 1 | 10.1% |
| 0011000000000000000 | 18 | 9.9% |
| 0110000000100000000 | 2 | 9.6% |
| 0010000010000000000 | 4 | 9.3% |
| 0110111000010000000 | 1 | 9.2% |
| 0110111100010000000 | 2 | 9.2% |
| 0010101000010110000 | 1 | 9.1% |
| 0110110111010000000 | 1 | 8.8% |
| 0101101100000000000 | 1 | 8.1% |
| 0101111100000000000 | 7 | 8.1% |
| 0000111010000000000 | 1 | 7.9% |
| 0000111110000000000 | 3 | 7.9% |
| 0001111100000101100 | 2 | 7.5% |
| 0000000000110000000 | 7 | 7.5% |
| 0110111000100000000 | 1 | 7.4% |
| 0110111100100000000 | 6 | 7.4% |
| 0111011100100000000 | 2 | 7.4% |
| 0111111100100000000 | 2 | 7.4% |
| 1100000000010000000 | 1 | 7.3% |
| 0010111101100000000 | 1 | 7.3% |
| 0010000000100100000 | 1 | 7.2% |
| 0010111010100000000 | 1 | 7.2% |
| 0010111110100000000 | 4 | 7.2% |
| 0011111110100000000 | 2 | 7.2% |
| 0010110111100000000 | 1 | 7.1% |
| 0010111111100000000 | 3 | 7.1% |
| 0011111111100000000 | 3 | 7.1% |
| 0110111101100000000 | 1 | 7.0% |
| 0111110101100000000 | 1 | 7.0% |
| 0111111101100000000 | 3 | 7.0% |
| 0110111110100000000 | 1 | 7.0% |
| 0111111110100000000 | 1 | 7.0% |
| 0110111111100000000 | 1 | 7.0% |
| 0111110111100000000 | 1 | 7.0% |
| 0010000000100110000 | 1 | 6.5% |
| 0000111100100100000 | 2 | 6.3% |
| 0001111100000111100 | 1 | 6.0% |
| 0001101101000000000 | 1 | 6.0% |
| 0010000000100001100 | 2 | 5.9% |
| 0010000010010110000 | 1 | 5.8% |
| 1010001100000000000 | 1 | 5.7% |
| 0000011110010110000 | 1 | 5.7% |
| 1010101000000000000 | 1 | 5.6% |
| 1010101100000000000 | 1 | 5.6% |
| 1010111100000000000 | 5 | 5.6% |
| 1011111100000000000 | 1 | 5.6% |
| 0010000011000000000 | 3 | 5.6% |
| 0000000000010010000 | 3 | 5.4% |
| 0000011100100001100 | 2 | 5.3% |
| 0000111100100001100 | 4 | 5.3% |
| 0011000000000101100 | 1 | 5.3% |
| 0000000000000000011 | 65 | 5.2% |
| 0110111111010110000 | 1 | 4.8% |
| 0001001100000000000 | 1 | 4.8% |
| 0000011000000000000 | 1 | 4.7% |
| 0000111100110100000 | 1 | 4.7% |
| 0010111000000101100 | 1 | 4.7% |
| 0010111100000101100 | 1 | 4.7% |
| 0000111011000000000 | 1 | 4.7% |
| 0000111111000000000 | 1 | 4.7% |
| 1100000000000001100 | 1 | 4.4% |
| 0010001010000000000 | 1 | 4.3% |
| 0001111110000000000 | 2 | 4.3% |
| 0001111000100001100 | 1 | 4.0% |
| 0001111100100001100 | 2 | 4.0% |
| 0011111100100010000 | 1 | 4.0% |
| 0010111100000111100 | 1 | 3.9% |
| 0011111100000111100 | 1 | 3.9% |
| 0000000000110100000 | 2 | 3.8% |
| 0000000000110110000 | 3 | 3.8% |
| 1101110100000000000 | 1 | 3.6% |
| 1000111110000000000 | 1 | 3.5% |
| 0110011100000101100 | 1 | 3.5% |
| 0110111000000101100 | 1 | 3.5% |
| 0010111110000101100 | 1 | 3.4% |
| 1000000000100100000 | 1 | 3.3% |
| 0000101000000000000 | 1 | 3.3% |
| 0010111100010001100 | 1 | 3.2% |
| 0011111100010001100 | 1 | 3.2% |
| 1011000001000000000 | 1 | 3.2% |
| 1111111100000000000 | 2 | 3.2% |
| 1011111001000000000 | 1 | 3.2% |
| 1011111110000000000 | 1 | 3.1% |
| 1100111101000000000 | 1 | 3.1% |
| 1010111111000000000 | 1 | 3.1% |
| 1011111111000000000 | 1 | 3.1% |
| 0001111100110000000 | 2 | 3.1% |
| 1111111111000000000 | 1 | 3.1% |
| 0000000011110001100 | 1 | 3.0% |
| 0100011000100010000 | 1 | 3.0% |
| 0010111000100001100 | 3 | 3.0% |
| 0010111100100001100 | 16 | 3.0% |
| 0011011100100001100 | 1 | 3.0% |
| 0011111000100001100 | 1 | 3.0% |
| 0011111100100001100 | 12 | 3.0% |
| 0100111100010001100 | 1 | 2.9% |
| 0011100000000000000 | 1 | 2.9% |
| 0010000010010001100 | 1 | 2.8% |
| 1000000000110000000 | 2 | 2.8% |
| 1010011100000010000 | 1 | 2.8% |
| 0110000000100001100 | 1 | 2.7% |
| 0010111100010101100 | 1 | 2.7% |
| 0010111001010001100 | 1 | 2.6% |
| 0110101000000000000 | 2 | 2.6% |
| 0110011100000000000 | 3 | 2.6% |
| 0110101100000000000 | 1 | 2.6% |
| 0110110100000000000 | 1 | 2.6% |
| 0110111000000000000 | 4 | 2.6% |
| 0110111100000000000 | 31 | 2.6% |
| 0111110000000000000 | 1 | 2.6% |
| 0111011100000000000 | 1 | 2.6% |
| 0111101100000000000 | 3 | 2.6% |
| 0111110100000000000 | 2 | 2.6% |
| 0111111000000000000 | 6 | 2.6% |
| 0111111100000000000 | 37 | 2.6% |
| 0101100100100001100 | 1 | 2.6% |
| 0001110101100001100 | 1 | 2.6% |
| 0000111111100001100 | 1 | 2.5% |
| 0001111010100001100 | 1 | 2.5% |
| 0110011100100001100 | 1 | 2.5% |
| 0110111100100001100 | 2 | 2.5% |
| 0111111000100001100 | 1 | 2.5% |
| 0111111100100001100 | 2 | 2.5% |
| 0001111111100001100 | 2 | 2.5% |
| 0010111101100001100 | 2 | 2.5% |
| 0010111110100001100 | 3 | 2.5% |
| 0011111110100001100 | 1 | 2.5% |
| 0011111111100001100 | 2 | 2.5% |
| 0101111101100001100 | 1 | 2.5% |
| 0111111110100001100 | 1 | 2.5% |
| 0110111111100001100 | 1 | 2.5% |
| 0001111111000000000 | 6 | 2.5% |
| 0000111100100101100 | 1 | 2.5% |
| 0010000001010111100 | 1 | 2.5% |
| 0000111100100111100 | 1 | 2.4% |
| 0111111000010011100 | 1 | 2.4% |
| 0000111000110001100 | 1 | 2.4% |
| 0000111100110001100 | 1 | 2.4% |
| 0010111101010101100 | 1 | 2.4% |
| 0000000000001100000 | 1 | 2.3% |
| 0011001111100011100 | 1 | 2.2% |
| 0111111110100011100 | 1 | 2.2% |
| 0010111100100101100 | 1 | 2.1% |
| 0011111100100101100 | 1 | 2.1% |
| 0010111100100111100 | 1 | 2.1% |
| 0011111100100111100 | 2 | 2.1% |
| 0010111000110001100 | 1 | 2.1% |
| 0010111100110001100 | 2 | 2.1% |
| 0011101000110001100 | 1 | 2.1% |
| 0011111100110001100 | 2 | 2.1% |
| 0010111000110101100 | 1 | 2.1% |
| 0011101100110101100 | 2 | 2.1% |
| 0110000000110001100 | 1 | 2.1% |
| 0110111100100101100 | 1 | 2.1% |
| 0111111100100111100 | 1 | 2.1% |
| 0110111111100111100 | 1 | 2.1% |
| 0011111101110001100 | 1 | 2.1% |
| 0111111011110001100 | 1 | 2.1% |
| 0110111000110111100 | 1 | 2.1% |
| 1000000000000111100 | 2 | 1.9% |
| 1010101101000000100 | 1 | 1.9% |
| 0010110101000000000 | 2 | 1.9% |
| 0010111101000000000 | 4 | 1.9% |
| 0011111001000000000 | 3 | 1.9% |
| 0011111101000000000 | 11 | 1.9% |
| 0010101100100100000 | 1 | 1.8% |
| 0010110100100100000 | 1 | 1.8% |
| 0010111000100100000 | 2 | 1.8% |
| 0010111100100100000 | 3 | 1.8% |
| 0011111000100100000 | 1 | 1.8% |
| 1111111100000001000 | 1 | 1.7% |
| 0010110100100110000 | 1 | 1.6% |
| 0010111100100110000 | 1 | 1.6% |
| 0011111100100110000 | 3 | 1.6% |
| 1000101100010000000 | 1 | 1.5% |
| 1111011000100000100 | 1 | 1.5% |
| 0010111100110000000 | 2 | 1.4% |
| 1010000000100000000 | 1 | 1.4% |
| 0110000000100100000 | 1 | 1.3% |
| 0010011110000000000 | 1 | 1.3% |
| 0010111010000000000 | 2 | 1.3% |
| 0010111110000000000 | 11 | 1.3% |
| 0011011110000000000 | 1 | 1.3% |
| 0011101110000000000 | 1 | 1.3% |
| 0011111010000000000 | 2 | 1.3% |
| 0011111110000000000 | 15 | 1.3% |
| 0010111100110100000 | 1 | 1.3% |
| 0010111000110110000 | 1 | 1.3% |
| 0011111000110100000 | 1 | 1.3% |
| 0011111100110110000 | 1 | 1.3% |
| 1111111111100111000 | 1 | 1.3% |
| 0000001100000000000 | 1 | 1.3% |
| 1000111100100000000 | 1 | 1.2% |
| 0001100100000000000 | 1 | 1.1% |
| 0001110000000000000 | 1 | 1.1% |
| 1000000000100001100 | 6 | 1.1% |
| 0010000010100100000 | 1 | 1.1% |
| 0011000011000000000 | 1 | 1.1% |
| 0111000000100100000 | 1 | 1.0% |
| 1000111100000001100 | 1 | 0.9% |
| 0110111100100100000 | 1 | 0.9% |
| 0111111100100100000 | 1 | 0.9% |
| 0010111001100100000 | 1 | 0.9% |
| 0010111101100100000 | 1 | 0.9% |
| 1001111100100000000 | 2 | 0.9% |
| 0010011111100100000 | 1 | 0.9% |
| 0101111111100100000 | 1 | 0.9% |
| 0111111110100100000 | 1 | 0.9% |
| 0110111111100100000 | 1 | 0.9% |
| 0111111111100100000 | 1 | 0.9% |
| 1010111100000110000 | 1 | 0.9% |
| 0001101000000000000 | 1 | 0.9% |
| 0000000000010100000 | 3 | 0.9% |
| 0010100011100110000 | 1 | 0.8% |
| 0110111100100110000 | 1 | 0.8% |
| 0010111101100110000 | 1 | 0.8% |
| 0011111001100110000 | 1 | 0.8% |
| 0011111101100110000 | 1 | 0.8% |
| 0010111110100110000 | 1 | 0.8% |
| 0110111010100110000 | 1 | 0.8% |
| 0111111111100110000 | 1 | 0.8% |
| 0010001100000000000 | 2 | 0.8% |
| 0001011100000000000 | 2 | 0.8% |
| 0001101100000000000 | 3 | 0.7% |
| 0001110100000000000 | 1 | 0.7% |
| 0001111000000000000 | 11 | 0.7% |
| 0001111100000000000 | 63 | 0.7% |
| 0010101111000000000 | 1 | 0.7% |
| 0010111011000000000 | 3 | 0.7% |
| 0010111111000000000 | 9 | 0.7% |
| 0011110011000000000 | 1 | 0.7% |
| 0011011111000000000 | 1 | 0.7% |
| 0011111011000000000 | 2 | 0.7% |
| 0011111111000000000 | 15 | 0.7% |
| 0100111101000000000 | 1 | 0.7% |
| 0100111111110000000 | 1 | 0.7% |
| 1010111100010000000 | 1 | 0.7% |
| 1000000001000111100 | 1 | 0.7% |
| 0111101000110100000 | 1 | 0.7% |
| 0111111100111110000 | 1 | 0.7% |
| 1001111000000001100 | 1 | 0.7% |
| 0010111101110100000 | 1 | 0.7% |
| 0010111110110110000 | 1 | 0.7% |
| 0010111111110110000 | 1 | 0.6% |
| 1010111111000110000 | 1 | 0.6% |
| 0111000011110100000 | 1 | 0.6% |
| 0111111111110110000 | 1 | 0.6% |
| 1010111100100000000 | 4 | 0.6% |
| 1011110100100000000 | 1 | 0.6% |
| 1011111000100000000 | 1 | 0.6% |
| 1011111100100000000 | 2 | 0.6% |
| 1011111100010100000 | 1 | 0.6% |
| 1011111110010000000 | 1 | 0.6% |
| 1110111100100000000 | 1 | 0.5% |
| 1011110101100000000 | 1 | 0.5% |
| 1011111110100000000 | 1 | 0.5% |
| 1101111101100000000 | 1 | 0.5% |
| 1111111101100000000 | 1 | 0.5% |
| 1110111110100000000 | 1 | 0.5% |
| 1110111111100000000 | 1 | 0.5% |
| 1000111100100100000 | 1 | 0.5% |
| 0100111010000000000 | 1 | 0.5% |
| 0000000000010110000 | 12 | 0.5% |
| 1100000000100001100 | 1 | 0.5% |
| 1010111000000001100 | 1 | 0.4% |
| 1010111100000001100 | 3 | 0.4% |
| 1011111000000001100 | 1 | 0.4% |
| 1011111100000001100 | 7 | 0.4% |
| 1010111100100100000 | 1 | 0.4% |
| 1011111100100110000 | 1 | 0.4% |
| 1010111100110000000 | 1 | 0.4% |
| 1010011100110100000 | 1 | 0.4% |
| 1010111100110110000 | 1 | 0.4% |
| 1110111111100100000 | 1 | 0.4% |
| 1110111100100110000 | 1 | 0.4% |
| 1010111101100110000 | 1 | 0.4% |
| 1111111100110000000 | 1 | 0.4% |
| 1000101111110110000 | 1 | 0.4% |
| 1111111110110000000 | 1 | 0.4% |
| 1111111100110010000 | 1 | 0.4% |
| 1000000010100001100 | 1 | 0.4% |
| 1111111110110110000 | 1 | 0.4% |
| 1100111100000001100 | 1 | 0.4% |
| 1010000001000001100 | 1 | 0.4% |
| 0101110101000000000 | 1 | 0.4% |
| 0101111101000000000 | 1 | 0.4% |
| 1101111100000001100 | 1 | 0.4% |
| 1000101111000001100 | 1 | 0.4% |
| 1110111100000001100 | 1 | 0.4% |
| 1111111100000001100 | 1 | 0.4% |
| 1011111101000001100 | 1 | 0.4% |
| 1011111110000001100 | 3 | 0.4% |
| 1110111011000001100 | 1 | 0.4% |
| 1111111111000001100 | 1 | 0.4% |
| 1000000000110101100 | 1 | 0.3% |
| 1011110100000101100 | 1 | 0.3% |
| 1011111100000101100 | 1 | 0.3% |
| 0100110111000000000 | 1 | 0.3% |
| 1001111100100001100 | 1 | 0.3% |
| 1111111100000101100 | 1 | 0.3% |
| 1010111100010001100 | 1 | 0.3% |
| 1110000011000111100 | 1 | 0.3% |
| 1111111111000111100 | 1 | 0.3% |
| 1010011100100001100 | 1 | 0.3% |
| 1010111100100001100 | 2 | 0.3% |
| 1011111100100001100 | 1 | 0.3% |
| 1011011111010001100 | 1 | 0.3% |
| 1011111011010001100 | 1 | 0.3% |
| 1110111111010001100 | 1 | 0.3% |
| 1110111000100001100 | 1 | 0.3% |
| 1110111100100001100 | 1 | 0.3% |
| 1111111100100001100 | 2 | 0.3% |
| 1011111111100001100 | 2 | 0.3% |
| 1111111101100001100 | 1 | 0.3% |
| 1110111110100001100 | 1 | 0.3% |
| 1110111111100001100 | 1 | 0.3% |
| 1010111000100101100 | 1 | 0.3% |
| 1010111100100101100 | 1 | 0.3% |
| 1011111111101001100 | 1 | 0.3% |
| 1010110100100111100 | 1 | 0.3% |
| 1010111100110001100 | 1 | 0.3% |
| 1010111100110111100 | 2 | 0.3% |
| 0101111110000000000 | 1 | 0.3% |
| 1011111001100101100 | 1 | 0.3% |
| 1111111100100111100 | 1 | 0.3% |
| 1011111011100111100 | 1 | 0.3% |
| 1011111111100111100 | 1 | 0.3% |
| 1111111011100111100 | 1 | 0.3% |
| 1110111100110001100 | 1 | 0.3% |
| 1010111011110001100 | 1 | 0.3% |
| 1011111111110001100 | 1 | 0.3% |
| 1110111111110001100 | 1 | 0.3% |
| 1011111110110101100 | 1 | 0.3% |
| 1100111010110111100 | 1 | 0.3% |
| 1101111111110111100 | 1 | 0.3% |
| 1111111110110111100 | 1 | 0.3% |
| 1110110111111111100 | 1 | 0.3% |
| 0010100100000000000 | 2 | 0.2% |
| 0010110000000000000 | 2 | 0.2% |
| 0010011000000000000 | 3 | 0.2% |
| 0010101000000000000 | 8 | 0.2% |
| 0010011100000000000 | 5 | 0.2% |
| 0010101100000000000 | 10 | 0.2% |
| 0010110100000000000 | 9 | 0.2% |
| 0010111000000000000 | 29 | 0.2% |
| 0010111100000000000 | 186 | 0.2% |
| 0111000001000000000 | 1 | 0.2% |
| 0101110111000000000 | 1 | 0.1% |
| 0101111111000000000 | 1 | 0.1% |
| 0000000000000110000 | 95 | 0.1% |
| 0110110101000000000 | 1 | 0.1% |
| 0110111101000000000 | 4 | 0.1% |
| 0111111001000000000 | 2 | 0.1% |
| 0111111101000000000 | 2 | 0.1% |
| 0110111010000000000 | 2 | 0.1% |
| 0110111110000000000 | 3 | 0.1% |
| 0111001110000000000 | 1 | 0.1% |
| 0111111110000000000 | 4 | 0.1% |
| 0011100100000000000 | 2 | 0.1% |
| 0011011000000000000 | 1 | 0.1% |
| 0011101000000000000 | 5 | 0.1% |
| 0011011100000000000 | 7 | 0.1% |
| 0011101100000000000 | 4 | 0.1% |
| 0011110100000000000 | 8 | 0.1% |
| 0011111000000000000 | 28 | 0.1% |
| 0011111100000000000 | 167 | 0.1% |
| 0000000011000000000 | 39 | 0.1% |
| 0111000011000000000 | 1 | 0.1% |
| 0110111111000000000 | 5 | 0.0% |
| 0111111011000000000 | 1 | 0.0% |
| 0111111111000000000 | 5 | 0.0% |
| 0000011100000000000 | 4 | 0.0% |
| 0000000000000001100 | 729 | 0.0% |
| 0000101100000000000 | 8 | 0.0% |
| 0000000000011100000 | 1 | 0.0% |
| 0000110100000000000 | 2 | 0.0% |
| 0000111000000000000 | 15 | 0.0% |
| 0000111100000000000 | 119 | 0.0% |
