# Supplementary material for: Patterns of intron gain and conservation in eukaryotic genes
Source: BMC Evol Biol. 2007 Oct 12;7:192. doi: 10.1186/1471-2148-7-192 (PMC2151770; doi:10.1186/1471-2148-7-192)
Supplement: Additional file 4 — The phylogenetic tree of eukaryotes used in the present study. Species and lineage abbreviations: Caeel (Caenorhabditis elegans), Strpu (Strongylocentrotus purpuratus), Cioin (Ciona intestinalis), Danre (Danio rerio), Galga (Gallus gallus), Homsa (Homo sapiens), roden (Mus musculus and Rattus norvegicus combined), Drome (Drosophila melanogaster), Anoga (Anopheles gambiae), cryne (Cryptococcus neoformans), Schpo (Schizosaccharomyces pombe), Sacce (Saccharomyces cerevisiae), Aspfu (Aspergillus fumigatus), Neucr (Neurospora crassa), Arath (Arabidopsis thaliana), Orysa (Oryza sativa), Thepa (Theileria parva), Plafa (Plasmodium falciparum), Dicdi (Dictyostelium discoideum), AME (Ancestor of Multicellular Eukaryotes). [file 1471-2148-7-192-S4.doc]

**Additional Figure**. The phylogenetic tree of eukaryotes used in the present study. Species and lineage abbreviations: Caeel (Caenorhabditis elegans), Strpu (Strongylocentrotus purpuratus), Cioin (Ciona intestinalis), Danre (Danio rerio), Galga (Gallus gallus), Homsa (Homo sapiens), roden (Mus musculus and Rattus norvegicus combined), Drome (Drosophila melanogaster), Anoga (Anopheles gambiae), cryne (Cryptococcus neoformans), Schpo (Schizosaccharomyces pombe), Sacce (Saccharomyces cerevisiae), Aspfu (Aspergillus fumigatus), Neucr (Neurospora crassa), Arath (Arabidopsis thaliana), Orysa (Oryza sativa), Thepa (Theileria parva), Plafa (Plasmodium falciparum), Dicdi (Dictyostelium discoideum), AME (Ancestor of Multicellular Eukaryotes).
